# Switching of the solid-state guest selectivity: solvent-dependent selective guest inclusion in a crystalline macrocyclic boronic ester†
†Electronic supplementary information (ESI) available: Experimental details, characterization data, PXRD and single-crystal X-ray crystallographic data. CCDC 1005494–1005500. For ESI and crystallographic data in CIF or other electronic format see DOI: 10.1039/c5sc04766h


**DOI:** 10.1039/c5sc04766h

**Published:** 2016-06-16

**Authors:** Suguru Ito, Kosuke Ono, Kohei Johmoto, Hidehiro Uekusa, Nobuharu Iwasawa

**Affiliations:** a Department of Chemistry , Tokyo Institute of Technology and JST-CREST , 2-12-1, O-okayama , Meguro-ku , Tokyo 152-8551 , Japan . Email: niwasawa@chem.titech.ac.jp; b Department of Chemistry and Materials Science , Tokyo Institute of Technology and JST-CREST , 2-12-1, O-okayama , Meguro-ku , Tokyo 152-8551 , Japan

## Abstract

Selective inclusion of each of bicyclic and tricyclic aromatic guest molecules was realized utilizing the self-assembly of a crystalline benzothiadiazole-type macrocyclic boronic ester. The complete switching of the guest selectivity was achieved simply by changing the cosolvent used in the formation of the crystalline host–guest complexes. Both the association free energy of host–guest complexes and the stability of packing structure were found to play important roles for the determination of the guest selectivity in this system, and the critical role of solvent molecules in the crystalline state for the switching phenomena was clarified.

## Abstract 2

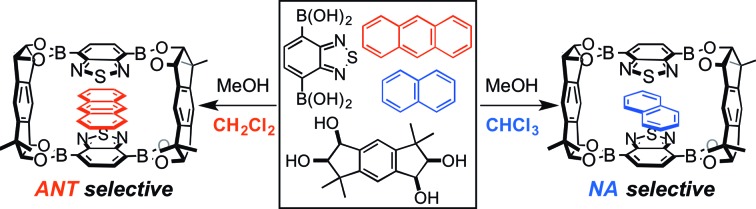
Switching of the inclusion of guest molecules was realized by the crystallization induced self-assembly of a benzothiadiazole-type macrocyclic boronic ester.

## Introduction

Crystalline host materials are often capable of separating a single guest molecule from a mixture of analogous compounds, which is practically useful for isolation or removal of a specific molecule.^1^–^6^ Extended crystalline frameworks such as zeolites^1^,^2^ and metal–organic frameworks (MOFs)^3^,^4^ have been widely explored as crystalline hosts for selective inclusion of either organic or inorganic molecules. Their guest selectivity is generally based on the selective recognition of the shape or electronic state of a suitable guest molecule by their framework. Although highly selective inclusion for a variety of guest molecules has been achieved by using crystalline frameworks, the alteration of the guest selectivity of crystalline host material still remains a challenging task.^7^ The development of a new crystalline host material is required to achieve this goal, which would contribute to the further growth of this field.

**Fig. 1:**
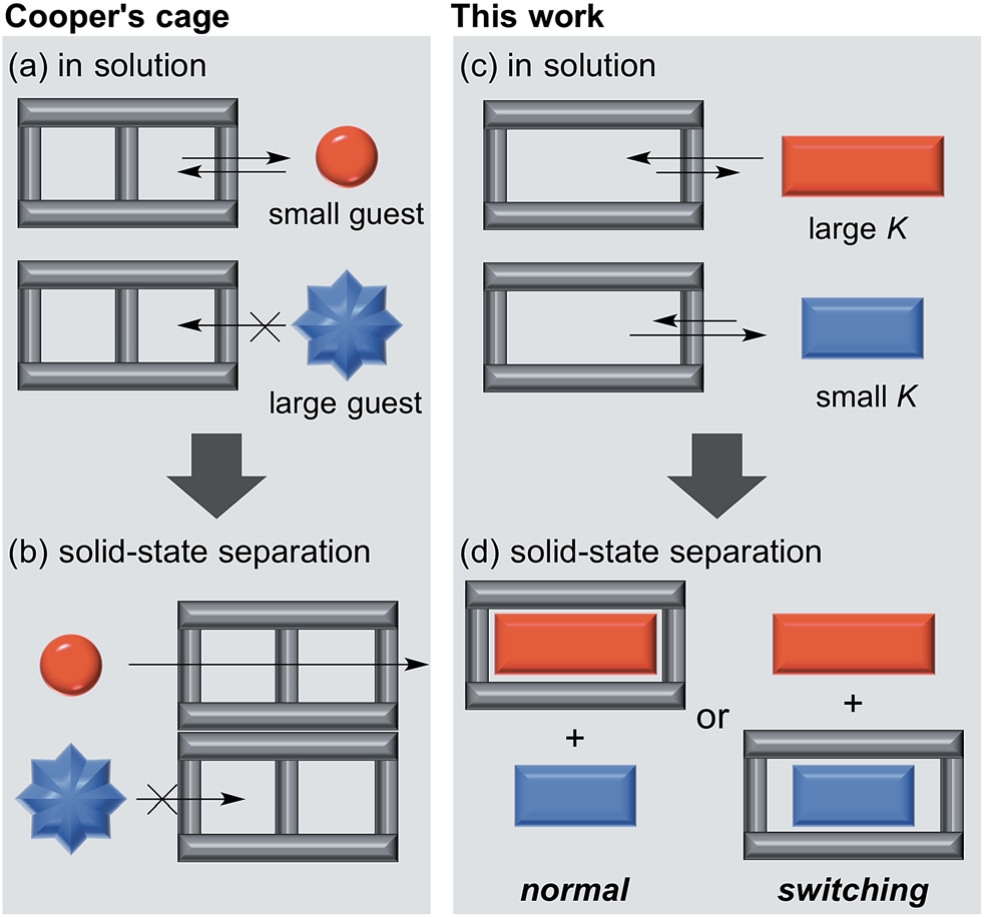
(a) Selective inclusion of a small guest molecule into the cavity of a cage compound with narrow windows in solution. (b) Solid-state chromatographic separation of guest molecules based on their shapes. (c) Association of a macrocyclic host and guest molecules with different association constants in solution. (d) Selective formation of a crystalline host–guest complex based on the association constants and the switching of the guest selectivity.

Recently, great advances have been made in the self-assembly of discrete, isolable architectures based on reversible bond formations, and a wide variety of self-assembled host compounds have been reported to form host–guest complexes with suitable guest compounds in solution state.^8^,^9^ Nevertheless, the solution-state host–guest behavior of the self-assembled architectures has not been utilized for the solid-state separation of guest mixtures until a recent report by Cooper *et al.*^10^ The reported imine-based, crystalline cage compound was able to separate guest mixtures based on their shape owing to their narrow windows where only small guest molecules can enter the cavity of the cage from the outside (Fig. 1a and b).^6^ On the other hand, the value of association constant (*K*) is generally utilized as an effective index to evaluate the relative affinity of different guest molecules for a host compound in the solution state. Although one can expect the selective inclusion of a guest molecule with a high *K* value into a crystalline host material, the relationship between the order of *K* values in solution and the solid-state separation of guest mixtures is not well documented as conventional crystalline host materials are often insoluble or dissociate into their components in solution. Herein, we demonstrate the selective guest separation by a self-assembled, crystalline macrocyclic boronic ester based on the *K* values of the discrete host boronic ester with guest molecules, and more importantly, a facile switching of guest selectivity in the crystalline state was achieved simply by changing the cosolvent used in the formation of the crystalline host–guest complexes (Fig. 1c and d). The main factor which contributed to the change of the selectivity was also clarified.

## Results and discussion

**Fig. 2:**
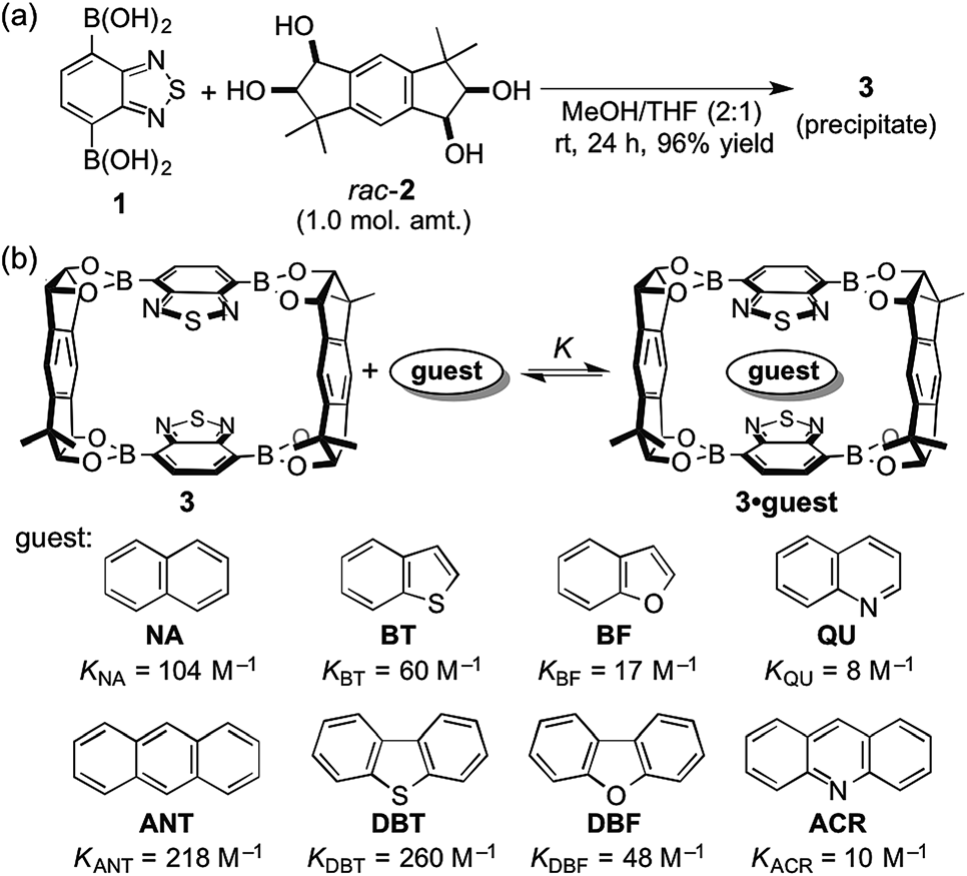
(a) Self-assembly of macrocyclic boronic ester **3** from **1** and *rac*-**2**. (b) Association constants of guest molecules with **3** in CDCl_3_ (determined by ^1^H NMR analysis).

We already reported the self-assembly of macrocyclic boronic esters simply by mixing a di- or tri-boronic acid with the indacene-type bis(1,2-diol) **2**, where precipitation with a guest molecule plays an important role for the selective formation of single host molecules.^6^,^11^ With this background, we envisaged that a macrocyclic boronic ester, which can associate with guest molecules in solution, would selectively precipitate with one guest molecule from a mixture of guest molecules in accordance with the order of the *K* values of the host–guest complexes in solution. 2,1,3-Benzothiadiazole-4,7-diboronic acid (**1**) was chosen as a component for the construction of such a macrocyclic boronic ester since 2,1,3-benzothiadiazole unit is known as an efficient electron-accepting unit, which was expected to recognize the electronic state of guest molecules.^12^ Benzothiadiazole-type macrocyclic boronic ester **3** composed of two molecules each of diboronic acid **1** and racemic bis(1,2-diol) **2** was obtained as a precipitate in 96% yield by the self-assembly of **1** with **2** in methanol/THF (Fig. 2a). When naphthalene (**NA**) was added to a deuterated chloroform solution of **3**, chemical shift values of **3** changed in ^1^H NMR spectrum of the mixture (Fig. S2†), indicating that there is an equilibrium between free **3** and host–guest complex with naphthalene (**3**·**NA**) in the solution state as expected. The binding stoichiometry of **3** with **NA** was estimated to be 1 : 1 by Job plot analysis (Fig. S1†), and the association constant (*K*) of **NA** with **3** was determined to be 104 M^–1^ by the ^1^H NMR experiment (Fig. 2b). Association constants of **3** for several bicyclic and tricyclic (hetero)aromatic compounds were also evaluated by ^1^H NMR analyses [Fig. 2b; benzothiophene (**BT**), benzofuran (**BF**), quinoline (**QU**), anthracene (**ANT**), dibenzothiophene (**DBT**), dibenzofuran (**DBF**), acridine (**ACR**)]. It should be noted that association constants of **3** for tricyclic compounds were higher than those for the corresponding bicyclic analogues (*K***_NA_** < *K***_ANT_**; *K***_BT_** < *K***_DBT_**; *K***_BF_** < *K***_DBF_**; *K***_QU_** < *K***_ACR_**).

**Fig. 3:**
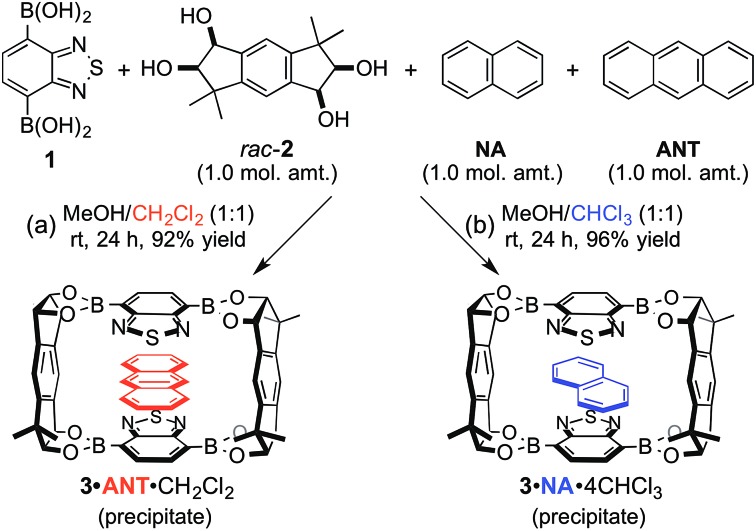
Solvent-dependent selective inclusion of guest molecules during the crystallization-induced self-assembly of macrocyclic boronic ester **3**. (a) Selective self-assembly of **3**·**ANT**·CH_2_Cl_2_ by using dichloromethane as cosolvent. (b) Selective self-assembly of **3**·**NA**·4CHCl_3_ by using chloroform as cosolvent.

Next, the self-assembly of **3** was carried out in the presence of naphthalene and anthracene aiming at the selective inclusion of one guest compound in **3** (Fig. 3). Considering the larger association constant (*K*) of **3** with anthracene (218 M^–1^) than with naphthalene (104 M^–1^), anthracene was expected to be included in **3** preferentially. In fact, anthracene-included **3** (**3**·**ANT**·CH_2_Cl_2_) selectively precipitated out in 92% yield as dichloromethane solvate when equimolar amounts of **1**, *rac*-**2**, naphthalene, and anthracene were mixed in methanol/dichloromethane (1 : 1) at room temperature for 24 h (Fig. 3a; **ANT**/**NA** ≥ 20 : 1 by ^1^H NMR). Powder X-ray diffraction (PXRD) analysis of the precipitate showed that the precipitate was an aggregate of microcrystals (Fig. S25†), and recrystallization of the precipitate afforded a single crystal, which was confirmed to be identical to that of the crystalline precipitate obtained by the self-assembly. X-ray crystallographic analysis exhibited that anthracene molecule is included in the center of the macrocyclic boronic ester **3** composed of two molecules of **1** and both enantiomers of *rac*-**2** (Fig. S28†).^13^ Other cosolvents (THF, Et_2_O, CH_3_CN, *etc.*) were also applicable instead of dichloromethane for the selective inclusion of anthracene, suggesting the importance of the difference of the association constants of the two guest compounds rather than cosolvent used in the self-assembly of the host–guest complex (Table S32†). Interestingly, the guest selectivity was dramatically changed and naphthalene-included **3** (**3**·**NA**·4CHCl_3_) was selectively obtained as a crystalline precipitate in 96% yield when chloroform was used as cosolvent (Fig. 3b; **NA**/**ANT** ≥ 20 : 1 by ^1^H NMR).

**Fig. 4:**
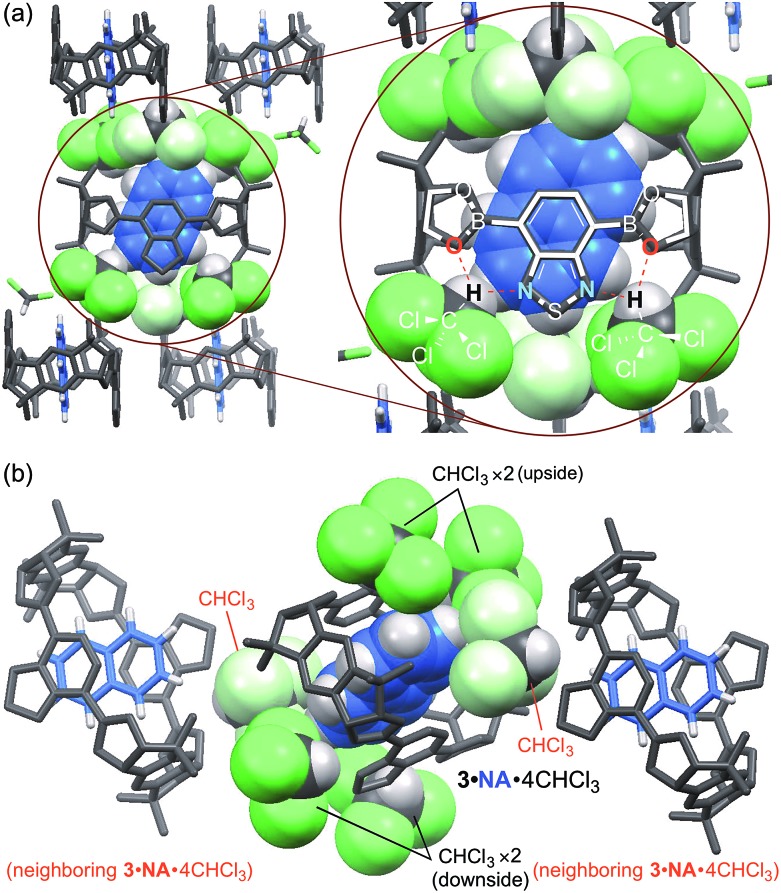
Single crystal structure of **3**·**NA**·4CHCl_3_. Included naphthalene (blue) and surrounding chloroform molecules (green) of central **3** are shown as space-filling model. (a) Top view. Each benzothiadiazole diboronic ester unit forms hydrogen bonds with two chloroform molecules. (b) Side view. Each naphthalene molecule included in **3** is surrounded by six chloroform molecules (four chloroform molecules forms hydrogen bonds with central **3** and the others with each neighboring **3**).

**Fig. 5:**
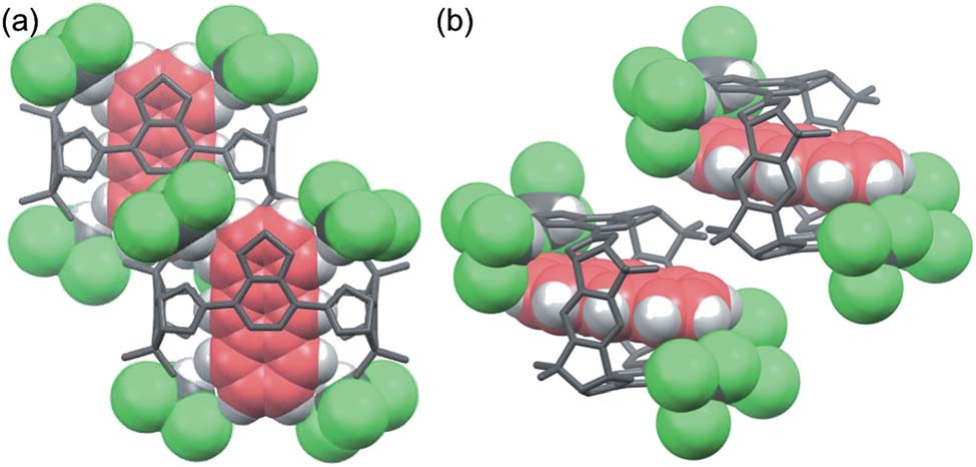
Single crystal structure of **3**·**ANT**·4CHCl_3_. Included anthracene (red) and surrounding chloroform molecules (green) of **3** are shown as space-filling model. (a) Top view. Each benzothiadiazole diboronic ester unit forms hydrogen bonds with two chloroform molecules. (b) Side view.

To reveal the role of chloroform, a single crystal of **3**·**NA**·4CHCl_3_ suitable for X-ray analysis was prepared by vapor diffusion of hexane into a chloroform solution of **3**·**NA**. As shown in Fig. 4a, each benzothiadiazole diboronic ester unit of **3**·**NA**·4CHCl_3_ forms hydrogen bonds with two chloroform molecules by the oxygen atom of the boronic ester moiety and the nitrogen atom of the benzothiadiazole moiety, and thus each **3**·**NA** is surrounded by four chloroform molecules (Fig. S22†).^13^ The naphthalene molecule is confined by six chloroform molecules owing to the packing structure of **3**·**NA**·4CHCl_3_ (*P*2_1_/*c*). That is, two chloroform molecules, forming hydrogen bonds with neighboring **3**·**NA**·4CHCl_3_, are additionally located just before and after the included naphthalene molecule (Fig. 4b). The molecular size of anthracene is considered to be too large to be included in this confined cavity (Fig. S35†). In fact, when the self-assembly of **3** was carried out in the presence of anthracene alone in methanol/chloroform, **3**·**ANT**·4CHCl_3_ of another crystal structure (*P*1[combining macron]) was obtained where each **3**·**ANT** also formed hydrogen bonds with four chloroform molecules (Fig. 5, S30, and S36†).^13^

These results indicate that **3**·**NA**·4CHCl_3_ in *P*2_1_/*c* crystal structure is more favorable than **3**·**ANT**·4CHCl_3_ in *P*1[combining macron] crystal structure. To obtain insight into this phenomenon, packing densities of guest-free **3**·4CHCl_3_ at 20 °C in *P*2_1_/*c* and *P*1[combining macron] crystal structures are calculated by virtually-removing the guest compounds inside **3** to be 1.311 g cm^–3^ and 1.268 g cm^–3^, respectively.^14^ Moreover, the self-assembly of **3** from **1** with *rac*-**2** in methanol/chloroform without addition of both naphthalene and anthracene gave chloroform-included **3**·4CHCl_3_ with *P*2_1_/*c* crystal structure in 96% yield (Fig. S26, S31 and S36†).^13^ These experimental results support the higher stability of **3**·4CHCl_3_ in *P*2_1_/*c* structure than that in *P*1[combining macron] structure, which should be the main factor for the selective inclusion of naphthalene by using chloroform.

We envisaged that the selective inclusion of each of the two guest compounds should also be applicable to several other combinations of bicyclic and tricyclic heteroaromatic compounds (Table 1). According to the higher *K* values of the tricyclic heteroaromatic compounds (**DBT**, **DBF**, and **ACR**) than those of the corresponding bicyclic ones (**BT**, **BF**, and **QU**), tricyclic heterocyclic compounds are considered to be included in **3** when dichloromethane is used as cosolvent. When the self-assembly of **3** was carried out using dichloromethane, **3**·**DBT**·CH_2_Cl_2_, **3**·**DBF**·CH_2_Cl_2_, and **3**·**ACR**·CH_2_Cl_2_ were selectively precipitated out in high yields in the presence of the corresponding bicyclic guest molecules, respectively (tricyclic/bicyclic ≥ 20 : 1 by ^1^H NMR). On the other hand, when the same experiments were carried out using chloroform as cosolvent, the reversal of the selectivity occurred and bicyclic compounds were selectively included in **3** as **3**·**BT**·4CHCl_3_, **3**·**BF**·4CHCl_3_, and **3**·**QU**·4CHCl_3_, respectively (bicyclic/tricyclic ≥ 20 : 1 by ^1^H NMR). Single crystal X-ray diffraction analyses of **3**·**guest**·4CHCl_3_ including these bicyclic heteroaromatic compounds revealed that the packing structures were identical to that of **3**·**NA**·4CHCl_3_ (*P*2_1_/*c*), indicating that the selective inclusion of the bicyclic compounds is attributed to the formation of the stable packing structure possessing the confined cavity created by **3** and chloroform molecules (Fig. S27 and S32–S34†).^13^

**Table 1:** Solvent-dependent selective inclusion of heteroaromatic guest compounds^*a*^

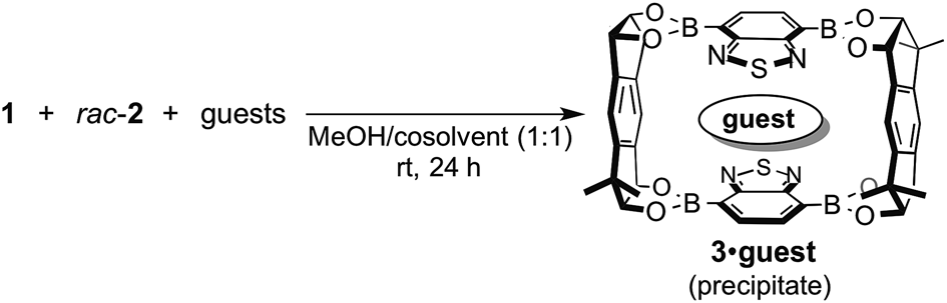			
Guests^*a*^	Cosolvent	Product	Yield (%)
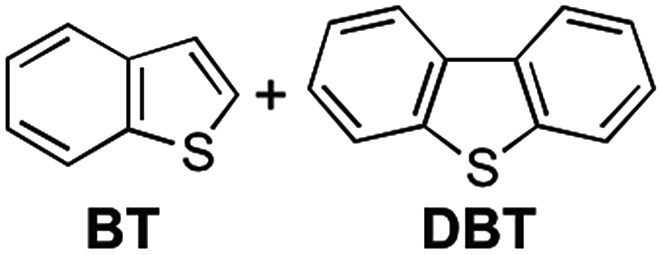	CH_2_Cl_2_	**3**·**DBT**·CH_2_Cl_2_	92
	CHCl_3_	**3**·**BT**·4CHCl_3_	96
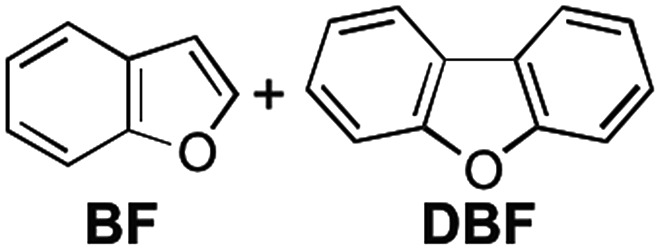	CH_2_Cl_2_	**3**·**DBF**·CH_2_Cl_2_	87
	CHCl_3_	**3**·**BF**·4CHCl_3_	96
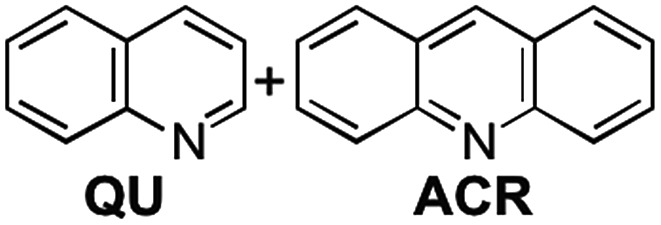	CH_2_Cl_2_	**3**·**ACR**·CH_2_Cl_2_	78
	CHCl_3_	**3**·**QU**·4CHCl_3_	96

^*a*^Diboronic acid **1**/racemic bis(1,2-diol) **2**/bicyclic heteroaromatic compound (**BT**, **BF**, or **QU**)/tricyclic heteroaromatic compound (**DBT**, **DBF**, or **ACR**) = 1 : 1 : 1 : 1.

## Conclusions

In conclusion, the selective inclusion of each of two kinds of guest molecules was achieved by using the crystalline macrocyclic boronic ester **3** containing benzothiadiazole moiety. The use of discrete self-assembled structure as crystalline host compound was found to have an advantage over extended porous frameworks such as MOFs in terms of the alteration of the guest selectivity. Although the crystalline-state guest selectivity generally coincided with the order of solution-state association energies of host–guest complexes when common organic solvents such as dichloromethane were used as the cosolvent, the guest selectivity was altered by using chloroform as the cosolvent, which could bind with host **3** by hydrogen bonding, thus forming the stable crystal structure with restricted space for guest inclusion. It should be noted that the solution-state association constant of a discrete self-assembled structure has not been utilized as an aid to understand the guest selectivity in crystalline-state to date. Moreover, the effective use of solvent effect for the switching of crystalline-state guest selectivity was demonstrated for the first time.^15^ We believe that these findings would not be limited to the present self-assembly system, thus should be applicable to other discrete self-assembled structures to realize a practical application of these structures as separating agents. Further investigations on the self-assembly of discrete boronic ester hosts, which can strongly bind wide variety of guest molecules other than planar aromatic and heteroaromatic compounds, are now in progress.

## Supplementary Material

Supplementary information

Crystal structure data
