# Supplementary material for: Switching of the solid-state guest selectivity: solvent-dependent selective guest inclusion in a crystalline macrocyclic boronic ester
Source: Chem Sci. 2016 Jun 16;7(9):5765–9. doi: 10.1039/c5sc04766h (PMC6054024; doi:10.1039/c5sc04766h)

Electronic Supplementary Material (ESI) for Chemical Science.
This journal is © The Royal Society of Chemistry 2016

Switching of the solid-state guest selectivity: solvent-dependent selective guest inclusion in a crystalline macrocyclic boronic ester

Suguru Ito, Kosuke Ono, Kohei Johmoto, Hidehiro Uekusa, and Nobuharu Iwasawa*

Department of Chemistry, Department of Chemistry and Materials Science
Tokyo Institute of Technology, and JST-CREST
O-okayama, Meguro-ku, Tokyo 152-8551, Japan
*e-mail: niwasawa@chem.titech.ac.jp

Table of Contents

1. General Methods …………………………………………………………………... S2
2. Experimental Procedure for the Self-Assembly of Macrocyclic Boronic Esters……... S3
3. Determination of Association Constants……………………… …………………... S4
4. Powder X-ray Diffraction Analysis………………………………………………… S53
5. X-ray Crystallographic Analysis …………………………………………………... S55
6. Miscellaneous Data………………………………………………………………… S69
References…………………………………………………………………………….. S72
1D and 2D NMR Spectra of Macrocyclic Boronic Esters………………………………. S73

1. General Methods

All operations were performed under air unless otherwise noted. ^{1}H and ^{13}C NMR spectra were recorded on a JEOL ECX-500 (500 MHz for ^{1}H and 125 MHz for ^{13}C) or on a JEOL ECX-400 (400 MHz for ^{1}H) spectrometer using $CDCl_3$ [tetramethylsilane (0 ppm) served as an internal standard in ^{1}H NMR and $CDCl_3$ (77.0 ppm) in ^{13}C NMR] as a solvent. Chemical shifts are expressed in parts per million (ppm). IR spectra were recorded on an FT/IR-460 plus (JASCO Co., Ltd.). Mass analyses (FAB^{+}) were performed on a JEOL JMS-700 mass spectrometer using NBA (3-nitrobenzyl alcohol) as matrix. Elemental analyses were performed on an elementar vario MICRO. Melting points were measured on an MPA100 (Stanford Research Systems) and are uncorrected. Dehydrated benzene, diethyl ether, and dichloromethane were purchased from Kanto Chemical Co., Inc. Tetrahydrofuran (THF) was purified by solvent purification system of Glass-Contour. Other solvents were distilled according to the usual procedures and stored over molecular sieves. 2,1,3-Benzothiadiazole-4,7-diboronic acid (**1**)[1] and racemic bis(1,2-diol) **2**[2] were synthesized according to the literature procedures.

2. Experimental Procedure for the Self-Assembly of Macrocyclic Boronic Esters

2-1. Self-assembly of macrocyclic boronic ester **3** (Figure 2a)

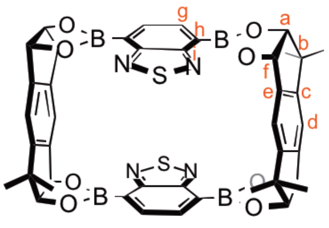

2,1,3-Benzothiadiazole-4,7-diboronic acid (**1**) (24.2 mg, 0.108 mmol) was added to a solution of *rac*-bis(1,2-diol) **2** (30.1 mg, 0.108 mmol) in MeOH/THF (2:1, 6.0 mL, 18 mM). The mixture immediately became homogeneous, and then a precipitate began to form within 3 min. After the mixture was stirred at room temperature for 24 h, **3** was obtained as a white powder by filtration followed by vacuum drying at 50 °C, for 3 h (45.0 mg, 97% yield). mp: 307–308 °C (decomposed); ^{1}H NMR (500 MHz, $CDCl_3$): δ 8.00 (s, 4H, H*g*), 7.30 (s, 4H, H*d*), 6.12 (d, *J* = 6.0 Hz, 4H, H*f*), 4.84 (d, *J* = 6.0 Hz, 4H, H*a*), 1.37 (s, 12H, Me), 1.18 (s, 12H, Me); ^{13}C NMR (125 MHz, $CDCl_3$): δ 156.7 (C*i*), 149.9 (C*c*), 141.2 (C*e*), 138.5 (C*g*), 125.5 (C*h*), 120.4 (C*d*), 89.0 (C*a*), 84.4 (C*f*), 47.4 (C*b*), 32.1 (C*Me*), 22.2 (C*Me*); IR (ATR) 2960, 1559, 1498, 1381, 1289, 1224, 1176, 1049, 1006, 887, 841, 788, 687 cm^{-1}; FAB-MS (*m*/*z*): $[M]^+$ Calcd. for $C_{44}H_{40}B_4N_4O_8S_2$, 860.2660; Found, 860.2651; Anal. Calcd for $C_{44}H_{40}B_4N_4O_8S_2$: C, 61.44; H, 4.69; N, 6.51; S, 7.46; Found: C, 61.38; H, 4.93; N, 6.30; S, 7.19.

2-2. General procedure for the selective self-assembly of **3•guest**•solvent (Figure 3, Table 1)

According to the same procedure used in the self-assembly of **3**, equimolar amounts of **1**, *rac*-**2**, bicyclic (hetero)aromatic compound (**NA**, **BT**, **BF**, or **QU**), and tricyclic (hetero)aromatic compound (**ANT**, **DBT**, **DBF**, or **ACR**) were stirred in MeOH/cosolvent (1:1) for 24 h to give **3•guest**•solvent as a precipitate. In all cases, the formation of **3** and the existence of guest compound were confirmed by ^{1}H NMR and FAB-MS analyses of the precipitates. Variable amount of cosolvent was observed in the ^{1}H NMR analyses. X-ray crystallographic analyses were carried out for the single crystals of **3•ANT**•CH_2Cl_2, **3•NA**•4$CHCl_3$, **3•ANT**•4$CHCl_3$, **3•CHCl$_3$**•4$CHCl_3$, **3•BT**•4$CHCl_3$, **3•BF**•4$CHCl_3$, and **3•QU**•4$CHCl_3$.

3. Determination of Association Constants

3-1. ^{1}H NMR analysis of **3•guest** for the determination of association constants

Association constant K of host-guest complex is defined

$$\mathrm{H} + \mathrm{G} \overset{K}{\rightleftharpoons} \mathrm{HG}$$

$$K = \frac{[\mathrm{HG}]}{[\mathrm{H}][\mathrm{G}]} = \frac{[\mathrm{HG}]}{([\mathrm{H}]_0-[\mathrm{HG}])([\mathrm{G}]_0-[\mathrm{HG}])} \quad \cdots (1)$$

$$\because [\mathrm{H}] = [\mathrm{H}]_0-[\mathrm{HG}],\ [\mathrm{G}] = [\mathrm{G}]_0-[\mathrm{HG}]$$

where [H], [G], and [HG] represent molar concentrations of the host, guest, and host-guest complex, respectively. $[\mathrm{H}]_0$ and $[\mathrm{G}]_0$ represent total concentrations of the host and guest molecules in the mixture.

Based on the maximum variation of chemical shift values of the host molecule ($\Delta\delta_{max}$), the changes in the chemical shift values of the host molecule ($\Delta\delta$) is defined as follows.

$$\Delta\delta = \frac{[\mathrm{HG}]\Delta\delta_{max}}{[\mathrm{H}]_0} \qquad \therefore\ [\mathrm{HG}] = \frac{[\mathrm{H}]_0\Delta\delta}{\Delta\delta_{max}} \quad \cdots (2)$$

Insertion of eq. (2) into eq. (1) leads to eq. (3).

$$\Delta\delta = \frac{\Delta\delta_{max}}{2K[\mathrm{H}]_0}\left[1+K[\mathrm{H}]_0+K[\mathrm{G}]_0-\{(1+K[\mathrm{H}]_0+K[\mathrm{G}]_0)^2-4K^2[\mathrm{H}]_0[\mathrm{G}]_0\}^{1/2}\right] \quad \cdots (3)$$

In eq. (3), unknown constants are K and $\Delta\delta_{max}$, and arbitrary constants are $[\mathrm{H}]_0$ and $[\mathrm{G}]_0$. The association constants K were estimated from plots of the observed changes in the chemical shift values of **3** ($\Delta\delta$) versus the initial concentration of guest molecule $[\mathrm{G}]_0$. The initial concentration of **3** ($[\mathrm{H}]_0$) was maintained to be 0.5 or 2.0 mM in $CDCl_3$ with 0.03% TMS (v/v) while that of guest molecule was varied. The results were analyzed by curve fitting using Delta Graph software (Red Rock Software Inc.). The K values are shown in 95% confidence intervals. The titration was carried out three times for each guest compound.

1) **3•NA**

Binding stoichiometry of **3** with **NA** was determined to be 1:1 by Job plot analysis of the ^{1}H NMR spectra of **3** with varying amount of **NA** in $CDCl_3$ with 0.03% TMS (v/v).

The NMR samples were prepared by the addition of different portions of **3** (2.0 mM) and **NA** (2.0 mM) so that the total concentration of added **3** and **NA** became 2.0 mM for each sample.

Table S1. Data table for Job plot of **3** with **NA** in $CDCl_3$

$[\mathbf{3}]_0$ / $([\mathbf{3}]_0+[\mathbf{NA}]_0)$	**3** (μL)	**NA** (μL)	δ of H_g (ppm)	Δδ of H_g	Δδ × $[\mathbf{3}]_0$ / $([\mathbf{3}]_0+[\mathbf{NA}]_0)$
1.00	600	0	7.9980	0	0.00000
0.90	540	60	7.9751	0.0229	0.02061
0.80	480	120	7.9536	0.0444	0.03552
0.70	420	180	7.9297	0.0683	0.04781
0.68	410	190	7.9253	0.0727	0.04968
0.66	400	200	7.9215	0.0765	0.05100
0.64	385	215	7.9152	0.0828	0.05313
0.62	370	230	7.9098	0.0882	0.05439
0.60	360	240	7.9057	0.0923	0.05538
0.54	325	275	7.8925	0.1055	0.05715
0.52	310	290	7.8864	0.1116	0.05766
0.50	300	300	7.8830	0.1150	0.05750
0.48	290	310	7.8798	0.1182	0.05713
0.46	275	325	7.8736	0.1244	0.05702
0.40	240	360	7.8604	0.1376	0.05504
0.38	230	370	7.8572	0.1408	0.05397
0.36	215	385	7.8515	0.1465	0.05250
0.33	200	400	7.8452	0.1528	0.05093
0.32	190	410	7.8421	0.1559	0.04937
0.30	180	420	7.8383	0.1597	0.04791
0.20	120	480	7.8162	0.1818	0.03636
0.10	60	540	7.7948	0.2032	0.02032

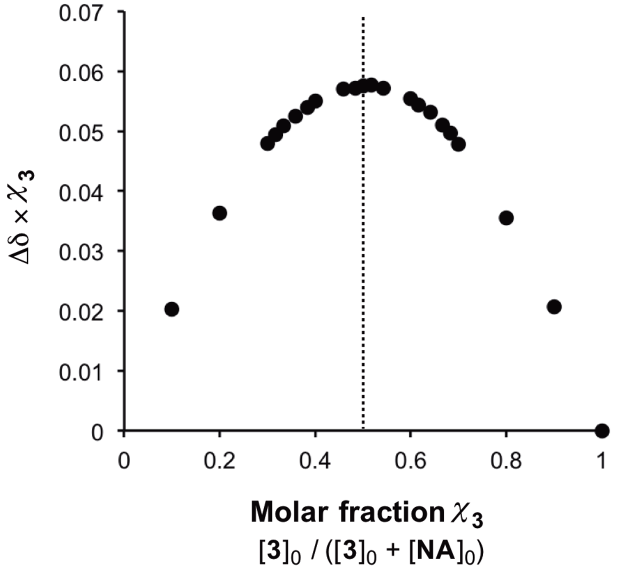

Figure S1. Job plot for NMR titration data of **3** and **NA**.

Table S2. Determination of association constant by the titration of **3** with NA

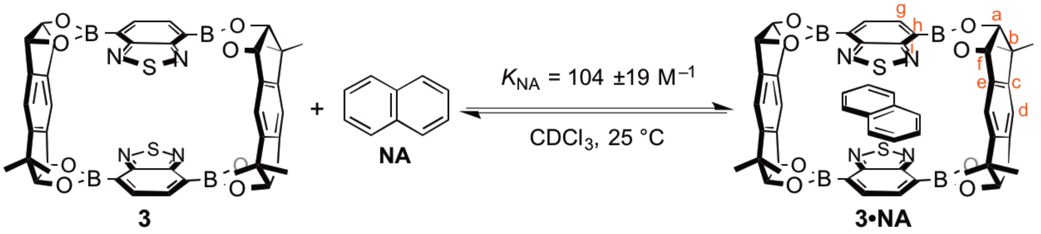

Entry	K_{NA} (M^{-1})
1st titration	97.5 ±12.2
2nd titration	109.7 ±19.1
3rd titration	106.0 ±15.0
Average	104 ±19

Table S3. Data tables for ^{1}H NMR titration of **3** with **NA**

1st titration

[G]$_0$ (M)	Δδ of H_{Me} 1.1801	Δδ of H_{Me} 1.3691	Δδ of H_a 4.8298	Δδ of H_f 6.1119	Δδ of H_d 7.2977	Δδ of H_g 7.9980
0.0030	0.0166	0.0469	-0.0154	0.0212	0.0899	-0.2617
0.0059	0.0269	0.0784	-0.0263	0.0355	0.1672	-0.4398
0.0088	0.0344	0.1002	-0.0332	0.0452	0.1924	-0.5417
0.0120	0.0395	0.1162	-0.0389	0.0526	0.2228	-0.6499
0.0180	0.0470	0.1385	-0.0469	0.0624	0.2663	-0.7782
0.0243	0.0521	0.1534	-0.0521	0.0693	0.2955	-0.8618
K (M^{-1})	101.8	96.4	89.9	96.9	108.4	91.6
$\Delta\delta_{max}$	0.07	0.22	-0.08	0.10	0.41	-1.26
χ^2	3.20E-07	2.54E-06	4.10E-07	4.92E-07	2.30E-04	1.68E-04
R^2	0.9996	0.9997	0.9996	0.9997	0.9915	0.9993

[H]$_0$ = 0.5 mM

K_{NA} = 97.5 ±12.2 M^{-1}

2nd titration

$[G]_0$ (M)	Δδ of H_{Me} 1.1804	Δδ of H_{Me} 1.3689	Δδ of H_a 4.8282	Δδ of H_f 6.1100	Δδ of H_d 7.2979	Δδ of H_g 7.9984
0.0028	0.0166	0.0498	-0.0154	0.0224	0.0951	-0.2715
0.0062	0.0275	0.0807	-0.0269	0.0367	0.1547	-0.4531
0.0090	0.0350	0.1025	-0.0343	0.0464	0.2194	-0.5750
0.0122	0.0401	0.1185	-0.0395	0.0539	0.2274	-0.6707
0.0180	0.0470	0.1380	-0.0463	0.0631	0.2675	-0.7698
0.0245	0.0527	0.1557	-0.0521	0.0711	0.2978	-0.8689
K (M^{-1})	120.8	120.6	96.8	100.4	116.1	103.7
$\Delta\delta_{max}$	0.07	0.21	-0.07	0.10	0.40	-1.21
χ^2	1.58E-06	2.14E-05	4.57E-07	2.57E-06	4.36E-04	2.94E-04
R^2	0.9984	0.9975	0.9995	0.9986	0.9843	0.9988

$[H]_0$ = 0.5 mM

K_{NA} = 109.7 ±19.1 M^{-1}

3rd titration

$[G]_0$ (M)	Δδ of H_{Me} 1.1810	Δδ of H_{Me} 1.3694	Δδ of H_a 4.8282	Δδ of H_f 6.1100	Δδ of H_d 7.2979	Δδ of H_g 7.9984
0.0031	0.0166	0.0504	-0.0171	0.0235	0.0980	-0.2858
0.0060	0.0269	0.0814	-0.0274	0.0373	0.1575	-0.4626
0.0092	0.0349	0.1037	-0.0343	0.0476	0.1993	-0.5825
0.0124	0.0395	0.1192	-0.0400	0.0545	0.2297	-0.6730
0.0175	0.0470	0.1375	-0.0469	0.0642	0.2698	-0.7847
0.0228	0.0510	0.1530	-0.0521	0.0688	0.2944	-0.8505
K (M^{-1})	113.1	118.1	95.0	105.3	100.3	104.4
$\Delta\delta_{max}$	0.07	0.21	-0.08	0.10	0.42	-1.21
χ^2	1.05E-06	8.11E-06	1.32E-06	1.26E-06	1.76E-05	1.04E-04
R^2	0.9987	0.9989	0.9985	0.9991	0.9993	0.9995

$[H]_0$ = 0.5 mM

K_{NA} = 106.0 ±15.0 M^{-1}

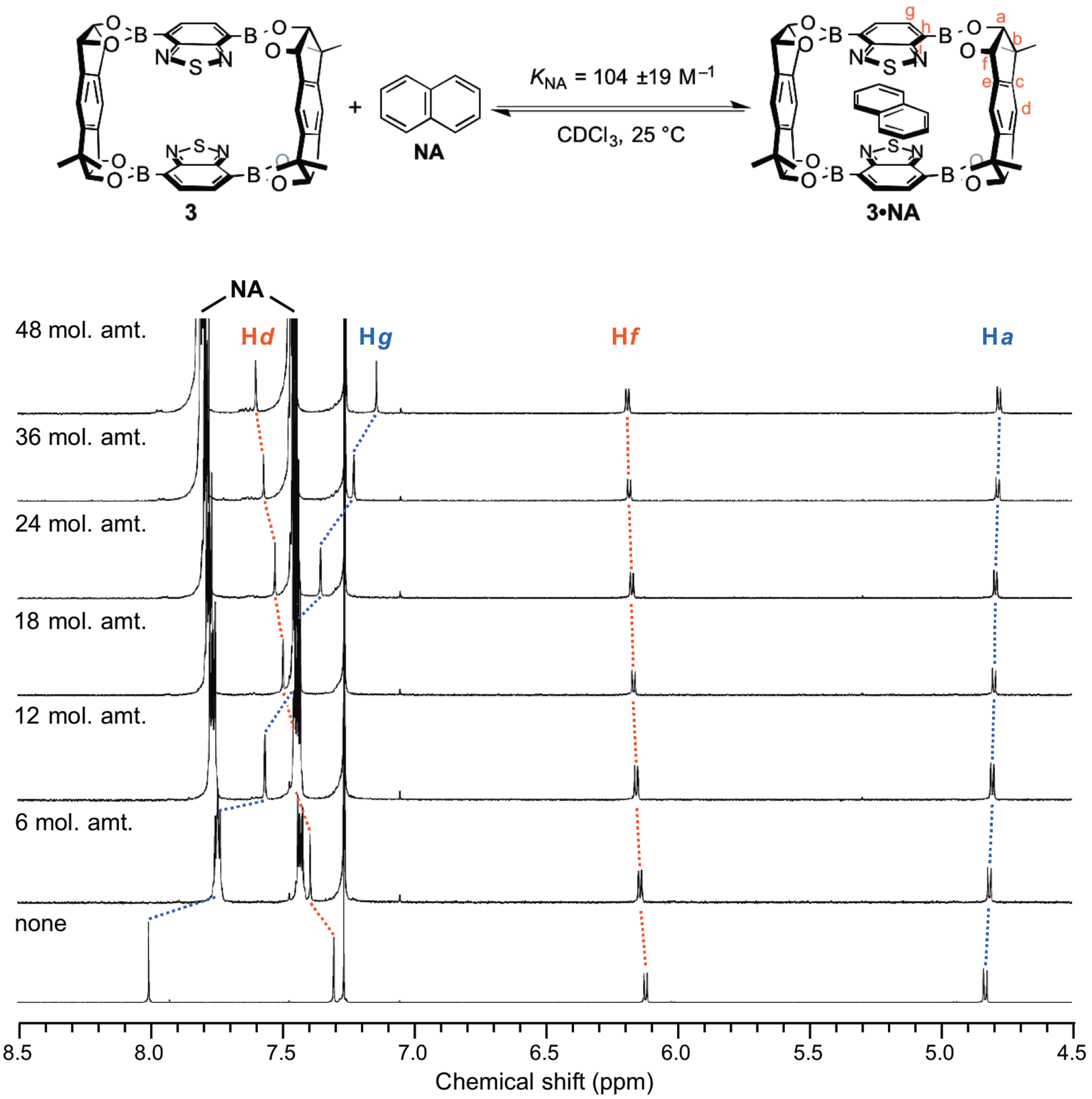

Figure S2. Partial ^{1}H NMR spectra of **3** with various amounts of naphthalene used for the determination of association constant K_{NA} (500 MHz, $CDCl_3$, 25 °C).

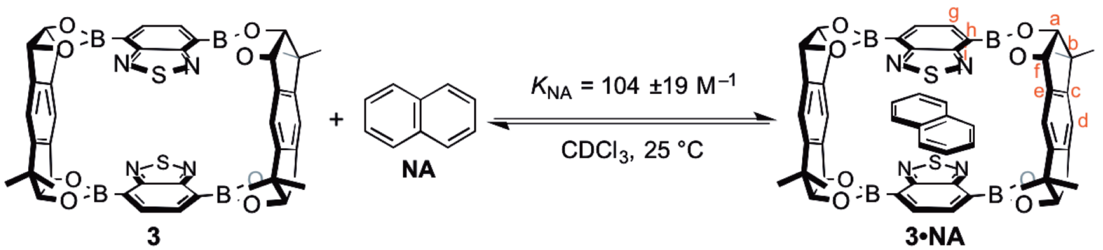

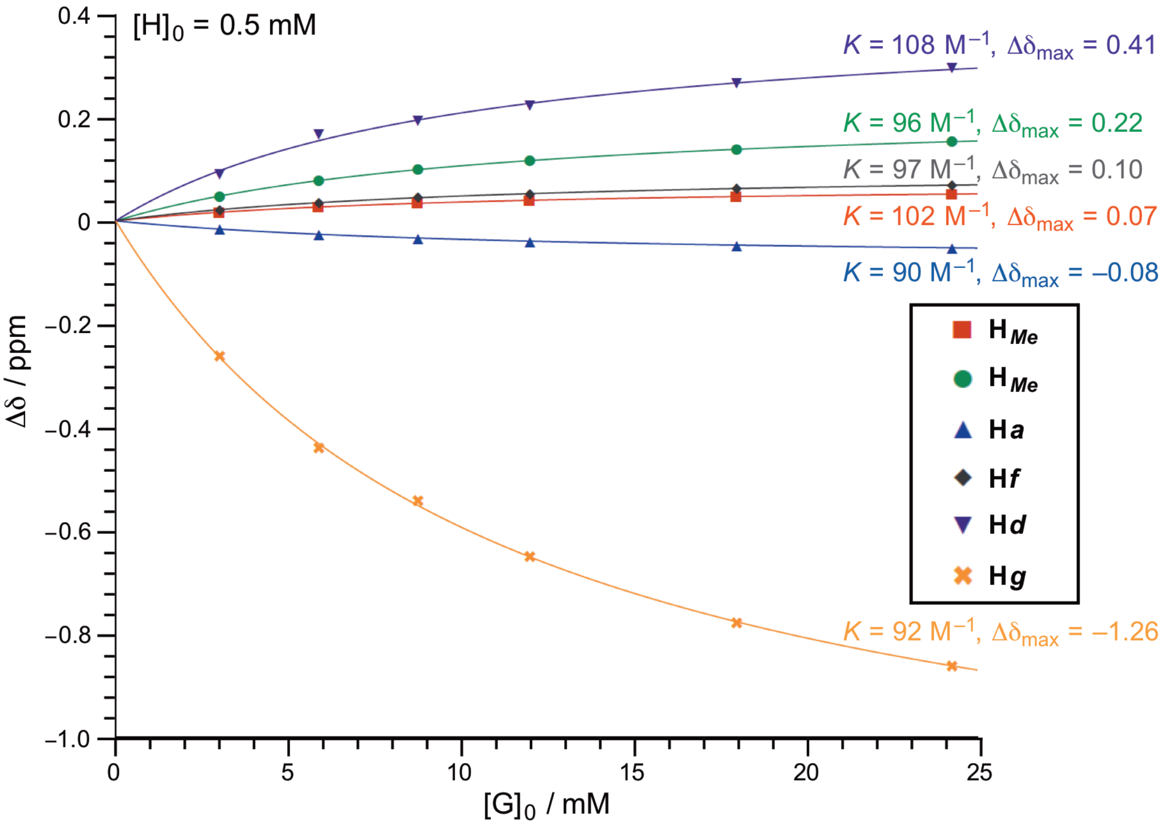

Figure S3. Changes in the chemical shift values Δδ of **3** plotted versus initial concentration of naphthalene $[G]_0$ and the corresponding fitting curve for the determination of association constant K_{NA}.

2) **3•BT**

Binding stoichiometry of **3** with **BT** was determined to be 1:1 by Job plot analysis of the ^{1}H NMR spectra of **3** with varying amount of **BT** in $CDCl_3$ with 0.03% TMS (v/v).

The NMR samples were prepared by the addition of different portions of **3** (2.0 mM) and **BT** (2.0 mM) so that the total concentration of added **3** and **BT** became 2.0 mM for each sample.

Table S4. Data table for Job plot of **3** with **BT** in $CDCl_3$

$[\mathbf{3}]_0$ / ($[\mathbf{3}]_0$+$[\mathbf{BT}]_0$)	**3** (μL)	**BT** (μL)	δ of H_g (ppm)	Δδ of H_g	Δδ ×$[\mathbf{3}]_0$ / ($[\mathbf{3}]_0$+$[\mathbf{BT}]_0$)
1.00	600	0	7.9980	0	0.00000
0.90	540	60	7.9864	0.0116	0.01044
0.80	480	120	7.9744	0.0236	0.01888
0.70	420	180	7.9631	0.0349	0.02443
0.68	410	190	7.9606	0.0374	0.02556
0.66	400	200	7.9589	0.0391	0.02607
0.64	385	215	7.9555	0.0425	0.02727
0.62	370	230	7.9530	0.0450	0.02775
0.60	360	240	7.9511	0.0469	0.02814
0.54	325	275	7.9448	0.0532	0.02882
0.52	310	290	7.9417	0.0563	0.02909
0.50	300	300	7.9392	0.0588	0.02940
0.48	290	310	7.9373	0.0607	0.02934
0.46	275	325	7.9347	0.0633	0.02901
0.40	240	360	7.9284	0.0696	0.02784
0.38	230	370	7.9259	0.0721	0.02764
0.36	215	385	7.9228	0.0752	0.02695
0.33	200	400	7.9202	0.0778	0.02593
0.32	190	410	7.9177	0.0803	0.02543
0.30	180	420	7.9165	0.0815	0.02445
0.20	120	480	7.9051	0.0929	0.01858
0.10	60	540	7.8938	0.1042	0.01042

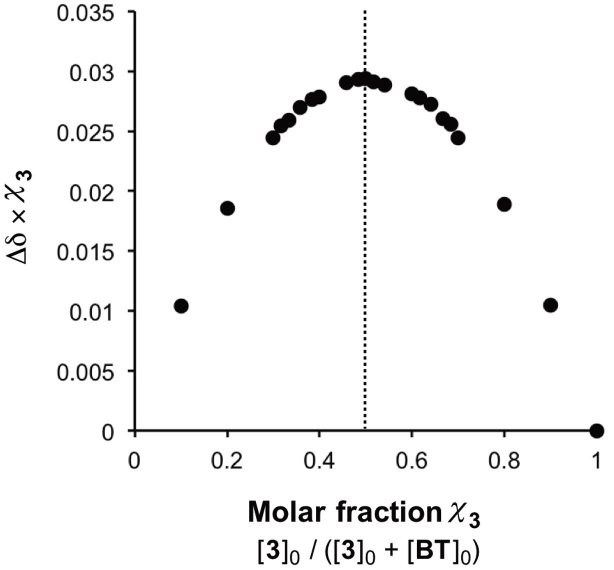

Figure S4. Job plot for NMR titration data of **3** and **BT**.

Table S5. Determination of association constant by the titration of **3** with **BT**

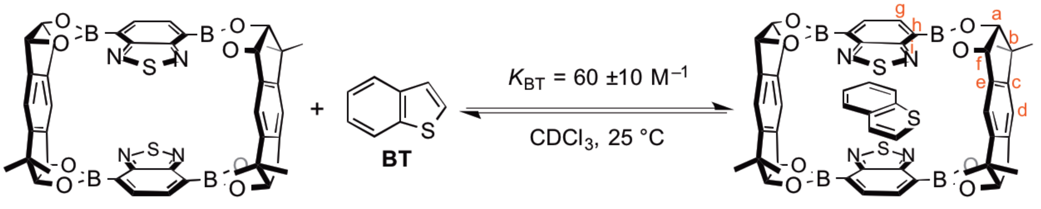

Entry	K_{BT} (M^{-1})
1st titration	59.9 ±8.8
2nd titration	60.6 ±7.8
3rd titration	58.4 ±12.8
Average	60 ±10

Table S6. Data tables for ^{1}H NMR titration of **3** with **BT**

1st titration

$[G]_0$ (M)	Δδ of H_{Me} 1.1804	Δδ of H_{Me} 1.3694	Δδ of H_a 4.8282	Δδ of H_f 6.1106	Δδ of H_d 7.2979	Δδ of H_g 7.9984
0.0047	0.0138	0.0424	-0.0148	0.0189	0.0791	-0.2234
0.0100	0.0224	0.0705	-0.0252	0.0315	0.1318	-0.3740
0.0211	0.0338	0.1054	-0.0366	0.0470	0.1959	-0.5550
0.0322	0.0395	0.1249	-0.0446	0.0556	0.2320	overlapped
0.0460	0.0441	0.1421	-0.0521	0.0630	0.2641	overlapped
0.0663	0.0470	0.1535	-0.0567	0.0670	0.2841	-0.8036
K (M^{-1})	66.2	58.9	51.3	61.9	59.9	61.0
$\Delta\delta_{max}$	0.06	0.19	-0.07	0.08	0.36	-1.00
χ^2	6.51E-07	6.55E-06	4.14E-06	1.51E-06	2.49E-05	6.96E-05
R^2	0.9992	0.9994	0.9971	0.9992	0.9993	0.9997

$[H]_0$ = 0.5 mM

K_{BT} = 59.9 ±8.8 M^{-1}

2nd titration

$[G]_0$ (M)	Δδ of H_{Me} 1.1804	Δδ of H_{Me} 1.3694	Δδ of H_a 4.8288	Δδ of H_f 6.1106	Δδ of H_d 7.2979	Δδ of H_g 7.9984
0.0049	0.0143	0.0436	-0.0154	0.0195	0.0814	-0.2303
0.0100	0.0229	0.0711	-0.0252	0.0321	0.1323	-0.3752
0.0206	0.0338	0.1060	-0.0378	0.0476	0.1970	-0.5561
0.0324	0.0395	0.1255	-0.0452	0.0556	0.2326	overlapped
0.0483	0.0447	0.1426	-0.0515	0.0630	0.2641	overlapped
0.0632	0.0470	0.1552	-0.0567	0.0676	0.2835	-0.7996
K (M^{-1})	67.1	58.2	54.1	62.8	60.6	60.8
$\Delta\delta_{max}$	0.06	0.20	-0.07	0.08	0.36	-1.01
χ^2	2.61E-07	8.48E-06	1.84E-06	8.25E-07	1.09E-05	1.20E-05
R^2	0.9997	0.9991	0.9986	0.9995	0.9997	0.9999

$[H]_0$ = 0.5 mM

K_{BT} = 60.6 ±7.8 M^{-1}

3rd titration

$[G]_0$ (M)	Δδ of H_{Me} 1.1793	Δδ of H_{Me} 1.3683	Δδ of H_a 4.8277	Δδ of H_f 6.1095	Δδ of H_d 7.2968	Δδ of H_g 7.9978
0.0053	0.0154	0.0452	-0.0149	0.0206	0.0830	-0.2319
0.0107	0.0252	0.0739	-0.0241	0.0343	0.1357	-0.3780
0.0208	0.0349	0.1071	-0.0367	0.0481	0.1981	-0.5567
0.0324	0.0412	0.1171	-0.0436	0.0573	0.2348	overlapped
0.0479	0.0452	0.1432	-0.0504	0.0641	0.2646	overlapped
0.0643	0.0487	0.1569	-0.0550	0.0687	0.2846	-0.8121
K (M^{-1})	69.7	55.7	48.9	62.7	58.3	55.1
$\Delta\delta_{max}$	0.06	0.20	-0.07	0.09	0.36	-1.04
χ^2	3.28E-07	1.02E-04	9.33E-07	2.22E-07	4.45E-06	2.78E-05
R^2	0.9996	0.9887	0.9992	0.9999	0.9999	0.9999

$[H]_0$ = 0.5 mM

K_{BT} = 58.4 ±12.8 M^{-1}

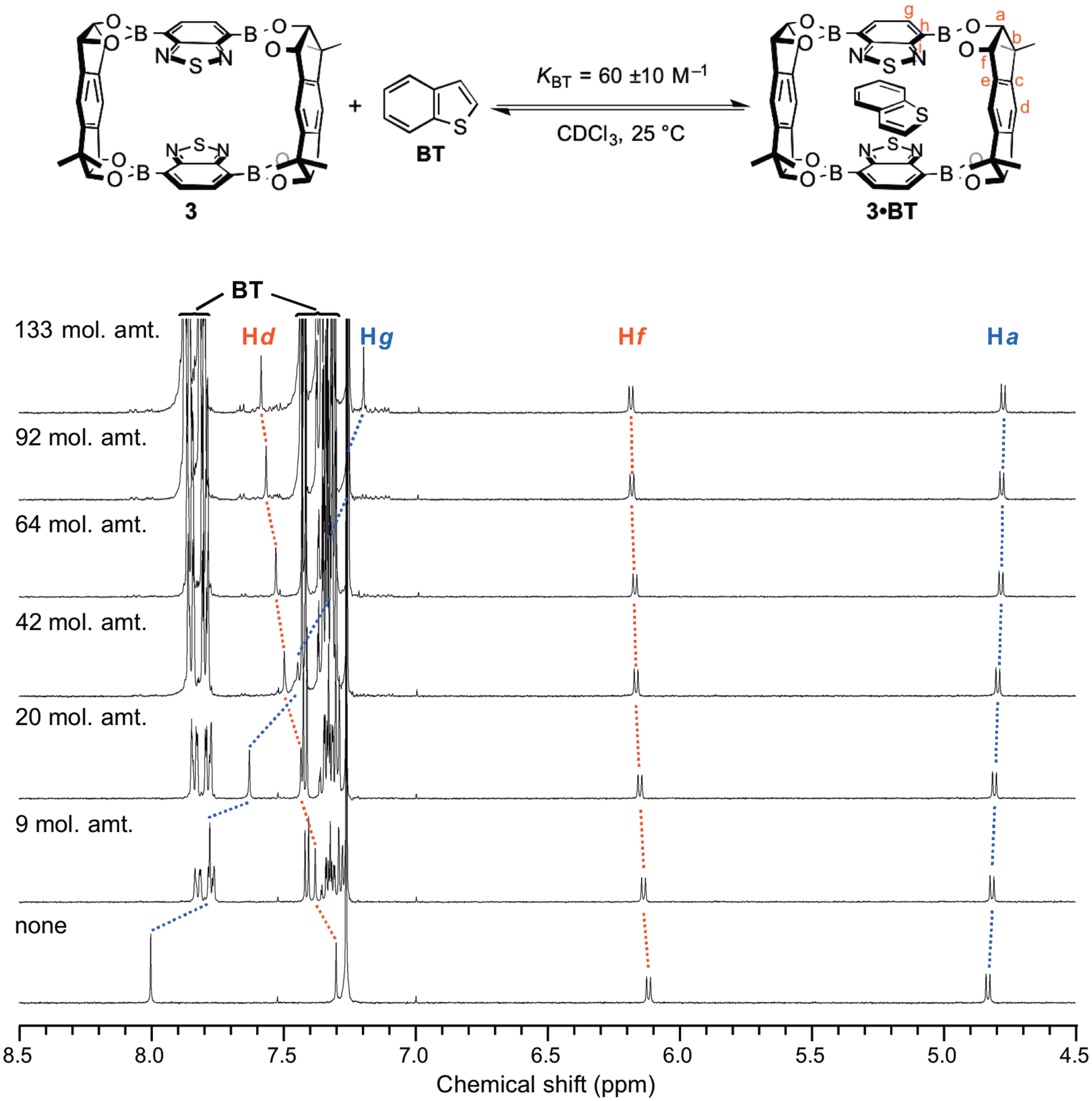

Figure S5. Partial ^{1}H NMR spectra of **3** with various amounts of benzothiophene used for the determination of association constant K_{BT} (400 MHz, $CDCl_3$, 25 °C).

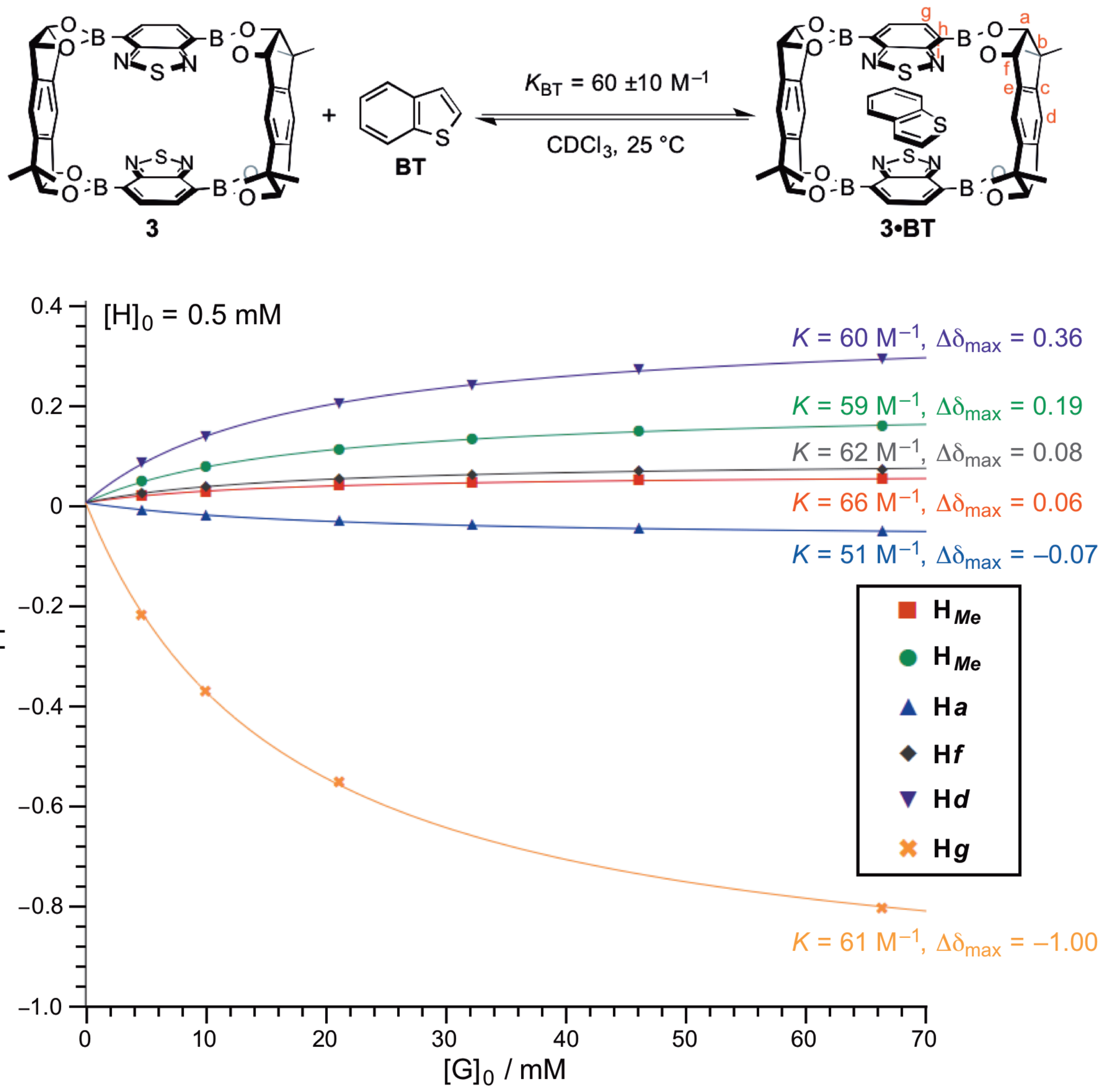

Figure S6. Changes in the chemical shift values Δδ of **3** plotted versus initial concentration of benzothiophene $[G]_0$ and the corresponding fitting curve for the determination of association constant K_{BT}.

3) **3•BF**

Binding stoichiometry of **3** with **BF** was determined to be 1:1 by Job plot analysis of the ^{1}H NMR spectra of **3** with varying amount of **BF** in $CDCl_3$ with 0.03% TMS (v/v).

The NMR samples were prepared by the addition of different portions of **3** (2.0 mM) and **BF** (2.0 mM) so that the total concentration of added **3** and **BF** became 2.0 mM for each sample.

Table S7. Data table for Job plot of **3** with **BF** in $CDCl_3$

$[\mathbf{3}]_0$ / ($[\mathbf{3}]_0$+$[\mathbf{BF}]_0$)	**3** (μL)	**BF** (μL)	δ of H_g (ppm)	Δδ of H_g	Δδ ×$[\mathbf{3}]_0$ / ($[\mathbf{3}]_0$+$[\mathbf{BF}]_0$)
1.00	600	0	7.9980	0	0.00000
0.90	540	60	7.9953	0.0027	0.00243
0.80	480	120	7.9927	0.0053	0.00424
0.70	420	180	7.9901	0.0079	0.00553
0.68	410	190	7.9896	0.0084	0.00574
0.66	400	200	7.9890	0.0090	0.00600
0.64	385	215	7.9883	0.0097	0.00622
0.62	370	230	7.9875	0.0105	0.00648
0.60	360	240	7.9871	0.0109	0.00654
0.54	325	275	7.9855	0.0125	0.00677
0.52	310	290	7.9848	0.0132	0.00682
0.50	300	300	7.9844	0.0136	0.00680
0.48	290	310	7.9839	0.0141	0.00682
0.46	275	325	7.9833	0.0147	0.00673
0.40	240	360	7.9817	0.0163	0.00652
0.38	230	370	7.9814	0.0166	0.00636
0.36	215	385	7.9808	0.0172	0.00616
0.33	200	400	7.9801	0.0179	0.00597
0.32	190	410	7.9798	0.0182	0.00576
0.30	180	420	7.9795	0.0185	0.00555
0.20	120	480	7.9763	0.0217	0.00434
0.10	60	540	7.9744	0.0236	0.00236

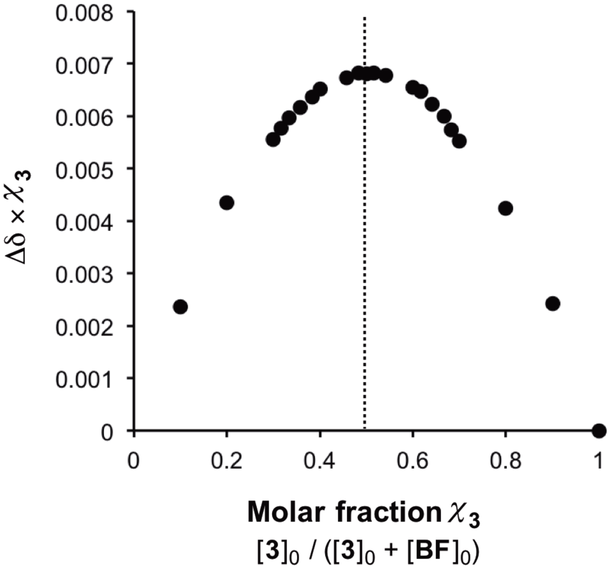

Figure S7. Job plot for NMR titration data of **3** and **BF**.

Table S8. Determination of association constant by the titration of **3** with **BF**

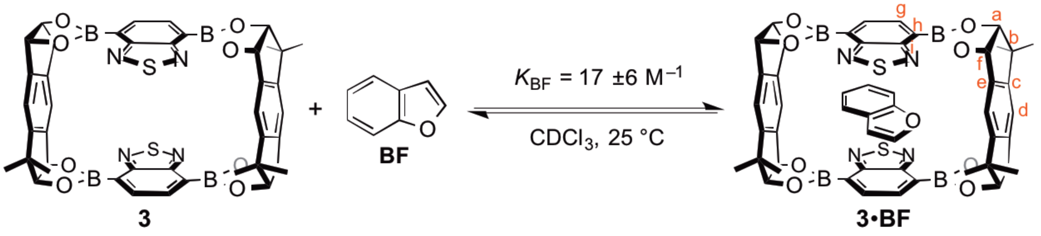

Entry	K_{BF} (M^{-1})
1st titration	15.6 ±5.8
2nd titration	17.7 ±5.6
3rd titration	16.5 ±6.5
Average	17 ±6

Table S9. Data tables for ^{1}H NMR titration of **3** with **BF**

1st titration

[G]$_0$ (M)	Δδ of H_{Me} 1.1801	Δδ of H_{Me} 1.3691	Δδ of H_a 4.8298	Δδ of H_f 6.1119	Δδ of H_d 7.2977	Δδ of H_g 7.998
0.0196	0.0132	0.0360	-0.0143	0.0177	0.0722	-0.2021
0.0390	0.0218	0.0607	-0.0246	0.0297	0.1220	-0.3401
0.0600	0.0269	0.0773	-0.0320	0.0378	0.1552	-0.4323
0.1020	0.0322	0.0979	-0.0418	0.0469	0.1981	-0.5529
0.1390	0.0372	0.1116	-0.0492	0.0532	0.2262	-0.6299
0.2100	0.0390	0.1237	-0.0572	0.0572	0.2514	-0.7008
K (M^{-1})	20.6	15.2	10.8	17.4	14.7	14.7
$\Delta\delta_{max}$	0.05	0.16	-0.08	0.07	0.33	-0.93
χ^2	2.57E-06	3.32E-06	1.47E-06	2.40E-06	1.29E-05	8.16E-05
R^2	0.9947	0.9994	0.9989	0.9980	0.9994	0.9995

[H]$_0$ = 2.0 mM

K_{BF} = 15.6 ±5.8 M^{-1}

2nd titration

$[G]_0$ (M)	$\Delta\delta$ of H_{Me} 1.1799	$\Delta\delta$ of H_{Me} 1.3689	$\Delta\delta$ of H_a 4.8282	$\Delta\delta$ of H_f 6.1100	$\Delta\delta$ of H_d 7.2979	$\Delta\delta$ of H_g 7.9978
0.0173	0.0012	0.0332	-0.0131	0.0161	0.0653	-0.1833
0.0394	0.0206	0.0572	-0.0229	0.0281	0.1146	-0.3213
0.0596	0.0269	0.0744	-0.0297	0.0367	0.1484	-0.4135
0.1072	0.0332	0.0956	-0.0400	0.0464	0.1638	-0.5278
0.1592	0.0366	0.1094	-0.0475	0.0522	0.2200	-0.6140
0.3200	0.0395	0.1220	-0.0549	0.0573	0.2469	-0.6879
K (M^{-1})	22.6	17.6	13.4	19.5	16.0	17.1
$\Delta\delta_{max}$	0.05	0.15	-0.07	0.07	0.29	-0.82
χ^2	3.34E-06	1.16E-05	2.02E-06	4.46E-06	5.86E-04	2.83E-04
R^2	0.9942	0.9980	0.9984	0.9965	0.9741	0.9984

$[H]_0$ = 2.0 mM

K_{BF} = 17.7 ±5.6 M^{-1}

3rd titration

$[G]_0$ (M)	$\Delta\delta$ of H_{Me} 1.1799	$\Delta\delta$ of H_{Me} 1.3689	$\Delta\delta$ of H_a 4.8277	$\Delta\delta$ of H_f 6.1100	$\Delta\delta$ of H_d 7.2973	$\Delta\delta$ of H_g 7.9978
0.0184	0.0126	0.0332	-0.0126	0.0167	0.0665	-0.1850
0.0392	0.0211	0.0578	-0.0224	0.0287	0.1157	-0.3219
0.0616	0.0263	0.0601	-0.0292	0.0367	0.1490	-0.4135
0.1098	0.0332	0.0956	-0.0395	0.0464	0.1650	-0.5278
0.1660	0.0360	0.1088	-0.0464	0.0516	0.2200	-0.6082
0.2960	0.0395	0.1214	-0.0544	0.0573	0.2469	-0.6867
K (M^{-1})	22.2	14.0	12.1	19.2	15.4	16.3
$\Delta\delta_{max}$	0.05	0.15	-0.07	0.07	0.30	-0.83
χ^2	8.45E-07	1.46E-04	1.77E-07	8.81E-07	5.60E-04	2.99E-05
R^2	0.9984	0.9755	0.9999	0.9993	0.9749	0.9998

$[H]_0$ = 2.0 mM

K_{BF} = 16.5 ±6.5 M^{-1}

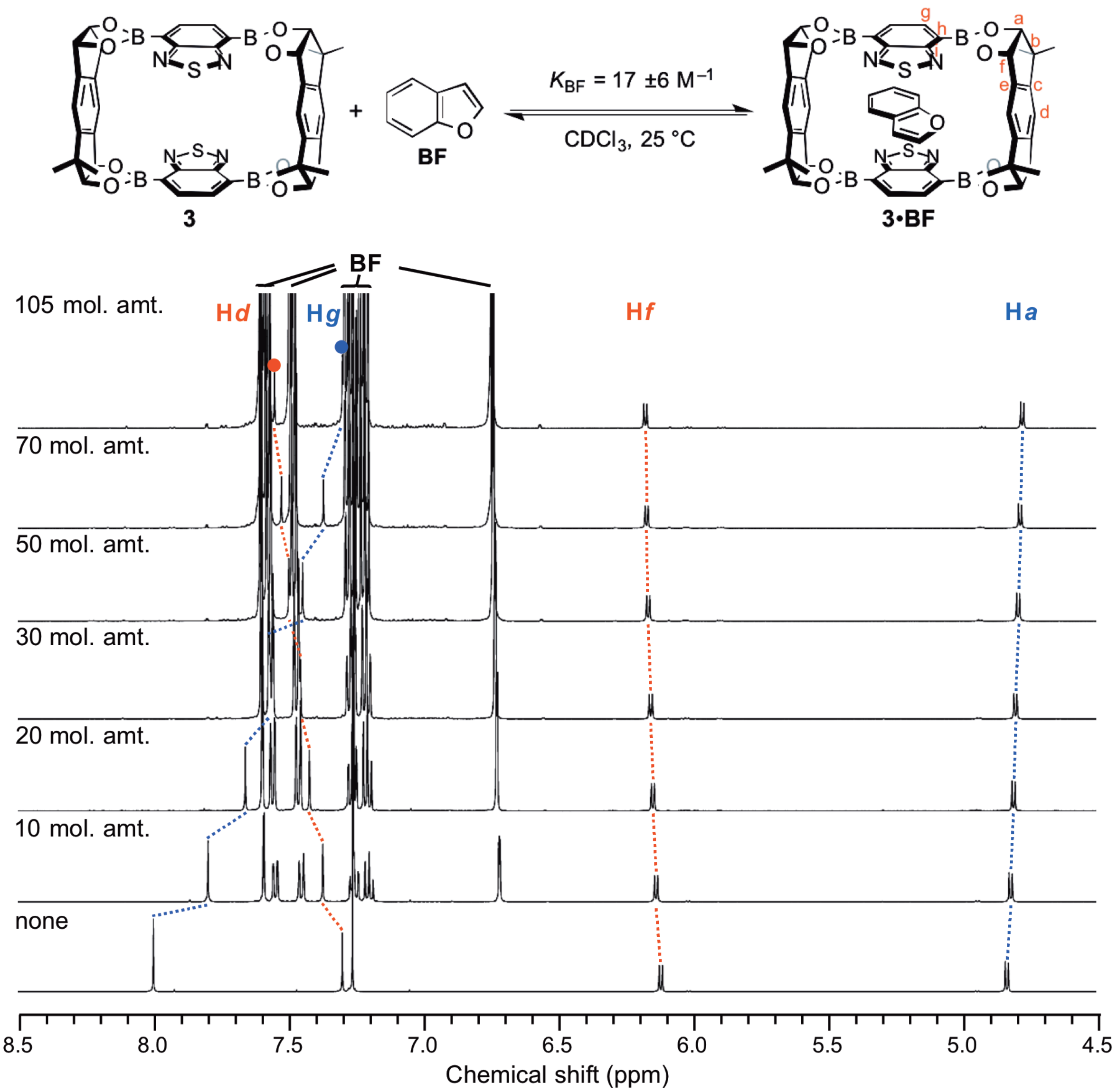

Figure S8. Partial ^{1}H NMR spectra of **3** with various amounts of benzofuran used for the determination of association constant K_{BF} (500 MHz, $CDCl_3$, 25 °C).

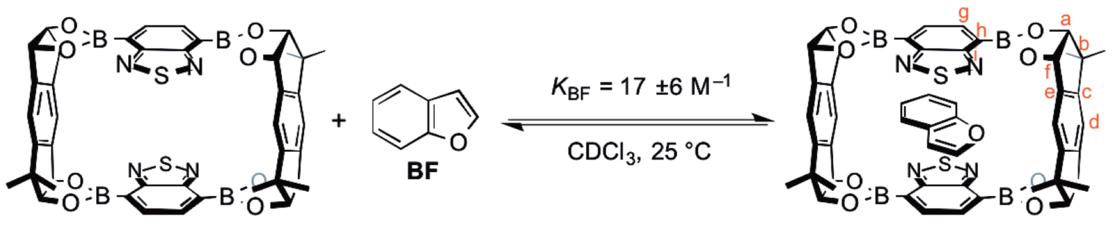

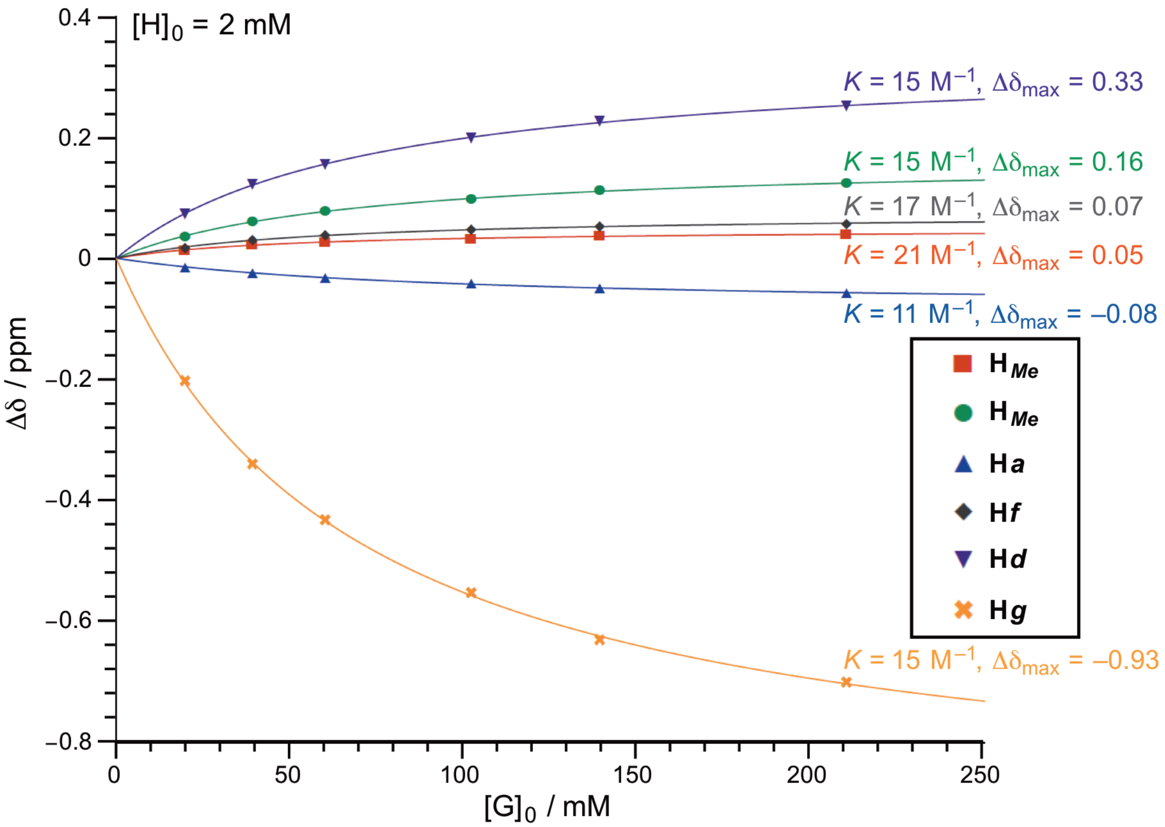

Figure S9. Changes in the chemical shift values Δδ of **3** plotted versus initial concentration of benzofuran $[G]_0$ and the corresponding fitting curve for the determination of association constant K_{BF}.

4) **3•QU**

Binding stoichiometry of **3** with **QU** was determined to be 1:1 by Job plot analysis of the ^{1}H NMR spectra of **3** with varying amount of **QU** in $CDCl_3$ with 0.03% TMS (v/v).

The NMR samples were prepared by the addition of different portions of **3** (2.0 mM) and **QU** (2.0 mM) so that the total concentration of added **3** and **QU** became 2.0 mM for each sample.

Table S10. Data table for Job plot of **3** with **QU** in $CDCl_3$

$[\mathbf{3}]_0$ / ($[\mathbf{3}]_0$+$[\mathbf{QU}]_0$)	**3** (μL)	**QU** (μL)	δ of H_g (ppm)	Δδ of H_g	Δδ ×$[\mathbf{3}]_0$ / ($[\mathbf{3}]_0$+$[\mathbf{QU}]_0$)
1.00	600	0	7.9980	0	0.00000
0.90	540	60	7.9962	0.0018	0.00162
0.80	480	120	7.9946	0.0034	0.00272
0.70	420	180	7.9926	0.0054	0.00378
0.68	410	190	7.9923	0.0057	0.00390
0.66	400	200	7.9919	0.0061	0.00407
0.64	385	215	7.9915	0.0065	0.00417
0.62	370	230	7.9911	0.0069	0.00426
0.60	360	240	7.9908	0.0072	0.00432
0.54	325	275	7.9898	0.0082	0.00444
0.52	310	290	7.9893	0.0087	0.004495
0.50	300	300	7.9890	0.0090	0.00450
0.48	290	310	7.9887	0.0093	0.004495
0.46	275	325	7.9883	0.0097	0.00445
0.40	240	360	7.9871	0.0109	0.00436
0.38	230	370	7.9868	0.0112	0.00429
0.36	215	385	7.9864	0.0116	0.00416
0.33	200	400	7.9860	0.0120	0.00400
0.32	190	410	7.9858	0.0122	0.00386
0.30	180	420	7.9855	0.0125	0.00375
0.20	120	480	7.9838	0.0142	0.00284
0.10	60	540	7.9820	0.0160	0.00160

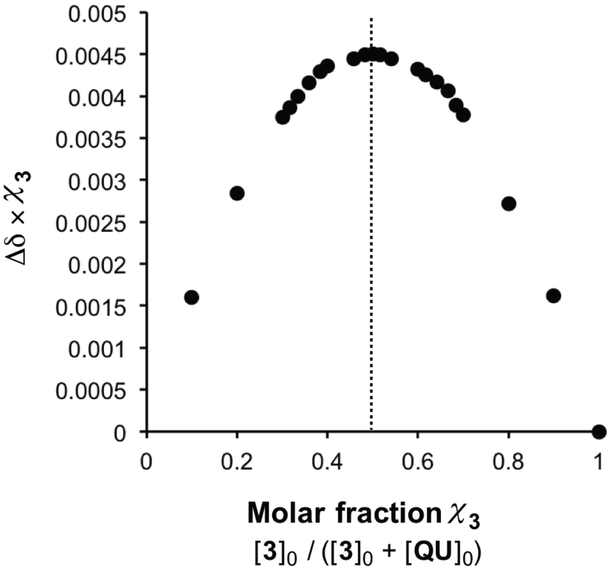

Figure S10. Job plot for NMR titration data of **3** and **QU**.

Table S11. Determination of association constant by the titration of **3** with **QU**

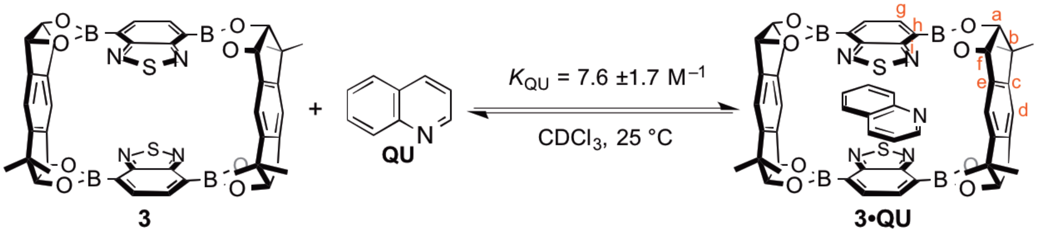

Entry	K_{QU} (M^{-1})
1st titration	7.2 ±1.5
2nd titration	7.9 ±1.5
3rd titration	7.7 ±1.8
Average	7.6 ±1.7

Table S12. Data tables for ^{1}H NMR titration of **3** with **QU**

1st titration

[G]$_0$ (M)	Δδ of H_{Me} 1.1801	Δδ of H_{Me} 1.3691	Δδ of H_a 4.8298	Δδ of H_f 6.1119	Δδ of H_d 7.2977	Δδ of H_g 7.998
0.021	0.0086	0.0269	-0.0103	0.0114	0.0498	-0.1495
0.044	0.0160	0.0492	-0.0189	0.0206	0.0922	-0.2743
0.066	0.0206	0.0658	-0.0263	0.0275	0.1225	-0.3699
0.124	0.0286	0.0922	-0.0366	0.0389	0.1712	-0.5136
0.166	0.0338	0.1105	-0.0466	0.0464	0.2044	-0.6127
0.225	0.0384	0.1277	-0.0526	0.0532	0.2323	-0.7077
K (M^{-1})	8.3	7.0	5.8	7.1	7.5	7.2
$\Delta\delta_{max}$	0.06	0.21	-0.09	0.09	0.37	-1.13
χ^2	1.28E-06	1.65E-05	6.49E-06	1.89E-06	3.81E-05	5.02E-04
R^2	0.9980	0.9978	0.9952	0.9986	0.9985	0.9978

[H]$_0$ = 2.0 mM

K_{QU} = 7.2 ±1.5 M^{-1}

2nd titration

$[G]_0$ (M)	Δδ of H_{Me} 1.1804	Δδ of H_{Me} 1.3694	Δδ of H_a 4.8282	Δδ of H_f 6.1106	Δδ of H_d 7.2979	Δδ of H_g 7.9984
0.021	0.0075	0.0241	-0.0097	0.0098	0.0458	-0.1380
0.042	0.0143	0.0447	-0.0171	0.0189	0.0825	-0.2492
0.067	0.0195	0.0619	-0.0240	0.0258	0.1129	-0.3477
0.117	0.0275	0.0882	-0.0343	0.0367	0.1633	-0.4897
0.173	0.0327	0.1066	-0.0423	0.0447	0.1982	-0.5917
0.280	0.0373	0.1260	-0.0515	0.0521	0.2221	-0.7011
K (M^{-1})	9.0	7.6	6.6	7.8	8.5	7.8
$\Delta\delta_{max}$	0.05	0.19	-0.08	0.08	0.32	-1.03
χ^2	1.48E-06	3.78E-06	1.02E-07	1.67E-06	9.49E-05	4.26E-05
R^2	0.9980	0.9995	0.9999	0.9988	0.9962	0.9998

$[H]_0$ = 2.0 mM

K_{QU} = 7.9 ±1.5 M^{-1}

3rd titration

$[G]_0$ (M)	Δδ of H_{Me} 1.1804	Δδ of H_{Me} 1.3689	Δδ of H_a 4.8282	Δδ of H_f 6.1100	Δδ of H_d 7.2979	Δδ of H_g 7.9978
0.022	0.0086	0.0263	-0.0103	0.0115	0.0481	-0.1455
0.044	0.0149	0.0469	-0.0177	0.0201	0.0831	-0.2560
0.068	0.0195	0.0630	-0.0246	0.0270	0.1163	-0.3488
0.117	0.0281	0.0899	-0.0343	0.0378	0.1655	-0.4948
0.182	0.0321	0.1076	-0.0435	0.0453	0.1999	-0.6019
0.266	0.0373	0.1265	-0.0521	0.0527	0.2221	-0.7022
K (M^{-1})	9.1	7.5	6.1	8.1	8.1	7.3
$\Delta\delta_{max}$	0.05	0.19	-0.08	0.08	0.33	-1.07
χ^2	1.64E-06	4.80E-06	8.46E-07	4.05E-07	6.10E-05	4.66E-05
R^2	0.9973	0.9993	0.9994	0.9997	0.9975	0.9998

$[H]_0$ = 2.0 mM

K_{QU} = 7.7 ±1.8 M^{-1}

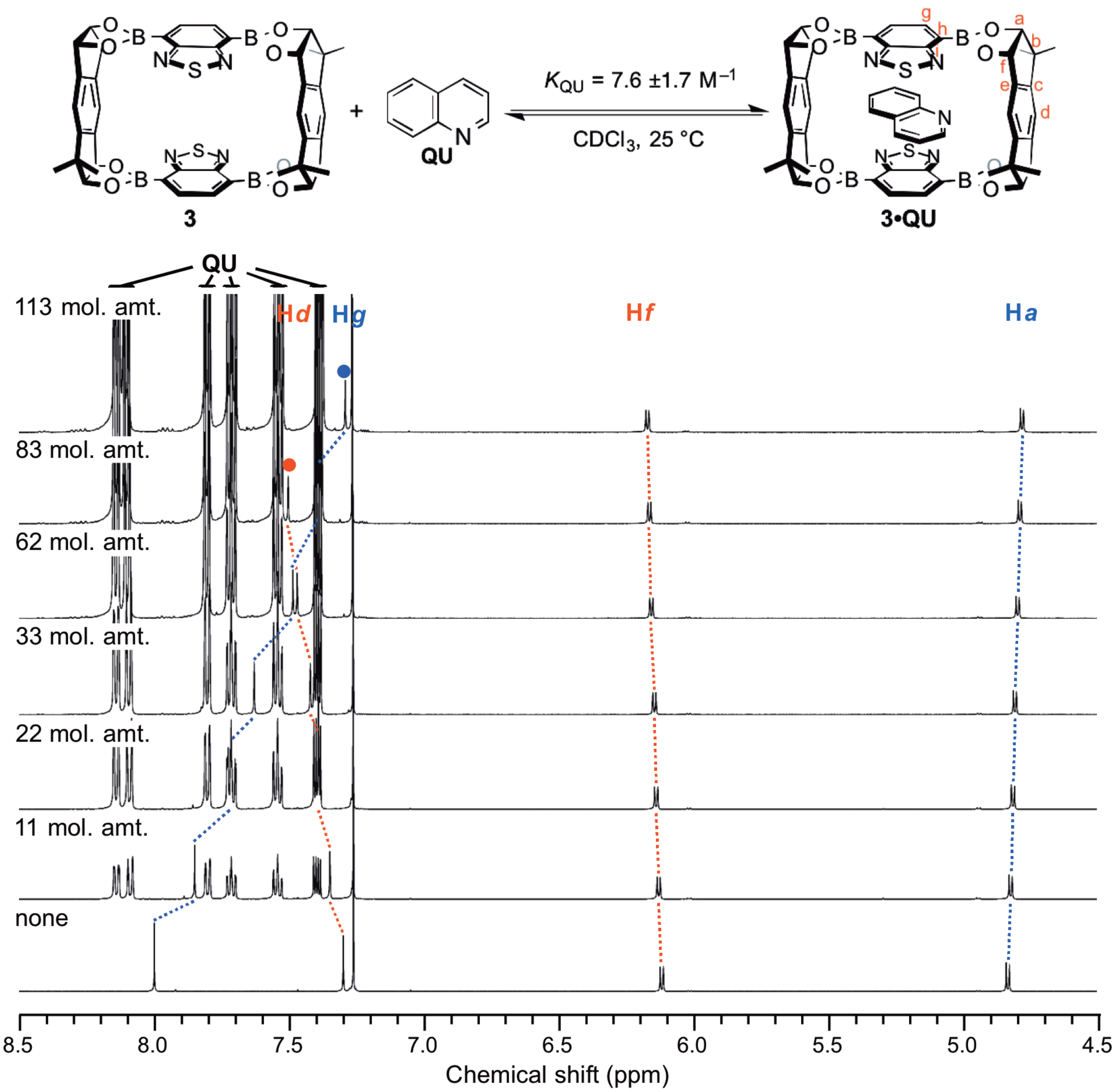

Figure S11. Partial ^{1}H NMR spectra of **3** with various amounts of quinoline used for the determination of association constant K_{QU} (500 MHz, $CDCl_3$, 25 °C).

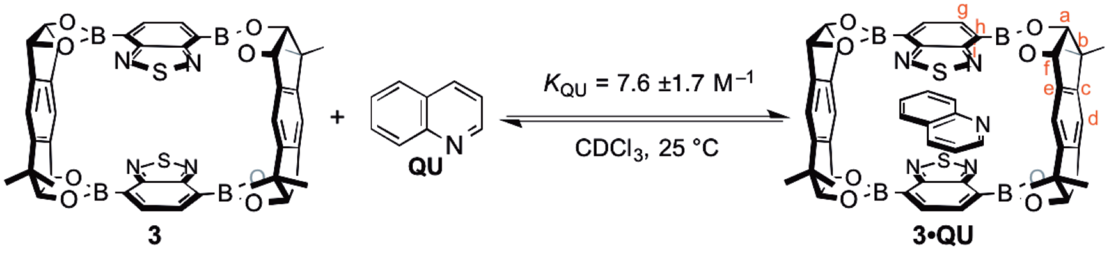

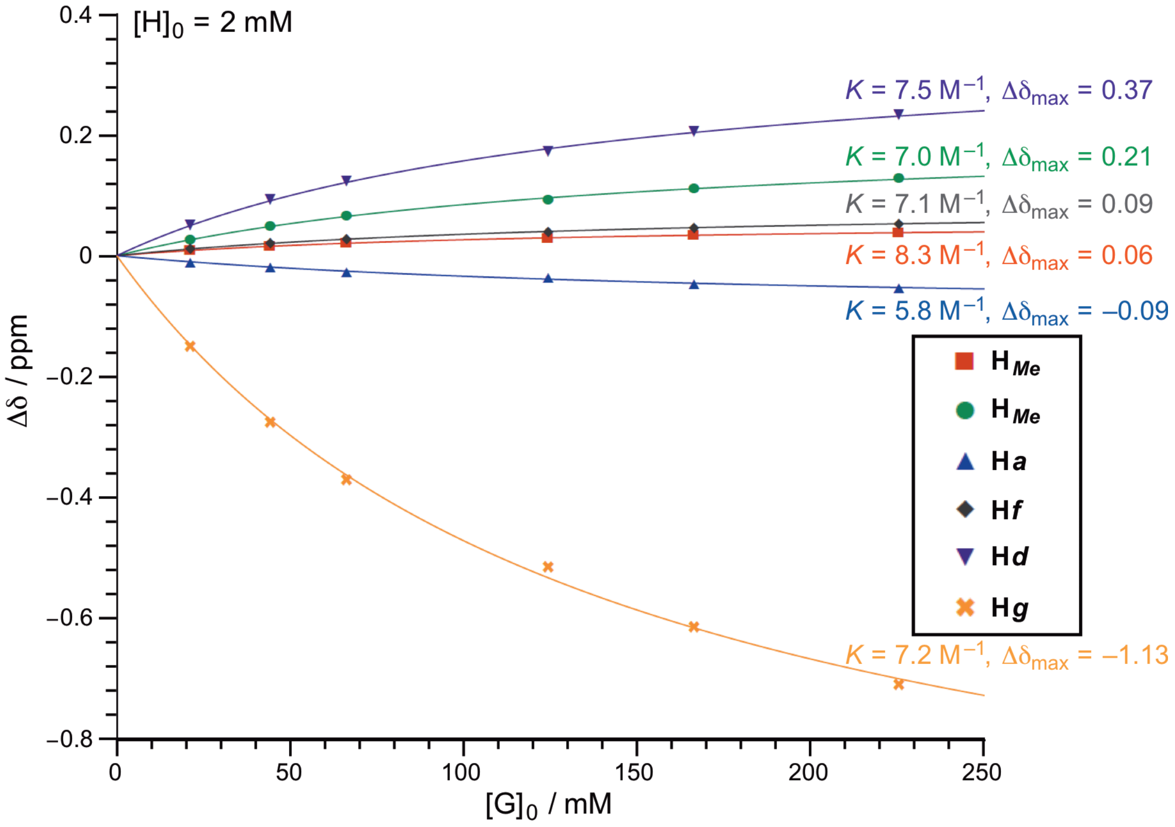

Figure S12. Changes in the chemical shift values Δδ of **3** plotted versus initial concentration of quinoline $[G]_0$ and the corresponding fitting curve for the determination of association constant K_{QU}.

5) **3•ANT**

Binding stoichiometry of **3** with **ANT** was determined to be 1:1 by Job plot analysis of the ^{1}H NMR spectra of **3** with varying amount of **ANT** in $CDCl_3$ with 0.03% TMS (v/v).

The NMR samples were prepared by the addition of different portions of **3** (2.0 mM) and **ANT** (2.0 mM) so that the total concentration of added **3** and **ANT** became 2.0 mM for each sample.

Table S13. Data table for Job plot of **3** with **ANT** in $CDCl_3$

[**3**]$_0$ / ([**3**]$_0$+[**ANT**]$_0$)	**3** (μL)	**ANT** (μL)	δ of H_g (ppm)	Δδ of H_g	Δδ ×[**3**]$_0$ / ([**3**]$_0$+[**ANT**]$_0$)
1.00	600	0	7.9980	0	0.00000
0.90	540	60	7.9492	0.0488	0.04392
0.80	480	120	7.9095	0.0885	0.07080
0.70	420	180	7.8557	0.1423	0.09961
0.68	410	190	7.8478	0.1502	0.10264
0.66	400	200	7.8402	0.1578	0.10520
0.64	385	215	7.8282	0.1698	0.10896
0.62	370	230	7.8162	0.1818	0.11211
0.60	360	240	7.8075	0.1905	0.11430
0.54	325	275	7.7816	0.2164	0.11722
0.52	310	290	7.7696	0.2284	0.11801
0.50	300	300	7.7633	0.2347	0.11735
0.48	290	310	7.7545	0.2435	0.11769
0.46	275	325	7.7444	0.2536	0.11623
0.40	240	360	7.7154	0.2826	0.11304
0.38	230	370	7.7084	0.2896	0.11101
0.36	215	385	7.6984	0.2996	0.10736
0.33	200	400	7.6870	0.3110	0.10367
0.32	190	410	7.6794	0.3186	0.10089
0.30	180	420	7.6719	0.3261	0.09783
0.20	120	480	7.6290	0.3690	0.07380
0.10	60	540	7.5881	0.4099	0.04099

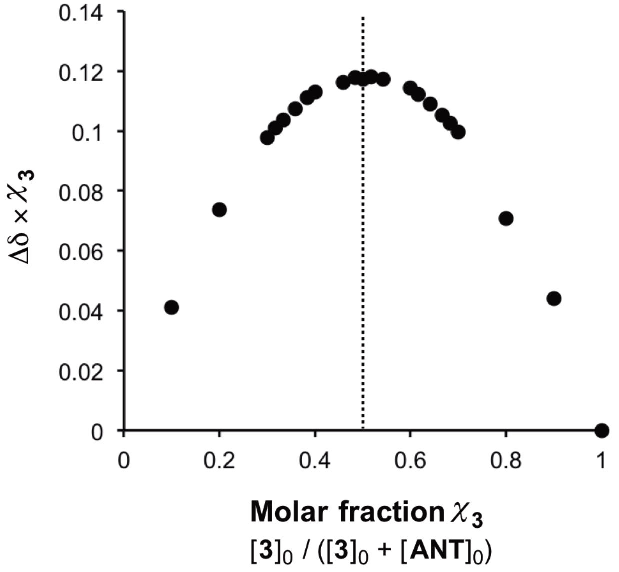

Figure S13. Job plot for NMR titration data of **3** and **ANT**.

Table S14. Determination of association constant by the titration of **3** with **ANT**

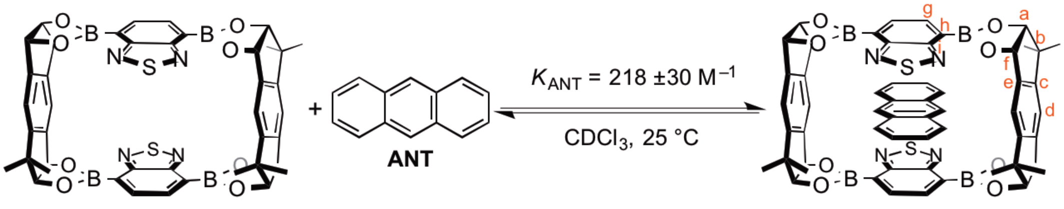

Entry	K_{ANT} (M^{-1})
1st titration	216.5 ±28.9
2nd titration	212.9 ±26.6
3rd titration	224.2 ±28.9
Average	218 ±30

Table S15. Data tables for ^{1}H NMR titration of **3** with **ANT**

1st titration

[G]$_0$ (M)	Δδ of H_{Me} 1.1799	Δδ of H_{Me} 1.3689	Δδ of H_a 4.8277	Δδ of H_f 6.1100	Δδ of H_d 7.2973	Δδ of H_g 7.9978
0.0030	0.0389	0.1031	-0.0235	0.0482	0.2051	-0.5395
0.0058	0.0567	0.1500	-0.0350	0.0699	0.2984	-0.7852
0.0089	0.0664	0.1764	-0.0413	0.0831	0.3517	-0.9255
0.0124	0.0715	0.1941	-0.0470	0.0905	0.3878	-1.0235
0.0181	0.0807	0.2170	-0.0516	0.1014	0.4308	-1.1386
0.0241	0.0841	0.2291	-0.0562	0.1066	0.4565	-1.2022
K (M^{-1})	236.4	218.5	186.9	221.3	218.7	217.0
$\Delta\delta_{max}$	0.10	0.27	-0.07	0.13	0.54	-1.43
χ^2	3.90E-06	1.26E-05	1.51E-06	2.60E-06	3.26E-05	1.93E-04
R^2	0.9972	0.9989	0.9979	0.9989	0.9993	0.9994

[H]$_0$ = 0.5 mM

K_{ANT} = 216.5 ±28.9 M^{-1}

2nd titration

[G]$_0$ (M)	Δδ of H_{Me} 1.1799	Δδ of H_{Me} 1.3689	Δδ of H_a 4.8277	Δδ of H_f 6.1100	Δδ of H_d 7.2973	Δδ of H_g 7.9984
0.0031	0.0389	0.1036	-0.0241	0.0482	0.2068	-0.5458
0.0059	0.0561	0.1494	-0.0344	0.0705	0.2973	-0.7830
0.0091	0.0664	0.1775	-0.0418	0.0831	0.3534	-0.9324
0.0126	0.0727	0.1958	-0.0464	0.0917	0.3895	-1.0264
0.0191	0.0807	0.2176	-0.0521	0.1014	0.4330	-1.1403
0.0259	0.0841	0.2291	-0.0562	0.1060	0.4560	-1.2011
K (M^{-1})	227.1	214.1	184.2	221.3	214.9	215.7
$Δδ_{max}$	0.10	0.27	-0.07	0.13	0.54	-1.42
$χ^2$	1.71E-06	5.49E-06	5.87E-07	3.59E-06	1.88E-05	1.17E-04
R^2	0.9988	0.9995	0.9992	0.9985	0.9996	0.9996

[H]$_0$ = 0.5 mM

K_{ANT} = 212.9 ±26.6 M^{-1}

3rd titration

[G]$_0$ (M)	Δδ of H_{Me} 1.1804	Δδ of H_{Me} 1.3689	Δδ of H_a 4.8282	Δδ of H_f 6.1100	Δδ of H_d 7.2979	Δδ of H_g 7.9984
0.0030	0.0390	0.1042	-0.0246	0.0487	0.2062	-0.5441
0.0058	0.0562	0.1500	-0.0349	0.0705	0.2973	-0.7835
0.0092	0.0659	0.1769	-0.0418	0.0831	0.3523	-0.9319
0.0120	0.0722	0.1953	-0.0469	0.0911	0.3878	-1.0241
0.0192	0.0791	0.2165	-0.0532	0.1009	0.4313	-1.1392
0.0260	0.0836	0.2291	-0.0561	0.1066	0.4565	-1.2056
K (M^{-1})	245.4	226.5	196.2	231.9	222.9	222.3
$Δδ_{max}$	0.10	0.27	-0.07	0.12	0.54	-1.42
$χ^2$	1.09E-06	6.86E-06	1.38E-06	1.10E-06	1.79E-05	8.62E-05
R^2	0.9992	0.9994	0.9981	0.9995	0.9996	0.9997

[H]$_0$ = 0.5 mM

K_{ANT} = 224.2 ±28.9 M^{-1}

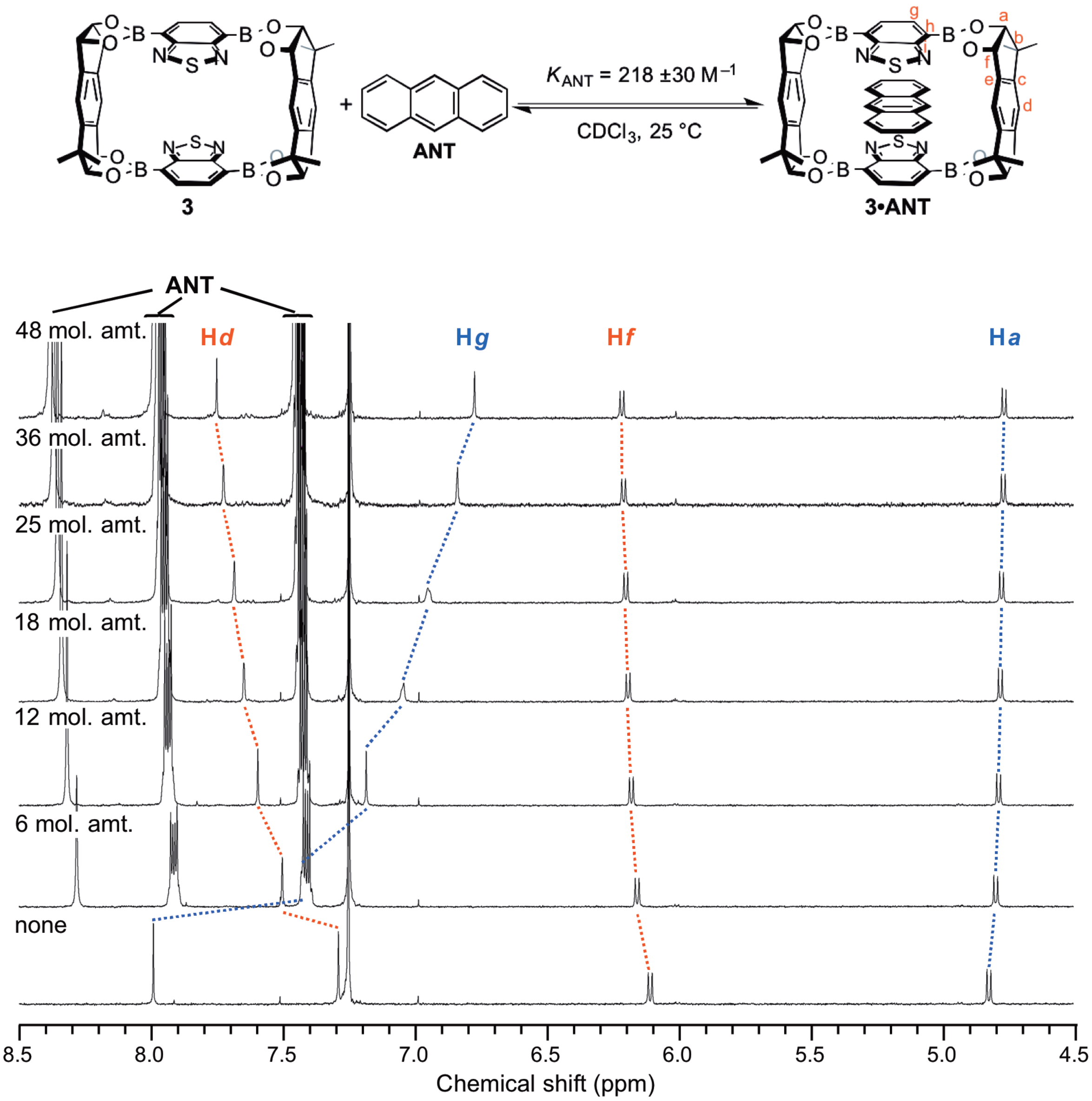

Figure S14. Partial ^{1}H NMR spectra of **3** with various amounts of anthracene used for the determination of association constant K_{ANT} (400 MHz, $CDCl_3$, 25 °C).

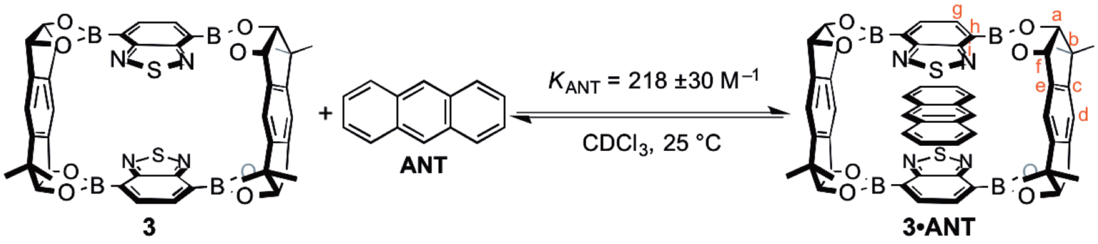

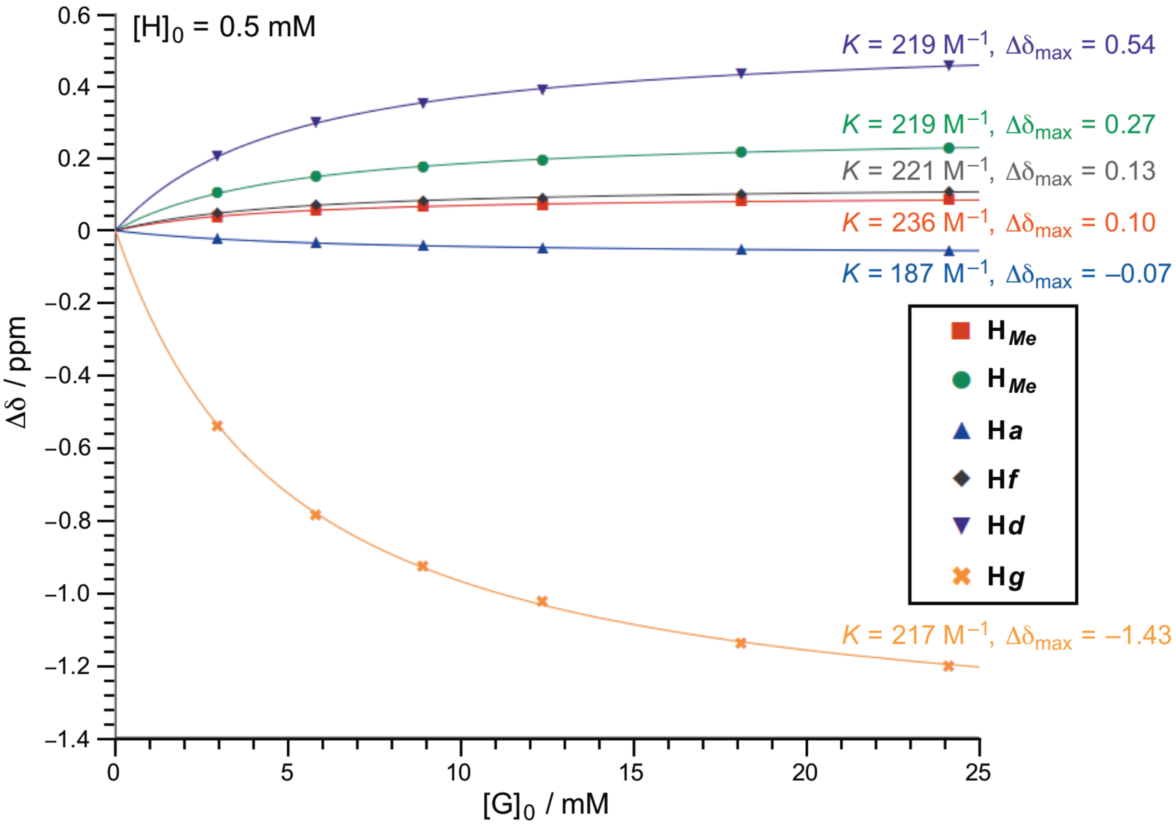

Figure S15. Changes in the chemical shift values Δδ of **3** plotted versus initial concentration of anthracene $[G]_0$ and the corresponding fitting curve for the determination of association constant K_{ANT}.

6) **3•DBT**

Binding stoichiometry of **3** with **DBT** was determined to be 1:1 by Job plot analysis of the ^{1}H NMR spectra of **3** with varying amount of **DBT** in $CDCl_3$ with 0.03% TMS (v/v).

The NMR samples were prepared by the addition of different portions of **3** (2.0 mM) and **DBT** (2.0 mM) so that the total concentration of added **3** and **DBT** became 2.0 mM for each sample.

Table S16. Data table for Job plot of **3** with **DBT** in $CDCl_3$

$[\mathbf{3}]_0$ / ($[\mathbf{3}]_0$+$[\mathbf{DBT}]_0$)	**3** (μL)	**DBT** (μL)	δ of H_g (ppm)	Δδ of H_g	Δδ ×$[\mathbf{3}]_0$ / ($[\mathbf{3}]_0$+$[\mathbf{DBT}]_0$)
1.00	600	0	7.9980	0	0.00000
0.90	540	60	7.9455	0.0525	0.04725
0.80	480	120	7.8957	0.1023	0.08184
0.70	420	180	7.8421	0.1559	0.10913
0.68	410	190	7.8339	0.1641	0.11214
0.66	400	200	7.8244	0.1736	0.11573
0.64	385	215	7.8112	0.1868	0.11986
0.62	370	230	7.7955	0.2025	0.12488
0.60	360	240	7.7880	0.2100	0.12600
0.54	325	275	7.7582	0.2398	0.12989
0.52	310	290	7.7444	0.2536	0.13103
0.50	300	300	7.7356	0.2624	0.13120
0.48	290	310	7.7265	0.2715	0.13123
0.46	275	325	7.7126	0.2854	0.13081
0.40	240	360	7.6815	0.3165	0.12660
0.38	230	370	7.6706	0.3274	0.12550
0.36	215	385	7.6643	0.3337	0.11958
0.33	200	400	7.6473	0.3507	0.11690
0.32	190	410	7.6397	0.3583	0.11346
0.30	180	420	7.6334	0.3646	0.10938
0.20	120	480	7.5836	0.4144	0.08288
0.10	60	540	7.5383	0.4597	0.04597

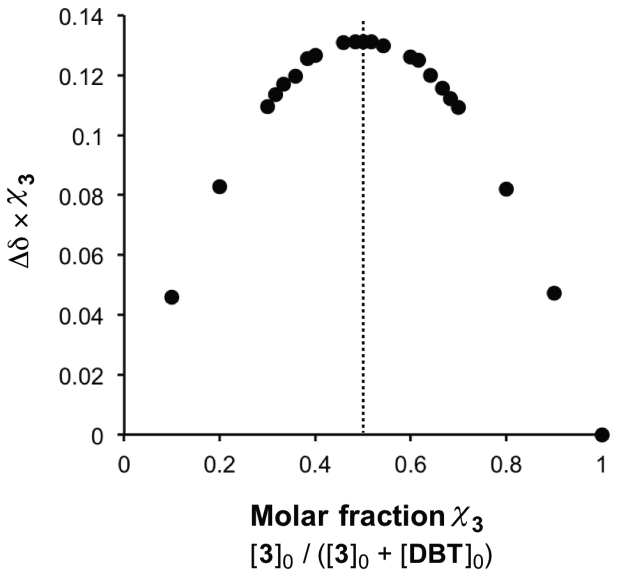

Figure S16. Job plot for NMR titration data of **3** and **DBT**.

Table S17. Determination of association constant by the titration of **3** with **DBT**

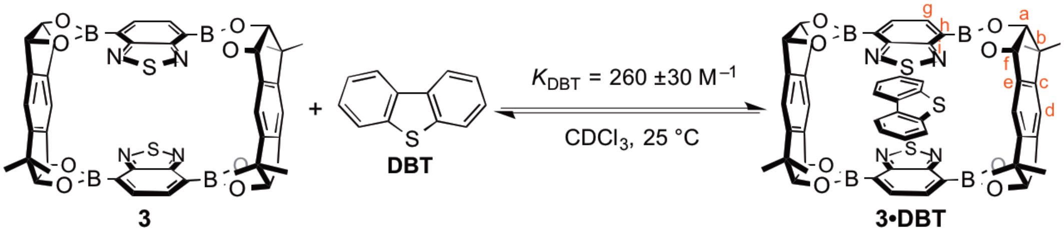

Entry	K_{DBT} (M^{-1})
1st titration	259.9 ±30.8
2nd titration	268.4 ±20.2
3rd titration	252.8 ±28.1
Average	260 ±30

Table S18. Data tables for ^{1}H NMR titration of **3** with **DBT**

1st titration

[G]$_0$ (M)	Δδ of H_{Me} 1.1799	Δδ of H_{Me} 1.3689	Δδ of H_a 4.8277	Δδ of H_f 6.1100	Δδ of H_d 7.2973	Δδ of H_g 7.9978
0.0031	0.0360	0.1002	-0.0298	0.0579	0.1931	-0.5853
0.0060	0.0509	0.1397	-0.0418	0.0814	0.2721	-0.8333
0.0087	0.0589	0.1678	-0.0487	0.0946	0.3162	-0.9691
0.0124	0.0630	0.1775	-0.0539	0.1020	0.3420	-1.0504
0.0177	0.0681	0.1941	-0.0590	0.1112	0.3735	-1.1455
0.0246	0.0715	0.2050	-0.0630	0.1175	0.3947	-1.2119
K (M^{-1})	286.1	260.1	232.7	265.6	260.3	254.7
Δδ_{max}	0.08	0.24	-0.07	0.14	0.46	-1.41
χ^2	2.25E-06	4.00E-05	5.64E-07	3.53E-06	3.46E-05	3.32E-04
R^2	0.9974	0.9946	0.9993	0.9985	0.9987	0.9987

[H]$_0$ = 0.5 mM

K_{DBT} = 259.9 ±30.8 M^{-1}

2nd titration

$[G]_0$ (M)	$\Delta\delta$ of H_{Me} 1.1799	$\Delta\delta$ of H_{Me} 1.3689	$\Delta\delta$ of H_a 4.8277	$\Delta\delta$ of H_f 6.1100	$\Delta\delta$ of H_d 7.2973	$\Delta\delta$ of H_g 7.9978
0.0030	0.0355	0.0996	-0.0304	0.0568	0.1919	-0.5908
0.0057	0.0498	0.1386	-0.0418	0.0808	0.2698	-0.8282
0.0091	0.0584	0.1661	-0.0493	0.0940	0.3156	-0.9691
0.0120	0.0630	0.1781	-0.0539	0.1020	0.3437	-1.0538
0.0183	0.0687	0.1941	-0.0590	0.1112	0.3735	-1.1463
0.0261	0.0717	0.2046	-0.0640	0.1175	0.3949	-1.2134
K (M^{-1})	282.3	269.4	247.7	270.5	270.0	270.7
$\Delta\delta_{max}$	0.08	0.23	-0.07	0.13	0.45	-1.39
χ^2	4.51E-07	2.97E-06	2.10E-06	1.63E-06	8.01E-06	6.15E-05
R^2	0.9995	0.9996	0.9973	0.9994	0.9997	0.9998

$[H]_0$ = 0.5 mM

K_{DBT} = 268.4 ±20.2 M^{-1}

3rd titration

$[G]_0$ (M)	$\Delta\delta$ of H_{Me} 1.1793	$\Delta\delta$ of H_{Me} 1.3683	$\Delta\delta$ of H_a 4.8277	$\Delta\delta$ of H_f 6.1095	$\Delta\delta$ of H_d 7.2968	$\Delta\delta$ of H_g 7.9978
0.0031	0.0366	0.1014	-0.0298	0.0584	0.1947	-0.5956
0.0061	0.0510	0.1415	-0.0418	0.0819	0.2720	-0.8322
0.0093	0.0595	0.1667	-0.0493	0.0951	0.3167	-0.9696
0.0123	0.0636	0.1787	-0.0539	0.1025	0.3436	-1.0550
0.0170	0.0687	0.1930	-0.0596	0.1105	0.3700	-1.1506
0.0219	0.0721	0.2027	-0.0619	0.1162	0.3894	-1.1942
K (M^{-1})	268.5	257.1	223.5	262.2	256.0	249.2
$\Delta\delta_{max}$	0.08	0.24	-0.07	0.14	0.46	-1.42
χ^2	4.08E-07	2.79E-06	1.58E-06	9.52E-07	1.46E-05	2.67E-04
R^2	0.9995	0.9996	0.9979	0.9996	0.9995	0.9990

$[H]_0$ = 0.5 mM

K_{DBT} = 252.8 ±28.1 M^{-1}

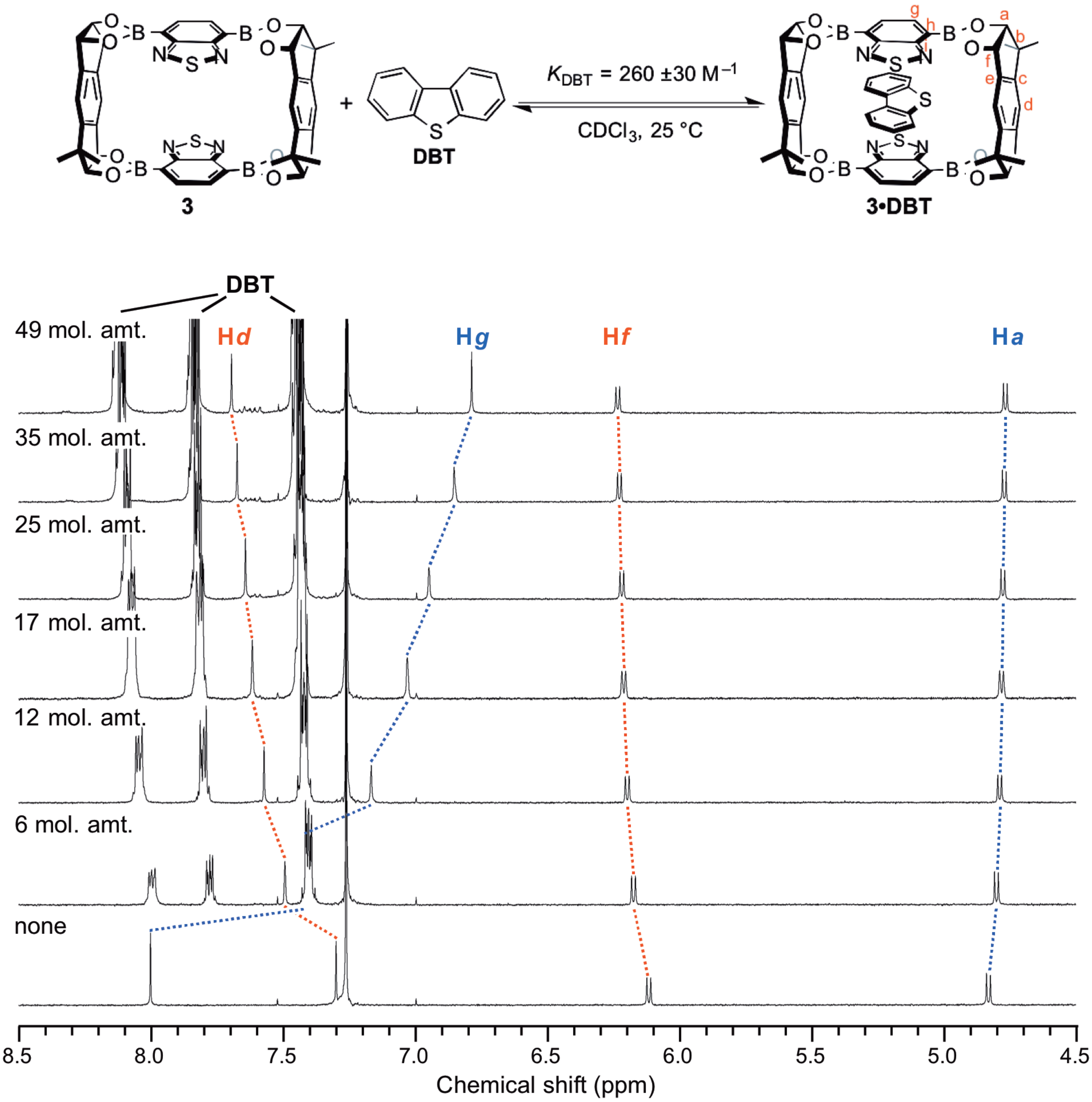

Figure S17. Partial ^{1}H NMR spectra of **3** with various amounts of dibenzothiophene used for the determination of association constant K_{DBT} (400 MHz, $CDCl_3$, 25 °C).

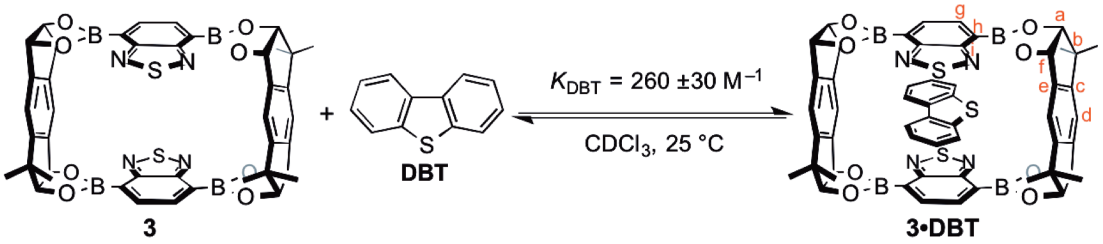

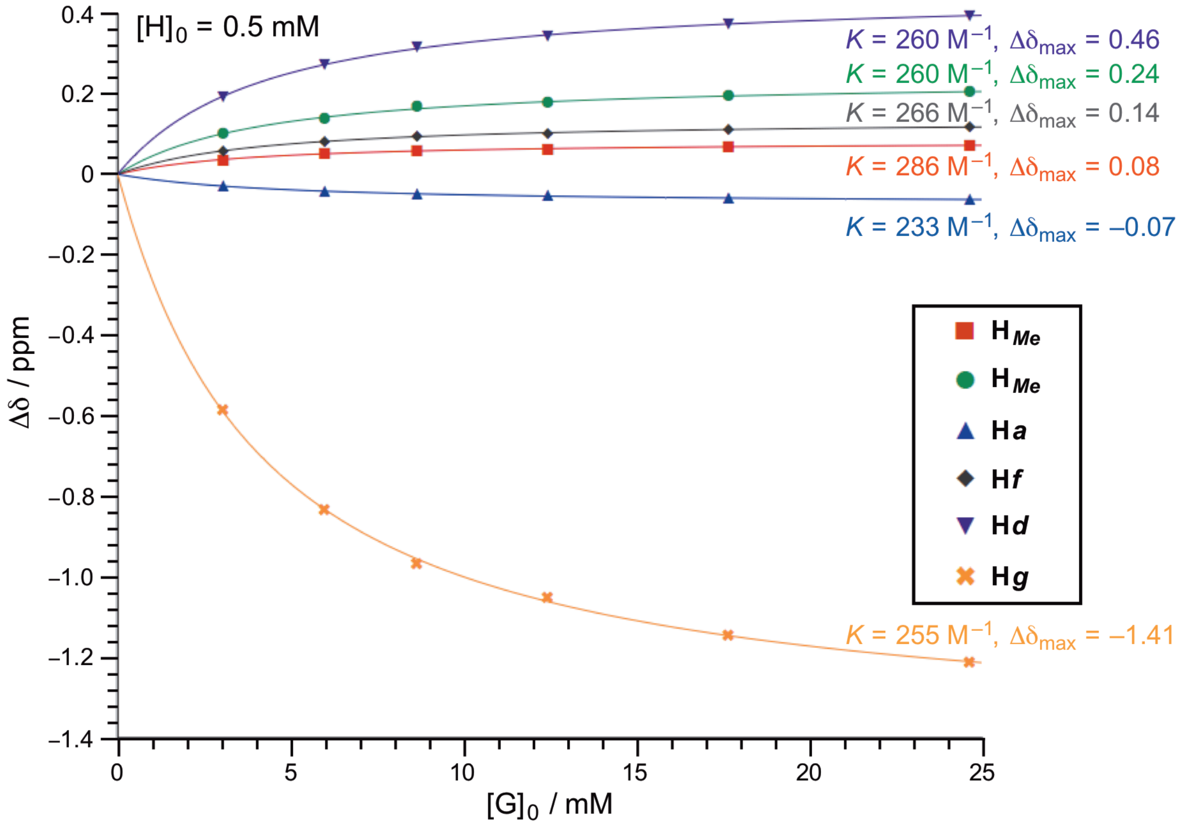

Figure S18. Changes in the chemical shift values Δδ of **3** plotted versus initial concentration of dibenzothiophene $[G]_0$ and the corresponding fitting curve for the determination of association constant K_{DBT}.

7) **3•DBF**

Binding stoichiometry of **3** with **DBF** was determined to be 1:1 by Job plot analysis of the ^{1}H NMR spectra of **3** with varying amount of **DBF** in $CDCl_3$ with 0.03% TMS (v/v).

The NMR samples were prepared by the addition of different portions of **3** (2.0 mM) and **DBF** (2.0 mM) so that the total concentration of added **3** and **DBF** became 2.0 mM for each sample.

Table S19. Data table for Job plot of **3** with **DBF** in $CDCl_3$

$[\mathbf{3}]_0$ / ($[\mathbf{3}]_0$+$[\mathbf{DBF}]_0$)	**3** (μL)	**DBF** (μL)	δ of H_g (ppm)	Δδ of H_g	Δδ ×$[\mathbf{3}]_0$ / ($[\mathbf{3}]_0$+$[\mathbf{DBF}]_0$)
1.00	600	0	7.9980	0	0.00000
0.90	540	60	7.9852	0.0128	0.01152
0.80	480	120	7.9732	0.0248	0.01984
0.70	420	180	7.9600	0.0380	0.02660
0.68	410	190	7.9581	0.0399	0.02727
0.66	400	200	7.9555	0.0425	0.02833
0.64	385	215	7.9526	0.0454	0.02913
0.62	370	230	7.9492	0.0488	0.03009
0.60	360	240	7.9473	0.0507	0.03042
0.54	325	275	7.9398	0.0582	0.03153
0.52	310	290	7.9373	0.0607	0.03136
0.50	300	300	7.9348	0.0632	0.03160
0.48	290	310	7.9328	0.0652	0.03151
0.46	275	325	7.9297	0.0683	0.03130
0.40	240	360	7.9228	0.0752	0.03008
0.38	230	370	7.9202	0.0778	0.02982
0.36	215	385	7.9177	0.0803	0.02877
0.33	200	400	7.9146	0.0834	0.02780
0.32	190	410	7.9127	0.0853	0.02701
0.30	180	420	7.9102	0.0878	0.02634
0.20	120	480	7.8982	0.0998	0.01996
0.10	60	540	7.8862	0.1118	0.01118

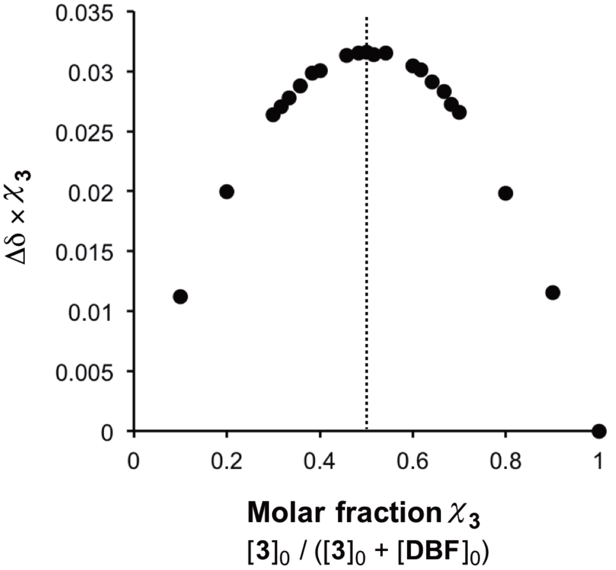

Figure S19. Job plot for NMR titration data of **3** and **DBF**.

Table S20. Determination of association constant by the titration of **3** with **DBF**

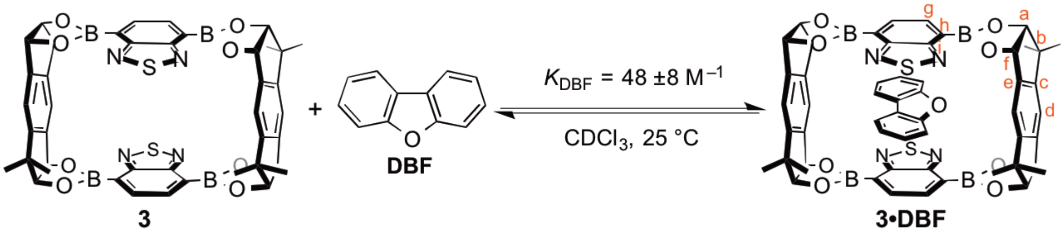

Entry	K_{DBF} (M^{-1})
1st titration	49.7 ±10.0
2nd titration	46.4 ±5.0
3rd titration	47.3 ±6.5
Average	48 ±8

Table S21. Data tables for ^{1}H NMR titration of **3** with **DBF**

1st titration

$[G]_0$ (M)	Δδ of H_{Me} 1.1799	Δδ of H_{Me} 1.3691	Δδ of H_a 4.8282	Δδ of H_f 6.1100	Δδ of H_d 7.2973	Δδ of H_g 7.9984
0.0054	0.0148	0.0450	-0.0148	0.0212	0.0848	-0.2560
0.0102	0.0240	0.0748	-0.0240	0.0350	0.1398	-0.4210
0.0208	0.0355	0.1132	-0.0372	0.0539	0.2137	-0.6454
0.0300	0.0423	0.1349	-0.0446	0.631	0.2627	-0.7709
0.0470	0.0481	0.1559	-0.0532	0.0728	0.2950	-0.8918
0.0649	0.0509	0.1699	-0.0595	0.0785	0.3225	-0.9765
K (M^{-1})	58.1	49.7	41.0	52.2	49.2	48.1
$Δδ_{max}$	0.07	0.22	-0.08	0.10	0.43	-1.29
$χ^2$	1.84E-06	6.18E-06	3.11E-07	2.94E-06	1.12E-04	1.75E-04
R^2	0.9983	0.9995	0.9998	0.9990	0.9975	0.9996

$[H]_0$ = 0.5 mM

K_{DBF} = 49.7 ±10.0 M^{-1}

2nd titration

[G]$_0$ (M)	Δδ of H_{Me} 1.1799	Δδ of H_{Me} 1.3691	Δδ of H_a 4.8282	Δδ of H_f 6.1100	Δδ of H_d 7.2973	Δδ of H_g 7.9984
0.0052	0.0148	0.0462	-0.0143	0.0218	0.0865	-0.2606
0.0108	0.0229	0.0736	-0.0246	0.0344	0.1392	-0.4198
0.0214	0.0360	0.1137	-0.0372	0.0539	0.2137	-0.6454
0.0313	0.0423	0.1355	-0.0452	0.0642	0.2627	-0.7732
0.0446	0.0481	0.1521	-0.0504	0.0716	0.2827	-0.8631
0.0622	0.0521	0.1704	-0.0584	0.0797	0.3208	-0.9691
K (M^{-1})	49.5	46.4	41.1	47.3	47.4	46.4
$\Delta\delta_{max}$	0.07	0.23	-0.08	0.11	0.43	-1.30
χ^2	2.22E-06	1.33E-05	2.72E-06	4.05E-06	1.46E-04	4.13E-04
R^2	0.9979	0.9989	0.9980	0.9984	0.9964	0.9989

[H]$_0$ = 0.5 mM

K_{DBF} = 46.4 ±5.0 M^{-1}

3rd titration

[G]$_0$ (M)	Δδ of H_{Me} 1.1799	Δδ of H_{Me} 1.3691	Δδ of H_a 4.8282	Δδ of H_f 6.1100	Δδ of H_d 7.2973	Δδ of H_g 7.9984
0.0054	0.0143	0.0444	-0.0148	0.0212	0.0842	-0.2549
0.0104	0.0240	0.0742	-0.0246	0.0350	0.1398	-0.4210
0.0217	0.0355	0.1132	-0.0378	0.0533	0.2131	-0.6454
0.0313	0.0423	0.1338	-0.0446	0.0636	0.2627	-0.7738
0.0442	0.0481	0.1549	-0.0526	0.0734	0.2967	-0.8843
0.0650	0.0515	0.1693	-0.0589	0.0791	0.3208	-0.9685
K (M^{-1})	53.2	47.0	41.8	48.2	46.7	46.8
$\Delta\delta_{max}$	0.07	0.23	-0.08	0.11	0.43	-1.30
χ^2	2.01E-06	8.79E-06	1.36E-06	3.80E-06	1.07E-04	2.35E-04
R^2	0.9981	0.9992	0.9991	0.9985	0.9976	0.9994

[H]$_0$ = 0.5 mM

K_{DBF} = 47.3 ±6.5 M^{-1}

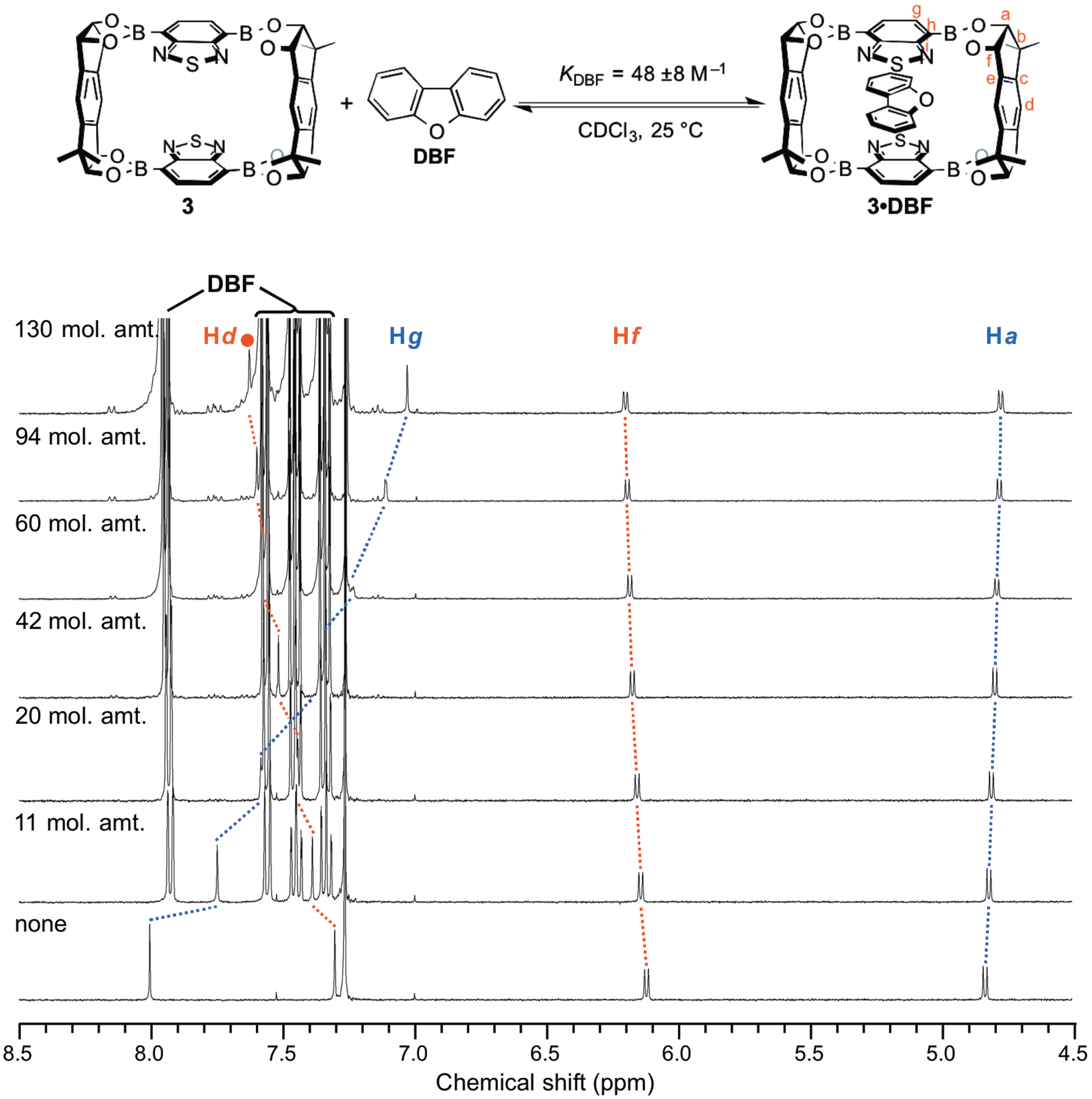

Figure S20. Partial ^{1}H NMR spectra of **3** with various amounts of dibenzofuran used for the determination of association constant K_{DBF} (400 MHz, $CDCl_3$, 25 °C).

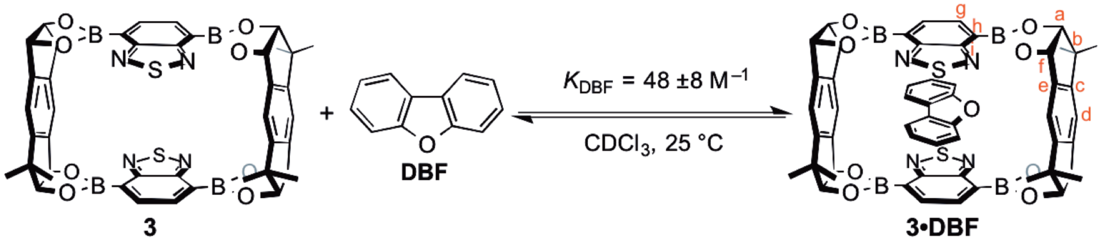

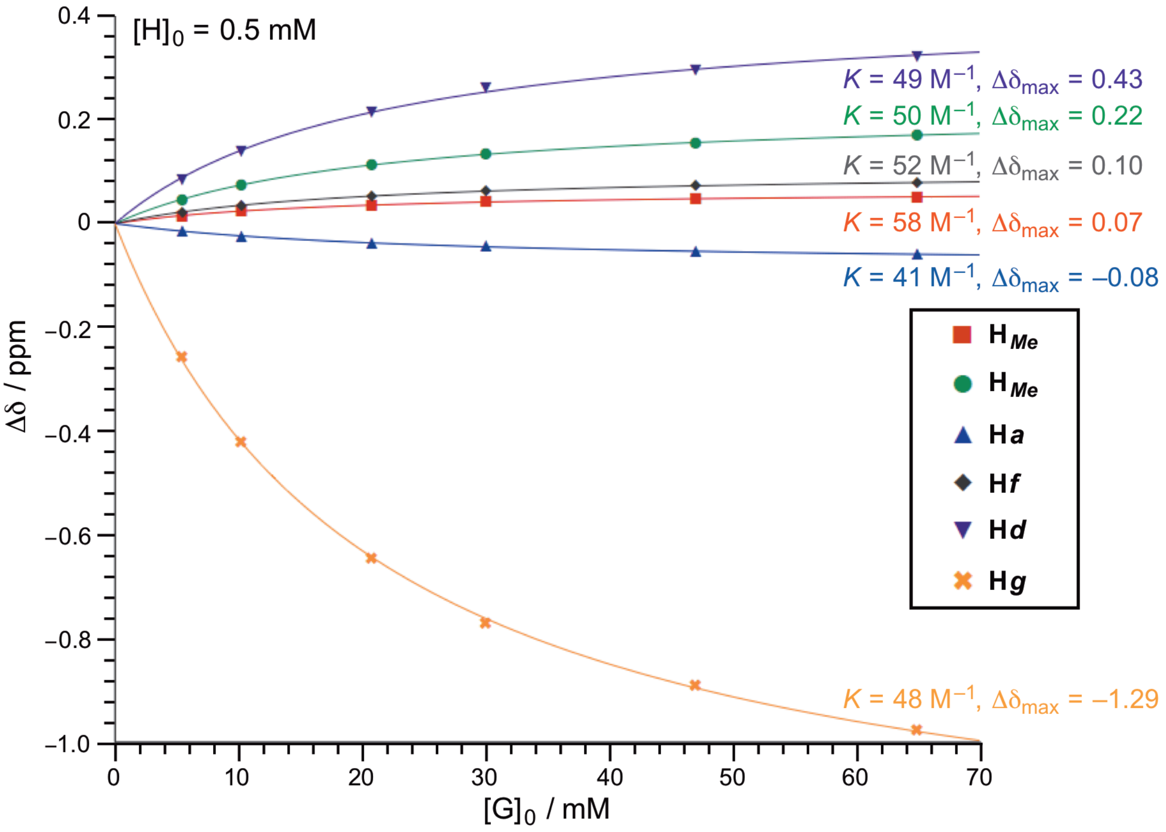

Figure S21. Changes in the chemical shift values Δδ of **3** plotted versus initial concentration of dibenzofuran $[G]_0$ and the corresponding fitting curve for the determination of association constant K_{DBF}.

8) **3•ACR**

Binding stoichiometry of **3** with **ACR** was determined to be 1:1 by Job plot analysis of the ^{1}H NMR spectra of **3** with varying amount of **ACR** in $CDCl_3$ with 0.03% TMS (v/v).

The NMR samples were prepared by the addition of different portions of **3** (2.0 mM) and **ACR** (2.0 mM) so that the total concentration of added **3** and **ACR** became 2.0 mM for each sample.

Table S22. Data table for Job plot of **3** with **ACR** in $CDCl_3$

$[\mathbf{3}]_0$ / ($[\mathbf{3}]_0$+$[\mathbf{ACR}]_0$)	**3** (μL)	**ACR** (μL)	δ of H_g (ppm)	Δδ of H_g	Δδ ×$[\mathbf{3}]_0$ / ($[\mathbf{3}]_0$+$[\mathbf{ACR}]_0$)
1.00	600	0	7.9980	0	0.00000
0.90	540	60	7.9946	0.0034	0.00306
0.80	480	120	7.9915	0.0065	0.00520
0.70	420	180	7.9877	0.0103	0.00721
0.68	410	190	7.9871	0.0109	0.00745
0.66	400	200	7.9864	0.0116	0.00773
0.64	385	215	7.9855	0.0125	0.00802
0.62	370	230	7.9845	0.0135	0.00833
0.60	360	240	7.9839	0.0141	0.00846
0.54	325	275	7.9818	0.0162	0.00878
0.52	310	290	7.9808	0.0172	0.00889
0.50	300	300	7.9801	0.0179	0.00895
0.48	290	310	7.9795	0.0185	0.00894
0.46	275	325	7.9788	0.0192	0.00880
0.40	240	360	7.9768	0.0212	0.00848
0.38	230	370	7.9763	0.0217	0.00832
0.36	215	385	7.9753	0.0227	0.00813
0.33	200	400	7.9744	0.0236	0.00787
0.32	190	410	7.9738	0.0242	0.00766
0.30	180	420	7.9736	0.0244	0.00732
0.20	120	480	7.9707	0.0273	0.00546
0.10	60	540	7.9669	0.0311	0.00311

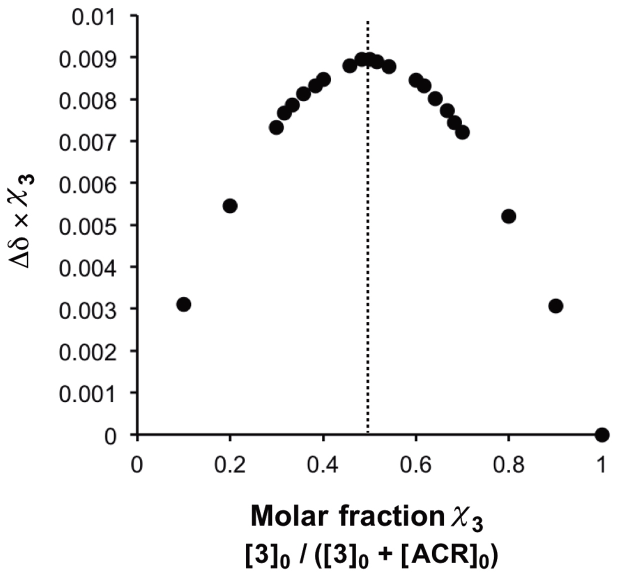

Figure S22. Job plot for NMR titration data of **3** and **ACR**.

Table S23. Determination of association constant by the titration of **3** with **ACR**

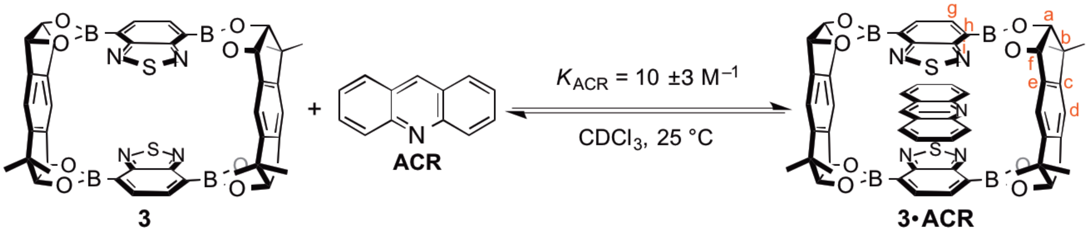

Entry	K_{ACR} (M^{-1})
1st titration	8.7 ±1.4
2nd titration	10.9 ±1.4
3rd titration	11.0 ±3.2
Average	10 ±3

Table S24. Data tables for ^{1}H NMR titration of **3** with **ACR**

1st titration

[G]$_0$ (M)	Δδ of H_{Me} 1.1801	Δδ of H_{Me} 1.3691	Δδ of H_a 4.8298	Δδ of H_f 6.1119	Δδ of H_d 7.2977	Δδ of H_g 7.998
0.006	0.0046	0.0137	-0.0051	0.0057	0.0281	-0.0807
0.012	0.0092	0.0275	-0.0097	0.0114	0.0538	-0.154
0.020	0.0138	0.0401	-0.0131	0.0171	0.0779	-0.2193
0.032	0.0201	0.0601	-0.02	0.0257	0.1168	-0.331
0.045	0.0264	0.0773	-0.0257	0.0332	0.1512	-0.426
0.061	0.0321	0.0973	-0.0338	0.0412	0.1907	-0.54
K (M^{-1})	9.9	8.8	7.4	8.8	8.6	8.7
$\Delta\delta_{max}$	0.09	0.28	-0.11	0.12	0.55	-1.55
χ^2	1.61E-07	2.02E-06	2.42E-06	8.55E-08	1.16E-05	1.56E-04
R^2	0.9997	0.9996	0.9964	0.9999	0.9995	0.9991

[H]$_0$ = 0.5 mM

K_{ACR} = 8.7 ±1.4 M^{-1}

2nd titration

$[G]_0$ (M)	Δδ of H_{Me} 1.1799	Δδ of H_{Me} 1.3689	Δδ of H_a 4.8277	Δδ of H_f 6.1100	Δδ of H_d 7.2973	Δδ of H_g 7.9978
0.005	0.0045	0.0120	-0.0035	0.0052	0.0235	-0.0658
0.011	0.0085	0.0252	-0.0080	0.0109	0.0487	-0.1369
0.023	0.0166	0.0481	-0.0155	0.0207	0.0946	-0.2652
0.033	0.0229	0.0658	-0.0212	0.0281	0.1272	-0.3688
0.052	0.0309	0.0899	-0.0281	0.0384	0.1765	-0.4878
0.071	0.0360	0.1071	-0.0350	0.0464	0.2074	-0.5905
K (M^{-1})	12.08	10.72	9.86	10.34	11.19	10.95
$\Delta\delta_{max}$	0.08	0.25	-0.08	0.11	0.48	-1.35
χ^2	1.37E-06	3.47E-06	8.39E-07	3.42E-07	1.56E-05	2.44E-04
R^2	0.9982	0.9995	0.9988	0.9997	0.9994	0.9988

$[H]_0$ = 0.5 mM

K_{ACR} = 10.9 ±1.4 M^{-1}

3rd titration

$[G]_0$ (M)	Δδ of H_{Me} 1.1799	Δδ of H_{Me} 1.3689	Δδ of H_a 4.8277	Δδ of H_f 6.1100	Δδ of H_d 7.2973	Δδ of H_g 7.9978
0.006	0.0057	0.0143	-0.0035	0.0063	0.0275	-0.0750
0.011	0.0091	0.0252	-0.0075	0.0104	0.0493	-0.1380
0.024	0.0171	0.0492	-0.0155	0.0212	0.0951	-0.2680
0.035	0.0223	0.0647	-0.0201	0.0281	0.1243	-0.3390
0.047	0.0297	0.0887	-0.0275	0.0378	0.1627	-0.4678
0.075	0.0360	0.1071	-0.0355	0.0459	0.2085	-0.5848
K (M^{-1})	13.53	11.45	8.09	11.52	10.81	10.51
$\Delta\delta_{max}$	0.07	0.24	-0.10	0.10	0.47	-1.34
χ^2	3.61E-06	4.45E-05	2.52E-06	6.82E-06	3.27E-05	9.27E-04
R^2	0.9948	0.9931	0.9967	0.9943	0.9986	0.9951

$[H]_0$ = 0.5 mM

K_{ACR} = 11.0 ±3.2 M^{-1}

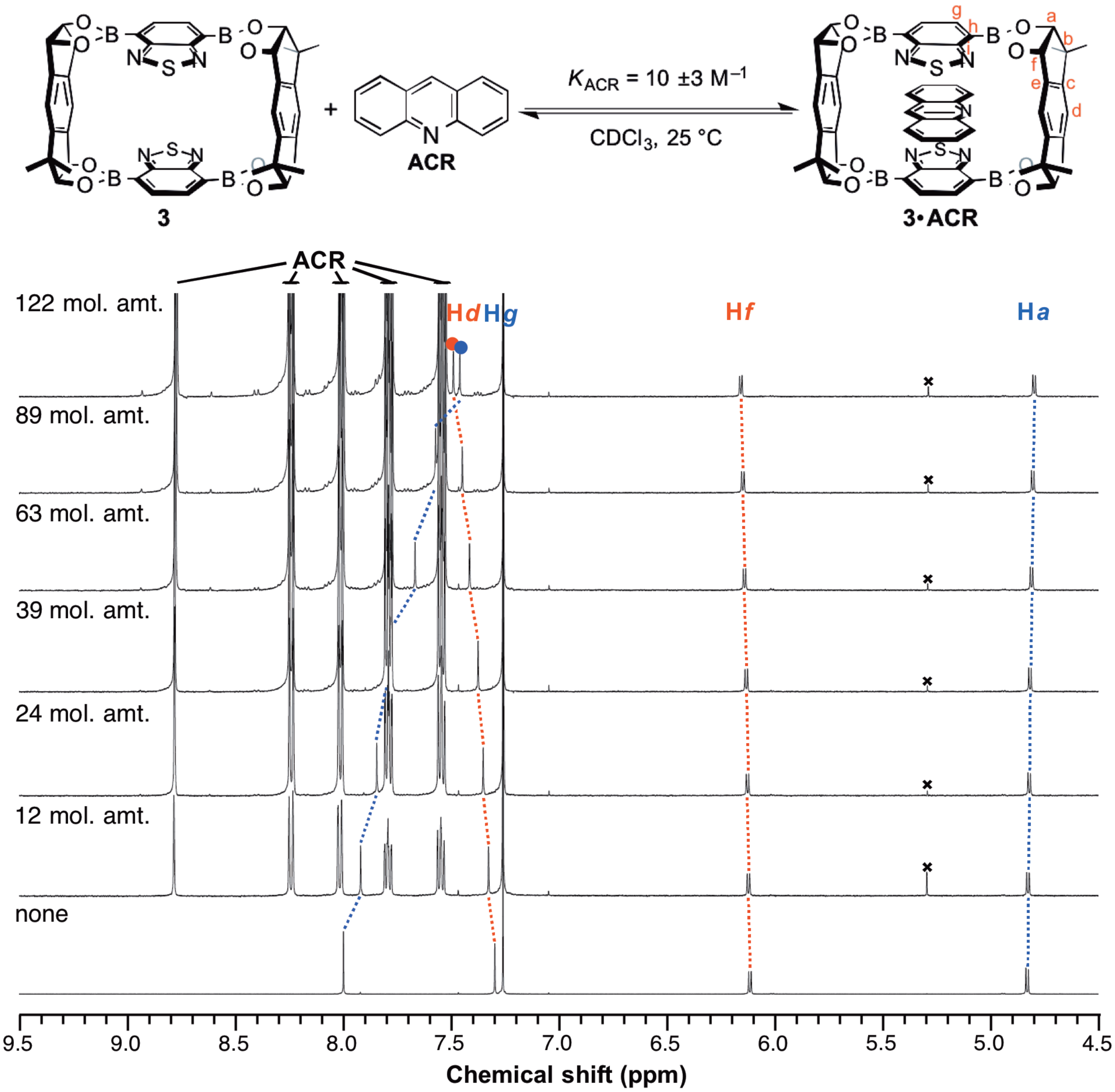

Figure S23. Partial ^{1}H NMR spectra of **3** with various amounts of acridine used for the determination of association constant K_{ACR} (500 MHz, $CDCl_3$, 25 °C).

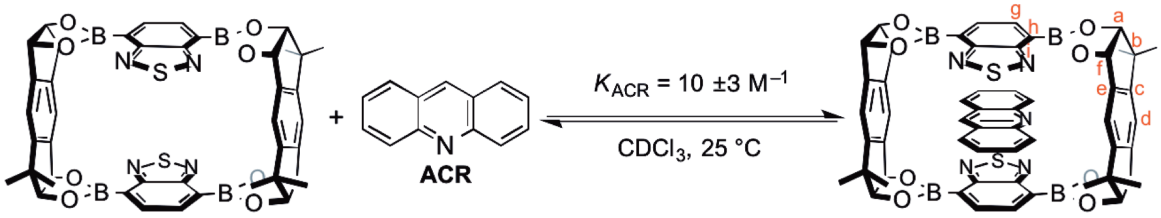

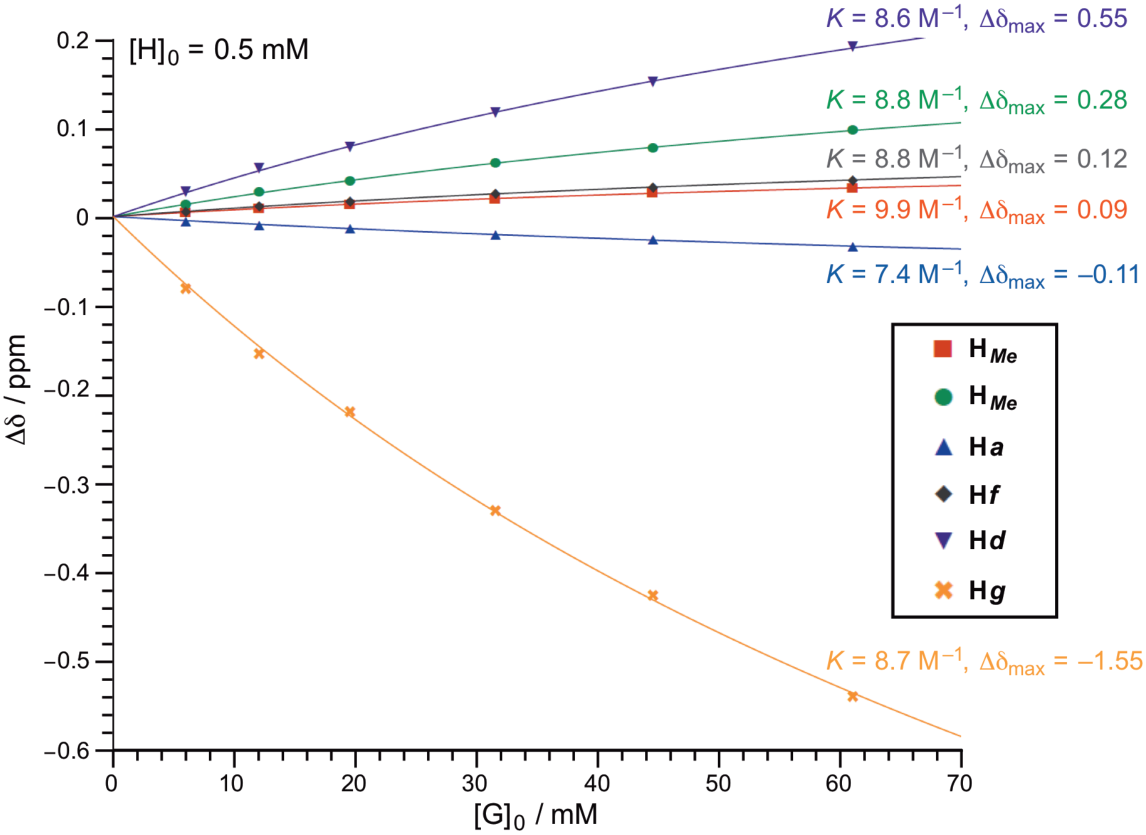

Figure S24. Changes in the chemical shift values Δδ of **3** plotted versus initial concentration of acridine $[G]_0$ and the corresponding fitting curve for the determination of association constant K_{ACR}.

4. Powder X-ray Diffraction Analysis

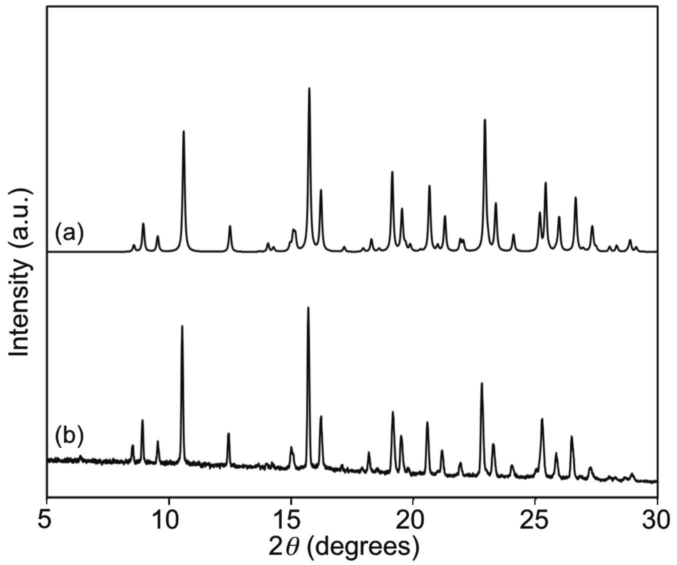

Figure S25. PXRD patterns of **3•ANT•**CH_2Cl_2. (a) Simulated PXRD pattern calculated from the single-crystal structure. (b) Experimental PXRD patterns of the precipitate.

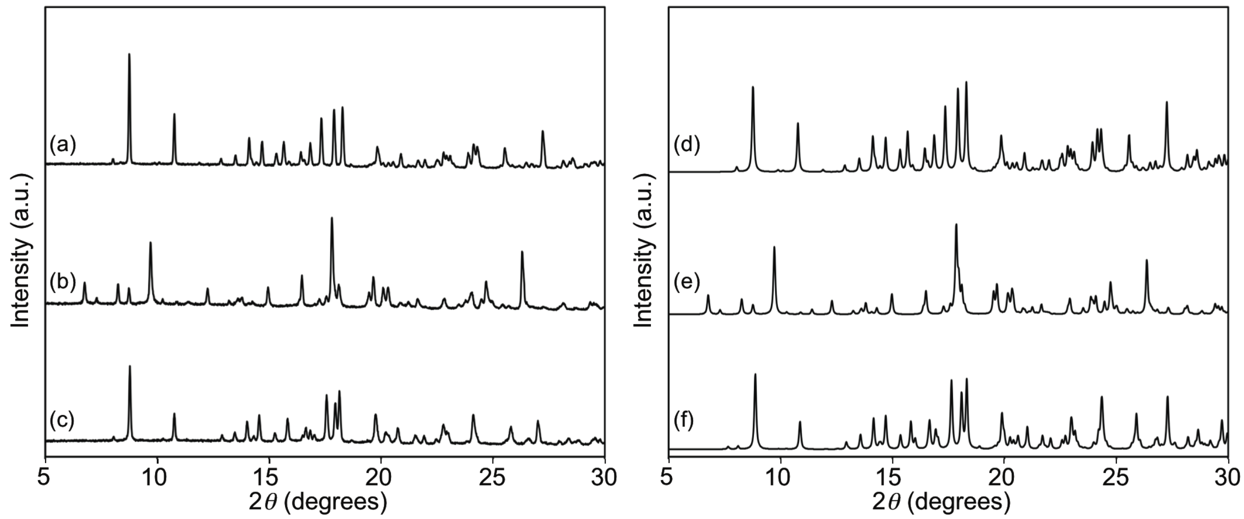

Figure S26. (a)~(c) Experimental PXRD patterns of the precipitate of (a) **3•NA•**$4CHCl_3$, (b) **3•ANT•**$4CHCl_3$, (c) **3•**$\mathbf{CHCl_3}$**•**$4CHCl_3$. (d)~(f) Simulated PXRD patterns calculated from the single-crystal structure of (d) **3•NA•**$4CHCl_3$, (e) **3•ANT•**$4CHCl_3$, (f) **3•**$\mathbf{CHCl_3}$**•**$4CHCl_3$.

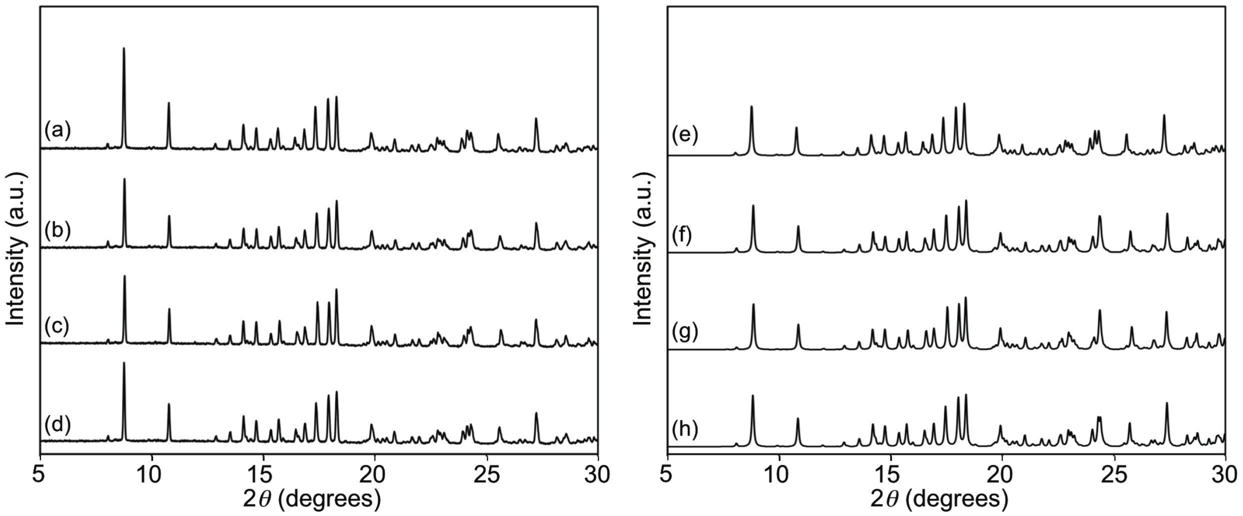

Figure S27. (a)~(d) Experimental PXRD patterns of the precipitate of (a) **3•NA**•4$CHCl_3$, (b) **3•BT**•4$CHCl_3$, (c) **3•BF**•4$CHCl_3$, (d) **3•QU**•4$CHCl_3$. (e)~(h) Simulated PXRD pattern calculated from the single-crystal structure of (e) **3•NA**•4$CHCl_3$, (f) **3•BT**•4$CHCl_3$, (g) **3•BF**•4$CHCl_3$, (h) **3•QU**•4$CHCl_3$.

5. X-ray Crystallographic Analysis

1) **3•ANT**•CH_2Cl_2

The single crystal of **3•ANT**•CH_2Cl_2 suitable for X-ray crystallographic analysis was obtained by vapor diffusion of hexane into the dichloromethane solution of **3•ANT**. Each unit cell of the crystal consisted of four **3•ANT** complexes and four dichloromethane molecules.

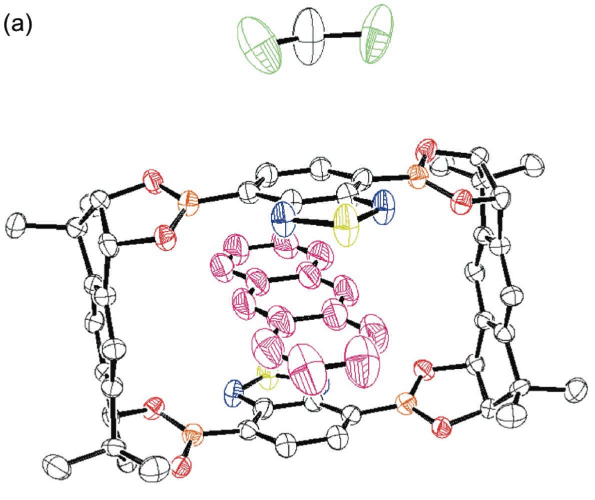

Figure S28. X-ray crystal structure of 3•ANT•CH_2Cl_2 (CCDC 1005494). All hydrogen atoms are omitted for clarity. (a) ORTEP structure with thermal ellipsoids shown at 50% probability (C = gray, O = red, B = orange, N = blue, S = yellow, Cl = green, anthracene = pink). (b) Schematic representation of the unit cell structure viewed along *a*-axis (**3** = gray, anthracene = pink, dichloromethane = green).

Table S25. Crystal data and structure refinement for **3•ANT•**CH_2Cl_2.

Chemical formula moiety	C_{44} H_{40} B_4 N_4 O_8 S_2, C_{14} H_{10}, C H_2 Cl_2
Chemical formula sum	C_{59} H_{52} B_4 Cl_2 N_4 O_8 S_2
Formula weight	1123.31
Temperature	198(2) K
Wavelength	1.54186 Å
Crystal system	Orthorhombic
Space group	$Cmc2_1$
Unit cell dimensions	a = 18.5357(5) Å
	b = 20.6392(10) Å
	c = 14.1552(7) Å
Volume	5415.3(4) $Å^3$
Z	4
Density (calculated)	1.378 Mg/m^3
Absorption coefficient	2.293 mm^{-1}
F(000)	2336
Crystal size	0.19 x 0.19 x 0.09 mm^3
Theta range for data collection	3.20 to 68.14°.
Index ranges	-22<=h<=21, -20<=k<=24, -15<=l<=17
Reflections collected	30899
Independent reflections	5031 [R(int) = 0.0949]
Completeness to theta = 68.22°	99.7 %
Absorption correction	Semi-empirical from equivalents
Max. and min. transmission	0.8185 and 0.6071
Refinement method	Full-matrix least-squares on F^2
Data / restraints / parameters	5031 / 1 / 366
Goodness-of-fit on F^2	1.142
Final R indices [I>2sigma(I)]	R_1 = 0.0691, wR_2 = 0.1682
R indices (all data)	R_1 = 0.0965, wR_2 = 0.2190
Largest diff. peak and hole	0.600 and -0.506 e.$Å^{-3}$

2) **3•NA**•4$CHCl_3$

The single crystal of **3•NA**•4$CHCl_3$ suitable for X-ray crystallographic analysis was obtained by vapor diffusion of hexane into the chloroform solution of **3•NA**. Each unit cell of the crystal consisted of two **3•NA** complexes and 8 chloroform molecules.

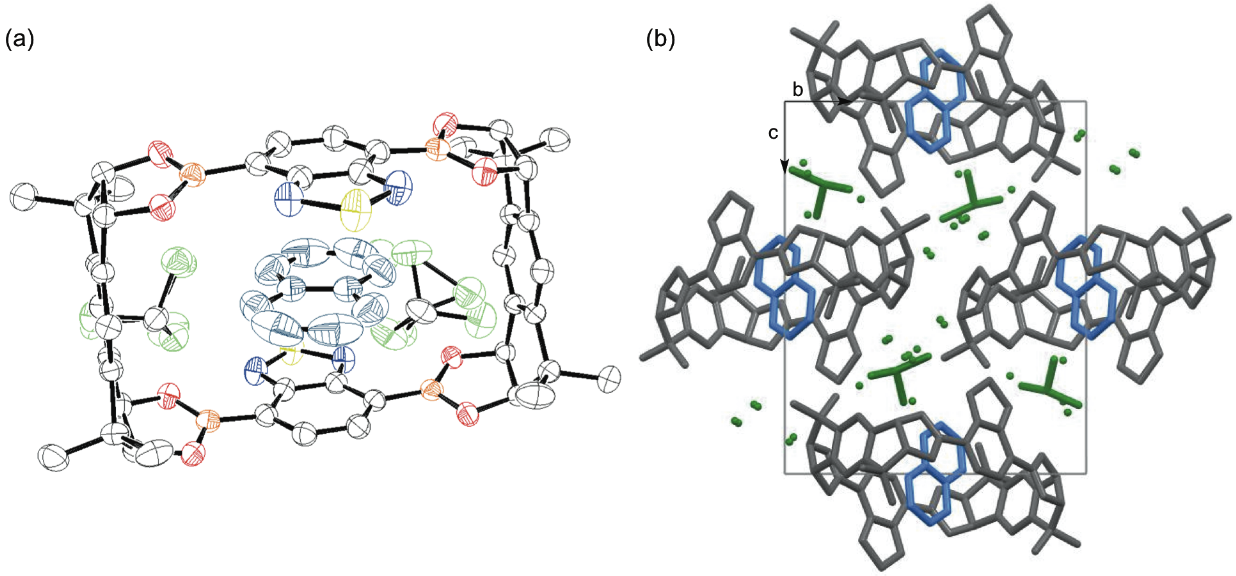

Figure S29. X-ray crystal structure of 3•NA•4$CHCl_3$ (CCDC 1005495). All hydrogen atoms are omitted for clarity. (a) ORTEP structure with thermal ellipsoids shown at 50% probability (C = gray, O = red, B = orange, N = blue, S = yellow, Cl = green, naphthalene = light blue). (b) Schematic representation of the unit cell structure viewed along *a*-axis (**3** = gray, naphthalene = blue, chloroform = green).

Table S26. Crystal data and structure refinement for **3•NA•**$4CHCl_3$.

Chemical formula moiety	$C_{44} H_{40} B_4 N_4 O_8 S_2$, $C_{10} H_8$, $4(C H Cl_3)$
Chemical formula sum	$C_{58} H_{52} B_4 Cl_{12} N_4 O_8 S_2$
Formula weight	1465.79
Temperature	293(2) K
Wavelength	1.54186 Å
Crystal system	Monoclinic
Space group	$P2_1/c$
Unit cell dimensions	a = 13.7340(6) Å
	b = 14.1169(5) Å β = 122.833(2)°.
	c = 20.8001(7) Å
Volume	3388.5(2) Å^3
Z	2
Density (calculated)	1.437 Mg/m^3
Absorption coefficient	5.510 mm^{-1}
F(000)	1496
Crystal size	0.294 x 0.285 x 0.174 mm^3
Theta range for data collection	3.830 to 68.224°.
Index ranges	$-16<=h<=16$, $-16<=k<=17$, $-24<=l<=25$
Reflections collected	37551
Independent reflections	6111 [R(int) = 0.0663]
Completeness to theta = 68.22°	98.6 %
Absorption correction	Semi-empirical from equivalents
Max. and min. transmission	0.447 and 0.383
Refinement method	Full-matrix least-squares on F^2
Data / restraints / parameters	6111 / 30 / 442
Goodness-of-fit on F^2	1.149
Final R indices [I>2sigma(I)]	R_1 = 0.0518, wR_2 = 0.1270
R indices (all data)	R_1 = 0.0630, wR_2 = 0.1371
Largest diff. peak and hole	0.355 and -0.373 e.Å^{-3}

3) **3•ANT**•4$CHCl_3$

The single crystal of **3•ANT**•4$CHCl_3$ suitable for X-ray crystallographic analysis was obtained by vapor diffusion of hexane into the chloroform solution of **3•ANT**. Each unit cell of the crystal consisted of one **3•ANT** complex and four chloroform molecules.

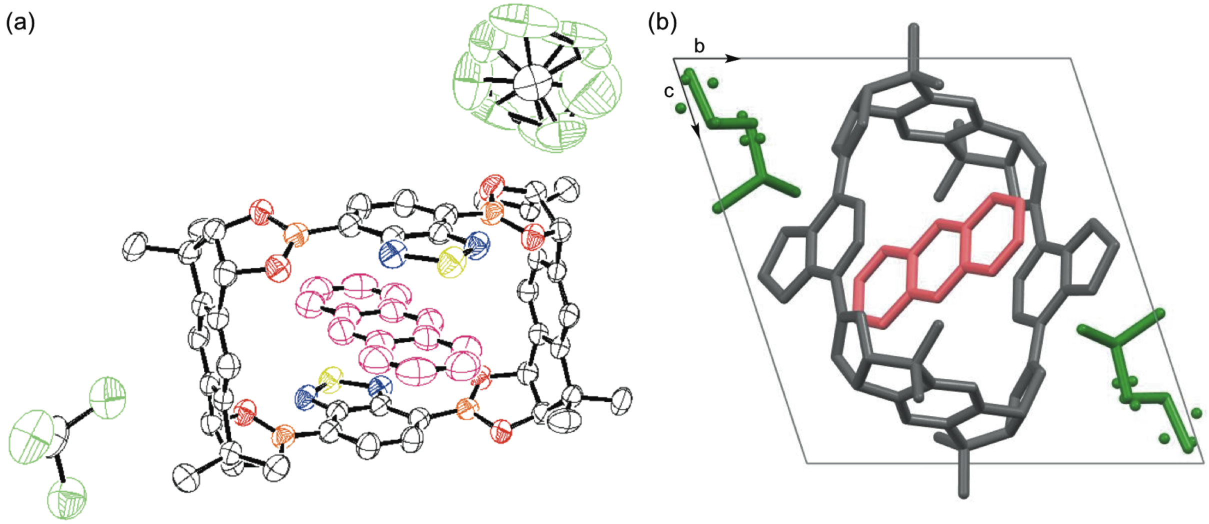

Figure S30. X-ray crystal structure of 3•ANT•4$CHCl_3$ (CCDC 1005496). All hydrogen atoms are omitted for clarity. (a) ORTEP structure with thermal ellipsoids shown at 50% probability (C = gray, O = red, B = orange, N = blue, S = yellow, Cl = green, anthracene = pink). (b) Schematic representation of the unit cell structure viewed along *a*-axis (**3** = gray, anthracene = pink, chloroform = green).

Table S27. Crystal data and structure refinement for **3•ANT**•4CHCl$_3$.

Chemical formula moiety	C_{44} H_{40} B_4 N_4 O_8 S_2, C_{14} H_{10}, 4(C H Cl_3)	
Chemical formula sum	C_{62} H_{54} B_4 Cl_{12} N_4 O_8 S_2	
Formula weight	1515.85	
Temperature	293(2) K	
Wavelength	1.54186 Å	
Crystal system	Triclinic	
Space group	*P*-1	
Unit cell dimensions	*a* = 10.54186(19) Å	α = 69.8702(7)°.
	b = 12.9408(2) Å	β = 73.6990(7)°.
	c = 14.3073(3) Å	γ = 79.7710(7)°.
Volume	1751.79(6) Å^3	
Z	1	
Density (calculated)	1.437 Mg/m^3	
Absorption coefficient	5.350 mm^{-1}	
F(000)	774	
Crystal size	0.20 x 0.09 x 0.09 mm^3	
Theta range for data collection	5.96 to 68.22°.	
Index ranges	-12<=*h*<=12, -15<=*k*<=15, -17<=*l*<=17	
Reflections collected	20272	
Independent reflections	6288 [*R*(int) = 0.0453]	
Completeness to theta = 68.22°	97.9 %	
Absorption correction	Semi-empirical from equivalents	
Max. and min. transmission	0.6446 and 0.5475	
Refinement method	Full-matrix least-squares on F^2	
Data / restraints / parameters	6288 / 7 / 471	
Goodness-of-fit on F^2	1.120	
Final *R* indices [*I*>2sigma(*I*)]	R_1 = 0.0514, wR_2 = 0.1284	
R indices (all data)	R_1 = 0.0769, wR_2 = 0.1637	
Largest diff. peak and hole	0.407 and -0.510 e.Å^{-3}	

4) **3•CHCl₃**•4CHCl₃

The single crystal of **$3\cdot CHCl_3$**$\cdot 4CHCl_3$ suitable for X-ray crystallographic analysis was obtained by vapor diffusion of hexane into the chloroform solution of **3**. Each unit cell of the crystal consisted of two **$3\cdot CHCl_3$** complexes and 8 chloroform molecules.

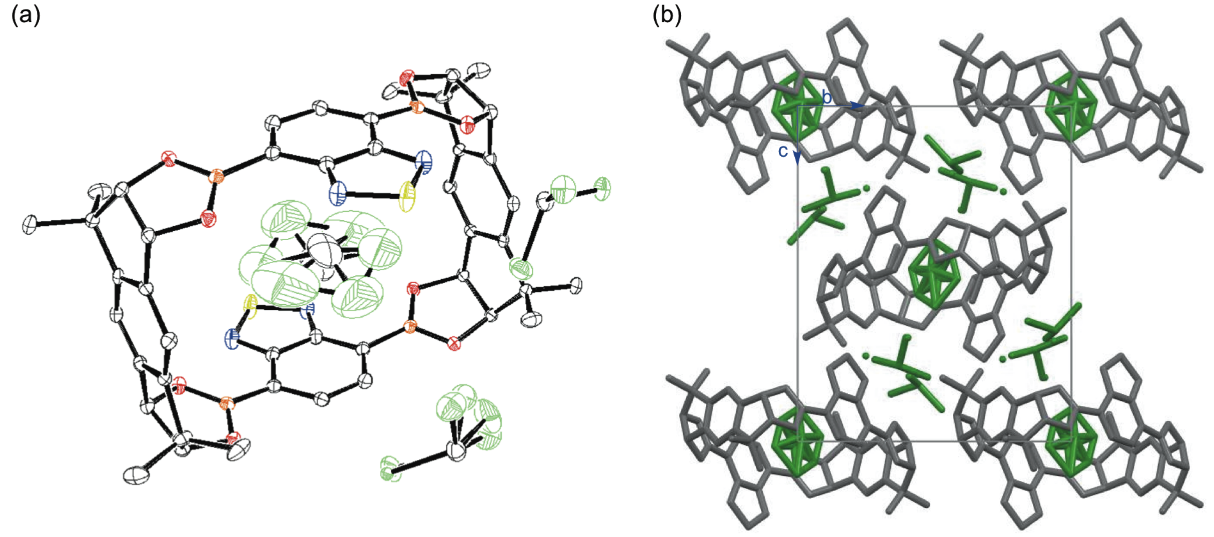

Figure S31. X-ray crystal structure of $3\cdot CHCl_3\cdot 4CHCl_3$ (CCDC 1005497). All hydrogen atoms are omitted for clarity. (a) ORTEP structure with thermal ellipsoids shown at 50% probability (C = gray, O = red, B = orange, N = blue, S = yellow, Cl = green). (b) Schematic representation of the unit cell structure viewed along *a*-axis (**3** = gray, chloroform = green).

Table S28. Crystal data and structure refinement for **3•CHCl$_3$**•4CHCl$_3$.

Chemical formula moiety	$C_{44} H_{40} B_4 N_4 O_8 S_2$, C H Cl_3, 4(C H Cl_3)	
Chemical formula sum	$C_{49} H_{45} B_4 Cl_{15} N_4 O_8 S_2$	
Formula weight	1457.00	
Temperature	93(2) K	
Wavelength	1.54186 Å	
Crystal system	Monoclinic	
Space group	$P2_1/c$	
Unit cell dimensions	a = 13.7207(3) Å	
	b = 14.0837(3) Å	β = 122.8400(10)°.
	c = 20.4986(3) Å	
Volume	3328.08(12) Å^3	
Z	2	
Density (calculated)	1.454 Mg/m^3	
Absorption coefficient	6.686 mm^{-1}	
F(000)	1476	
Crystal size	0.178 x 0.112 x 0.104 mm^3	
Theta range for data collection	3.835 to 68.228°.	
Index ranges	$-15<=h<=16$, $-16<=k<=16$, $-24<=l<=24$	
Reflections collected	37340	
Independent reflections	6087 [R(int) = 0.0609]	
Completeness to theta = 67.686°	100.0 %	
Absorption correction	Semi-empirical from equivalents	
Max. and min. transmission	1.0000 and 0.8226	
Refinement method	Full-matrix least-squares on F^2	
Data / restraints / parameters	6087 / 24 / 405	
Goodness-of-fit on F^2	1.077	
Final R indices [I>2sigma(I)]	R_1 = 0.0481, wR_2 = 0.1183	
R indices (all data)	R_1 = 0.0536, wR_2 = 0.1233	
Largest diff. peak and hole	0.669 and -0.827 e.Å^{-3}	

5) **3•BT**•4$CHCl_3$

The single crystal of **3•BT**•4$CHCl_3$ suitable for X-ray crystallographic analysis was obtained by vapor diffusion of hexane into the chloroform solution of **3•BT**. Each unit cell of the crystal consisted of two **3•BT** complexes and 8 chloroform molecules.

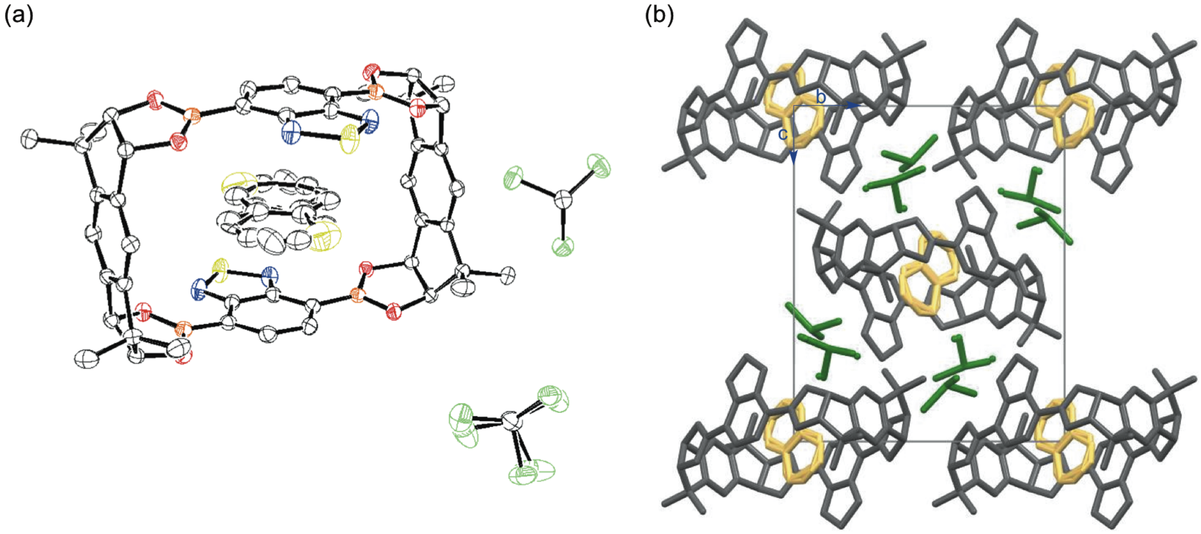

Figure S32. X-ray crystal structure of 3•BT•4$CHCl_3$ (CCDC 1005498). All hydrogen atoms are omitted for clarity. (a) ORTEP structure with thermal ellipsoids shown at 50% probability (C = gray, O = red, B = orange, N = blue, S = yellow, Cl = green). (b) Schematic representation of the unit cell structure viewed along *a*-axis (**3** = gray, benzothiophene = yellow, chloroform = green).

Table S29. Crystal data and structure refinement for **3•BT**•4CHCl$_3$.

Chemical formula moiety	$C_{44} H_{40} B_4 N_4 O_8 S_2$, $C_8 H_6 S$, 4(C H Cl_3)
Chemical formula sum	$C_{56} H_{50} B_4 Cl_{12} N_4 O_8 S_3$
Formula weight	1471.82
Temperature	173(2) K
Wavelength	0.71075 Å
Crystal system	Monoclinic
Space group	$P2_1/c$
Unit cell dimensions	a = 13.6699(7) Å
	b = 14.0308(9) Å β = 122.572(3)°.
	c = 20.6325(9) Å
Volume	3334.9(3) Å^3
Z	2
Density (calculated)	1.466 Mg/m^3
Absorption coefficient	0.646 mm^{-1}
F(000)	1500
Crystal size	0.13 x 0.11 x 0.09 mm^3
Theta range for data collection	3.01 to 27.41°.
Index ranges	-17<=h<=16, -18<=k<=18, -26<=l<=26
Reflections collected	52063
Independent reflections	7560 [R(int) = 0.0593]
Completeness to theta = 68.22°	99.5 %
Absorption correction	Semi-empirical from equivalents
Max. and min. transmission	0.9459 and 0.7456
Refinement method	Full-matrix least-squares on F^2
Data / restraints / parameters	7560 / 54 / 418
Goodness-of-fit on F^2	1.047
Final R indices [I>2sigma(I)]	R_1 = 0.0503, wR_2 = 0.1339
R indices (all data)	R_1 = 0.0704, wR_2 = 0.1473
Largest diff. peak and hole	0.797 and -0.957 e.Å^{-3}

6) **3•BF**•4$CHCl_3$

The single crystal of **3•BF**•4$CHCl_3$ suitable for X-ray crystallographic analysis was obtained by vapor diffusion of hexane into the chloroform solution of **3•BF**. Each unit cell of the crystal consisted of two **3•BF** complexes and 8 chloroform molecules.

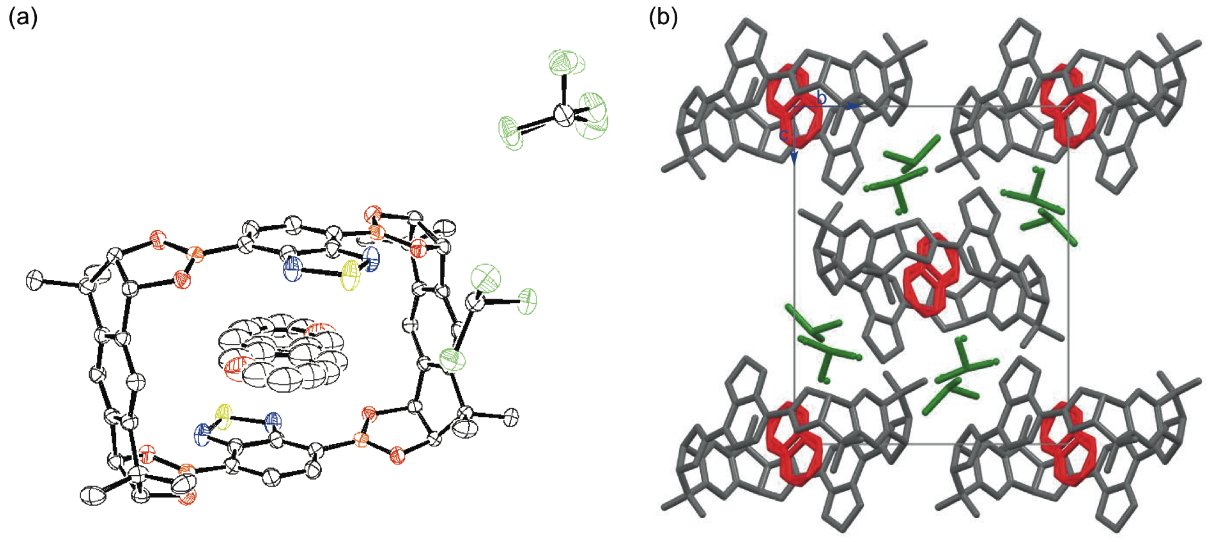

Figure S33. X-ray crystal structure of 3•BF•4$CHCl_3$ (CCDC 1005499). All hydrogen atoms are omitted for clarity. (a) ORTEP structure with thermal ellipsoids shown at 50% probability (C = gray, O = red, B = orange, N = blue, S = yellow, Cl = green). (b) Schematic representation of the unit cell structure viewed along *a*-axis (**3** = gray, benzofuran = red, chloroform = green).

Table S30. Crystal data and structure refinement for **3•BF**•4CHCl$_3$.

Chemical formula moiety	C_{44} H_{40} B_4 N_4 O_8 S_2, C_8 H_6 O, 4(C H Cl_3)
Empirical formula	C_{56} H_{50} B_4 Cl_{12} N_4 O_9 S_2
Formula weight	1455.76
Temperature	173(2) K
Wavelength	0.71075 Å
Crystal system	Monoclinic
Space group	$P2_1/c$
Unit cell dimensions	a = 13.6823(12) Å
	b = 14.0543(13) Å β = 122.711(4)°.
	c = 20.5851(14) Å
Volume	3330.6(5) Å^3
Z	2
Density (calculated)	1.452 Mg/m^3
Absorption coefficient	0.617 mm^{-1}
F(000)	1484
Crystal size	0.19 x 0.10 x 0.09 mm^3
Theta range for data collection	3.01 to 27.42°.
Index ranges	$-17<=h<=17$, $-18<=k<=18$, $-26<=l<=26$
Reflections collected	51293
Independent reflections	7581 [R(int) = 0.0455]
Completeness to theta = 68.22°	99.7 %
Absorption correction	Semi-empirical from equivalents
Max. and min. transmission	0.9483 and 0.7825
Refinement method	Full-matrix least-squares on F^2
Data / restraints / parameters	7581 / 0 / 405
Goodness-of-fit on F^2	1.050
Final R indices [I>2sigma(I)]	R_1 = 0.0502, wR_2 = 0.1366
R indices (all data)	R_1 = 0.0614, wR_2 = 0.1455
Largest diff. peak and hole	0.990 and -0.783 e.Å^{-3}

7) **3•QU**•4$CHCl_3$

The single crystal of **3•QU**•4$CHCl_3$ suitable for X-ray crystallographic analysis was obtained by vapor diffusion of hexane into the chloroform solution of **3•QU**. Each unit cell of the crystal consisted of two **3•QU** complexes and 8 chloroform molecules.

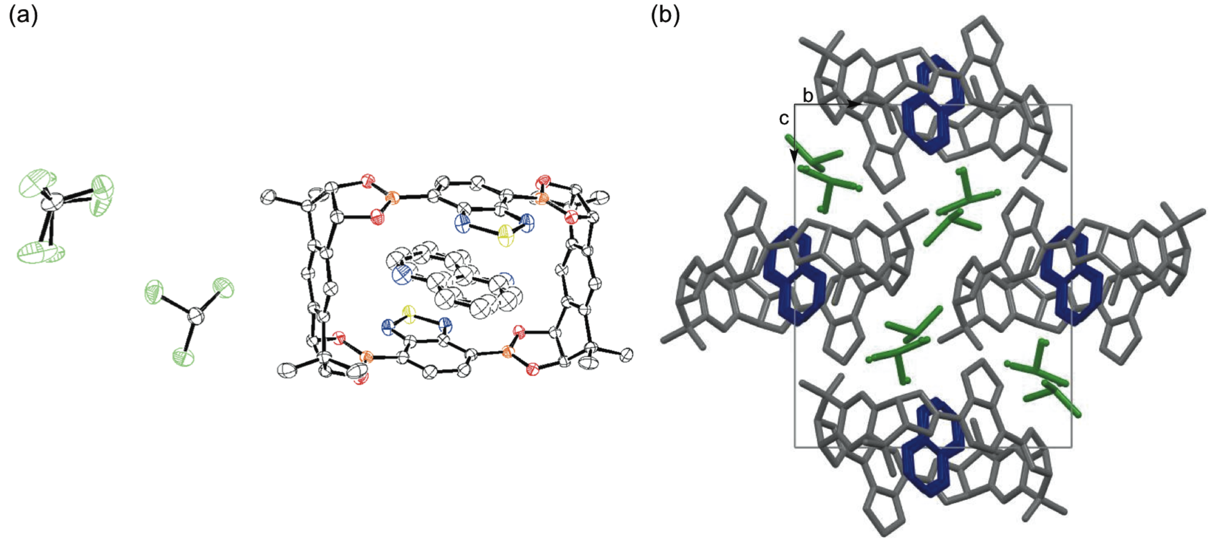

Figure S34. X-ray crystal structure of 3•QU•4$CHCl_3$ (CCDC 1005500). All hydrogen atoms are omitted for clarity. (a) ORTEP structure with thermal ellipsoids shown at 50% probability (C = gray, O = red, B = orange, N = blue, S = yellow, Cl = green). (b) Schematic representation of the unit cell structure viewed along *a*-axis (**3** = gray, quinoline = blue, chloroform = green).

Table S31. Crystal data and structure refinement for **3•QU**•4CHCl$_3$.

Chemical formula moiety	C_{44} H_{40} B_4 N_4 O_8 S_2, C_9 H_7 N, 4(C H Cl_3)
Chemical formula sum	C_{57} H_{51} B_4 Cl_{12} N_5 O_8 S_2
Formula weight	1466.79
Temperature	173(2) K
Wavelength	0.71075 Å
Crystal system	Monoclinic
Space group	$P2_1/c$
Unit cell dimensions	a = 13.6829(10) Å
	b = 14.0362(10) Å β = 122.667(3)°.
	c = 20.6735(11) Å
Volume	3342.4(4) Å^3
Z	2
Density (calculated)	1.457 Mg/m^3
Absorption coefficient	0.615 mm^{-1}
F(000)	1496
Crystal size	0.50 x 0.42 x 0.39 mm^3
Theta range for data collection	3.01 to 27.44°.
Index ranges	$-17<=h<=17$, $-18<=k<=18$, $-26<=l<=26$
Reflections collected	51895
Independent reflections	7625 [R(int) = 0.0494]
Completeness to theta = 68.22°	99.8 %
Absorption correction	Semi-empirical from equivalents
Max. and min. transmission	0.7964 and 0.7497
Refinement method	Full-matrix least-squares on F^2
Data / restraints / parameters	7625 / 0 / 429
Goodness-of-fit on F^2	1.050
Final R indices [I>2sigma(I)]	R_1 = 0.0457, wR_2 = 0.1219
R indices (all data)	R_1 = 0.0556, wR_2 = 0.1289
Largest diff. peak and hole	0.678 and -0.339 e.Å^{-3}

6. Miscellaneous Data

6-1. Effect of solvent in the self-assembly of **3** in the presence of **NA** and **ANT** (Table S32)

Various cosolvents were examined in the self-assembly of **3** in the presence of naphthalene and anthracene (Table S32). When the common organic solvents (CH_2Cl_2, THF, Et_2O, etc.) were used as cosolvents, anthracene was selectively included in the precipitate of **3** (Table S32, entries 1–7). In contrast, naphthalene was selectively included in **3** by using $CHCl_3$ as cosolvent (entries 8 and 9).

Table S32. Effect of solvent in the self-assembly of **3** in the presence of **NA** and **ANT**.

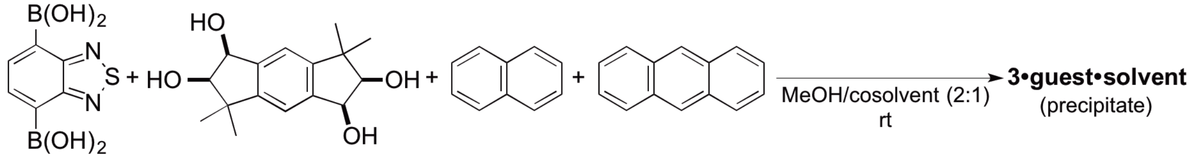

1 — *rac*-**2** (1.0 mol. amt.) — **NA** (1.0 mol. amt.) — **ANT** (1.0 mol. amt.)

entry	cosolvent	time (h)	product[a]	yield (%)
1	CH_2Cl_2	3	**3**•**ANT**•0.5CH_2Cl_2[b]	95
2	THF	3	**3**•**ANT**•0.5THF[b]	95
3	Et_2O	3	**3**•**ANT**[b]	91
4	CH_3CN	3	**3**•**ANT**[b]	94
5	acetone	3	**3**•**ANT**•0.4acetone[b]	80
6	benzene	4	**3**•0.8**ANT**•0.8benzene[b]	94
7	toluene	4.5	**3**•0.85**ANT**•0.17toluene[b]	87
8	$CHCl_3$	3	**3**•**NA**•3.4$CHCl_3$[c]	89
9	$CHCl_3$	24	**3**•**NA**•4$CHCl_3$[d]	90

[a] Amount of guest molecules and solvents were determined by ^1H NMR analysis. The amount of solvent was variable. [b] The ratio of anthracene/naphthalene was determined to be >20:1 by ^1H NMR analysis. [c] Anthracene (0.4 mol. amt.) was included in the precipitate. [d] Trace amount of anthracene was included in the precipitate.

6-2. Schematic representation of **3•ANT•**$4CHCl_3$ in *P*2_1/*c* crystal structure.

When naphthalene molecule of **3•NA•**$4CHCl_3$ in *P*2_1/*c* crystal structure was virtually replaced to anthracene molecule, large steric hindrances were observed between the anthracene molecule and chloroform molecules (Figure S35).

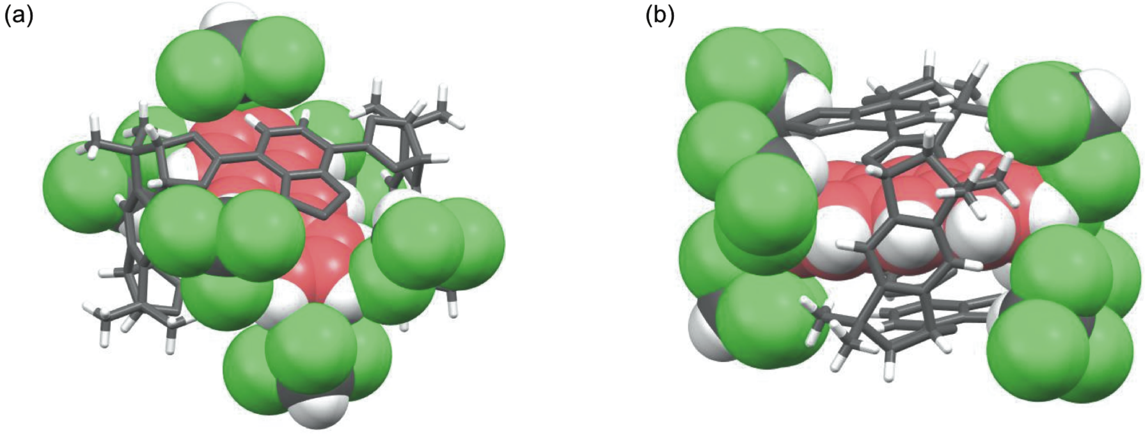

Figure S35. Virtual schematic representation of **3•ANT•**$4CHCl_3$ in *P*2_1/*c* crystal structure. Included anthracene molecule and chloroform molecules are shown as space-filling model, and **3** is represented as stick model. (a) Top view. (b) Side view.

6-3. Self-assembly of **3** using chloroform as cosolvent

When **1**, *rac*-**2**, and anthracene were mixed in MeOH/$CHCl_3$ (2:1) at room temperature for 24 h, **3•ANT**•4$CHCl_3$ was obtained as a precipitate in 97% yield (Figure S36a). Self-assembly of **1** with *rac*-**2** in MeOH/$CHCl_3$ (2:1) at room temperature for 24 h afforded **3•$CHCl_3$**•4$CHCl_3$ in 96% yield as a precipitate (Figure S36b).

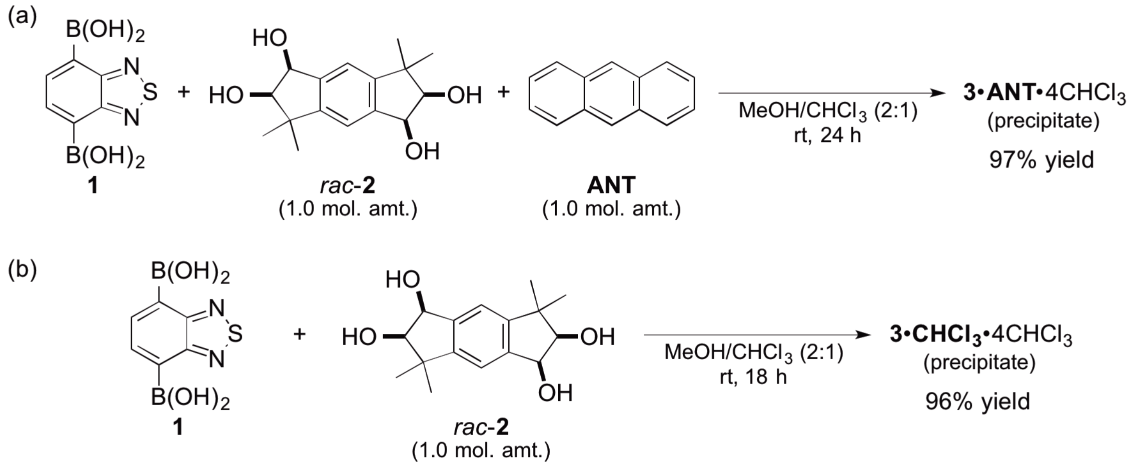

Figure S36. Self-assembly of **3** in MeOH/$CHCl_3$ (2:1). (a) With anthracene (1.0 mol. amt.). (b) Without the addition of guest compound.

References

1) X. Ding, L. Chen, Y. Honsho, X. Feng, O. Saengsawang, J. Guo, A. Saeki, S. Seki, S. Irle, S. Nagase, V. Parasuk and D. Jiang, *J. Am. Chem. Soc.*, 2011, **133**, 14510–14513.

2) H. Sakurai, N. Iwasawa and K. Narasaka, *Bull. Chem. Soc. Jpn.*, 1996, **69**, 2585–2594.

^1H NMR spectrum of **3** (500 MHz, in $CDCl_3$ with 0.03% TMS (v/v), rt)

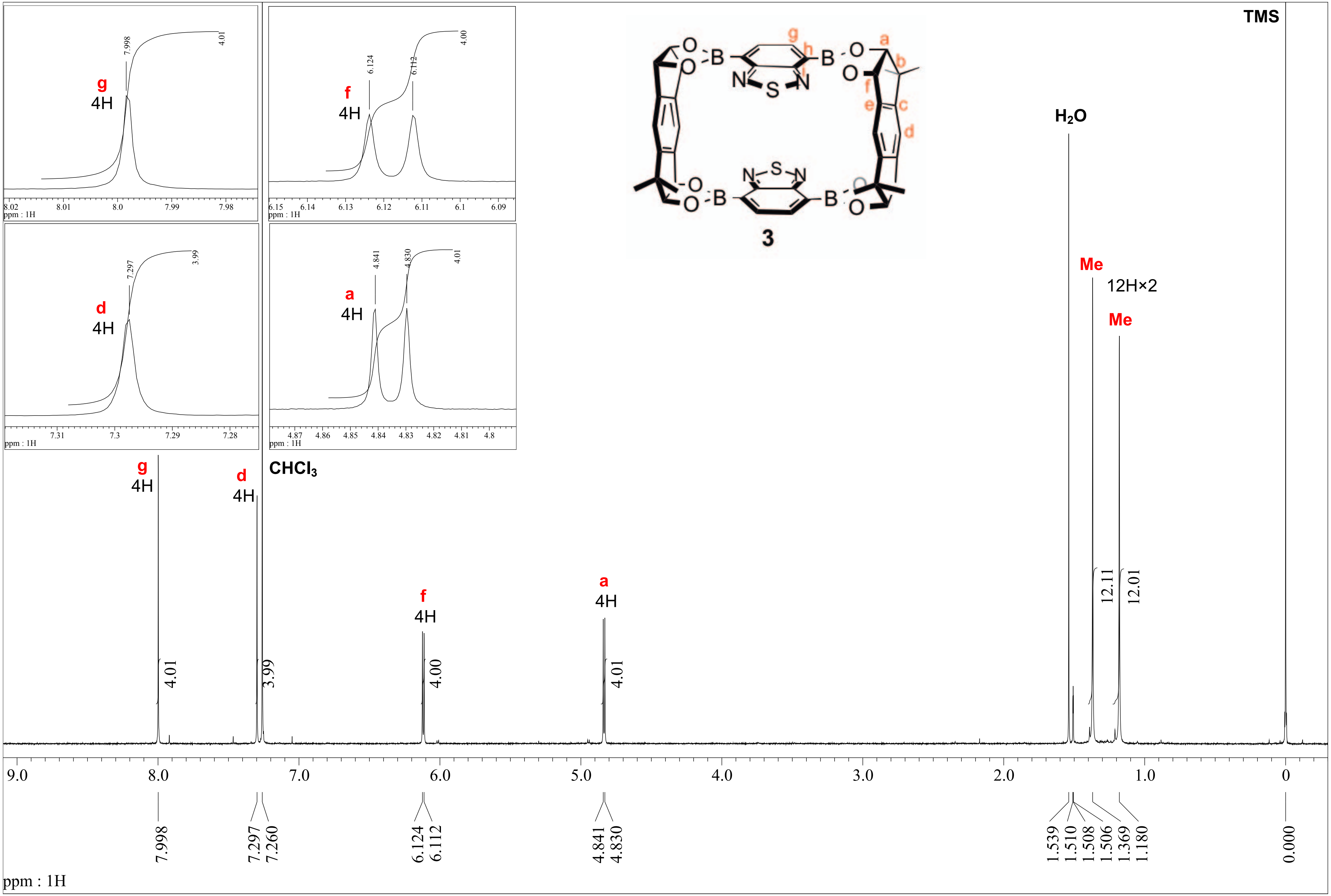

^{13}C NMR spectrum of **3** (125 MHz, in $CDCl_3$ with 0.03% TMS (v/v), rt)

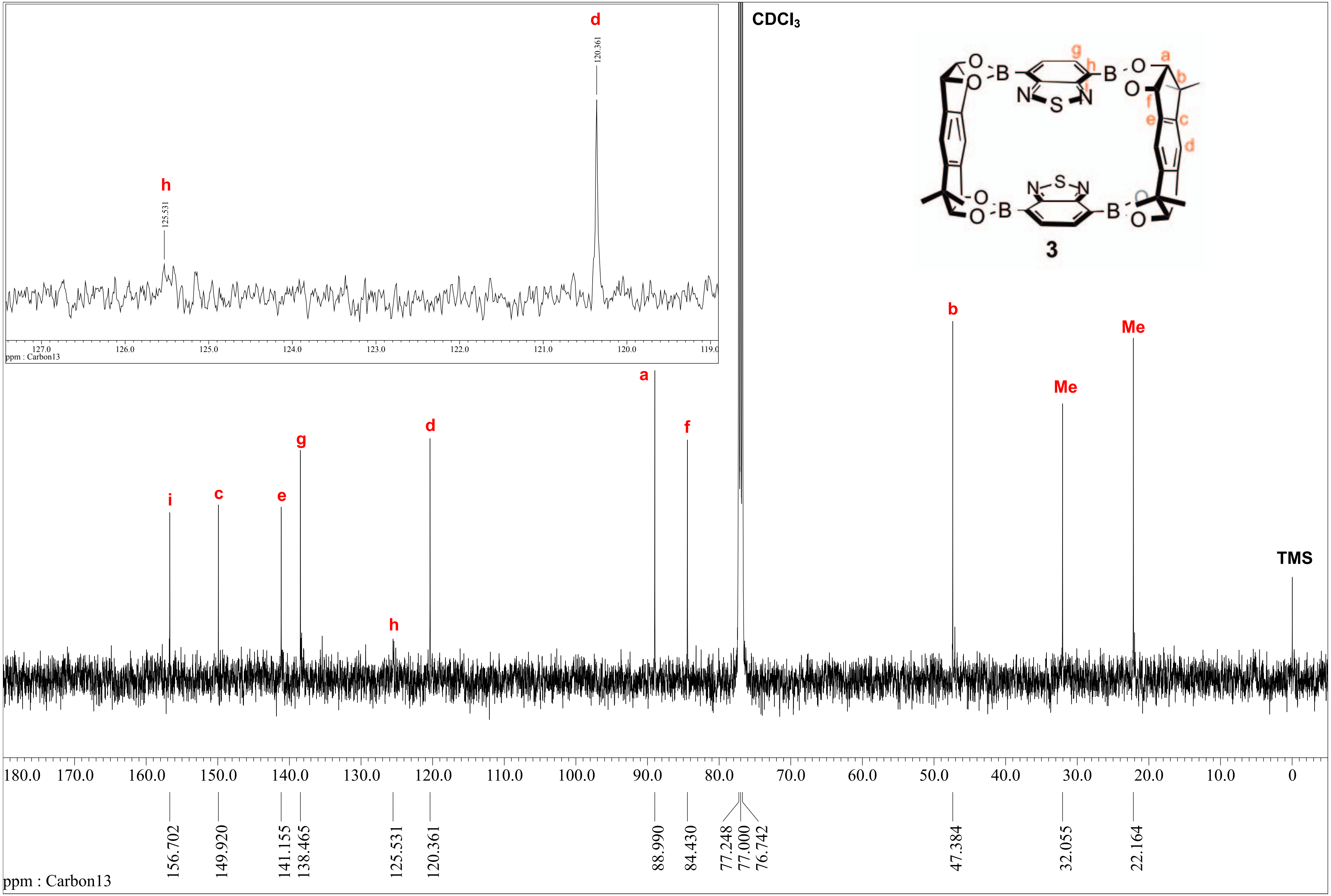

HMQC spectrum of **3** (500 MHz, in $CDCl_3$ with 0.03% TMS (v/v), rt)

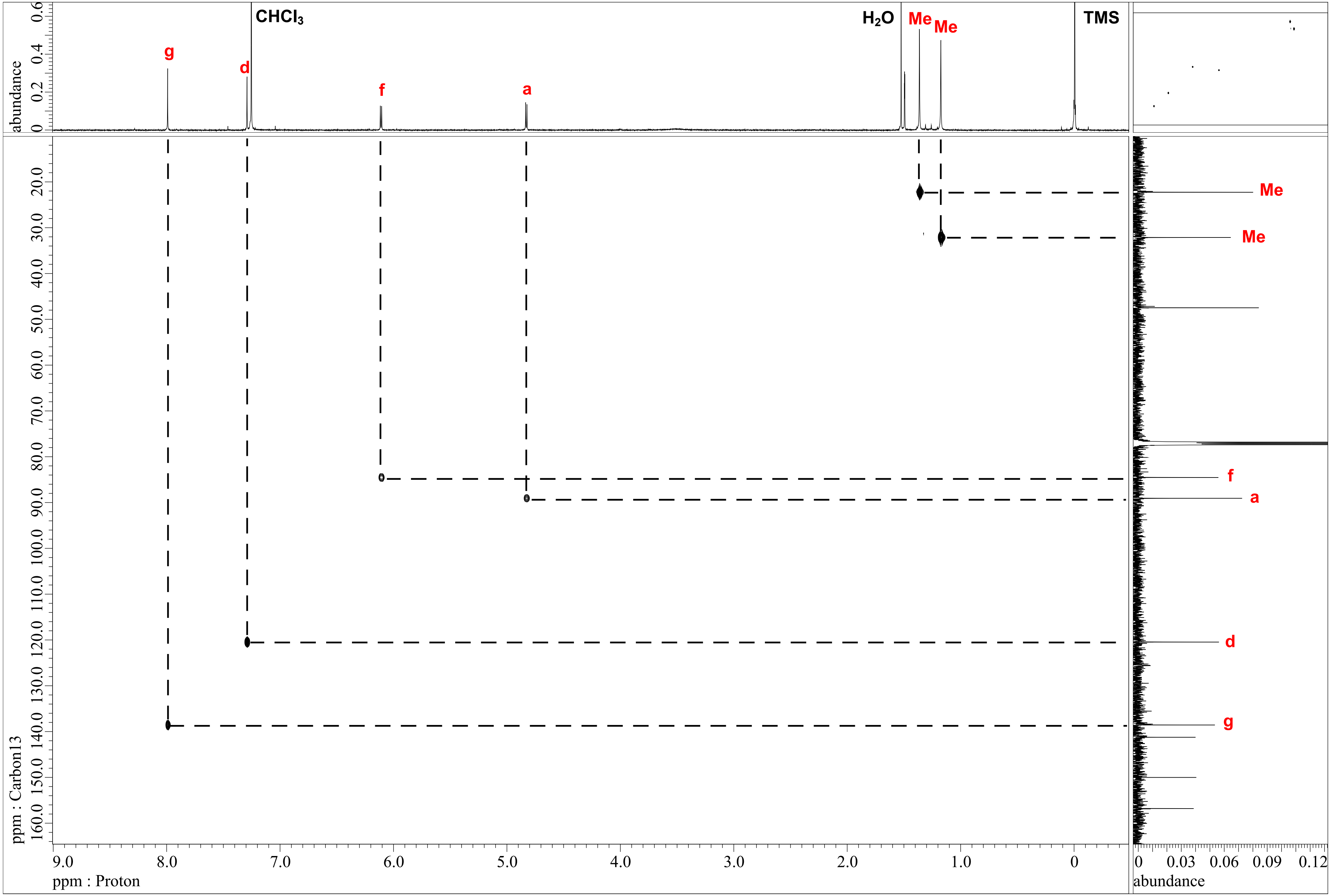

HMBC spectrum of **3** (500 MHz, in $CDCl_3$ with 0.03% TMS (v/v), rt)

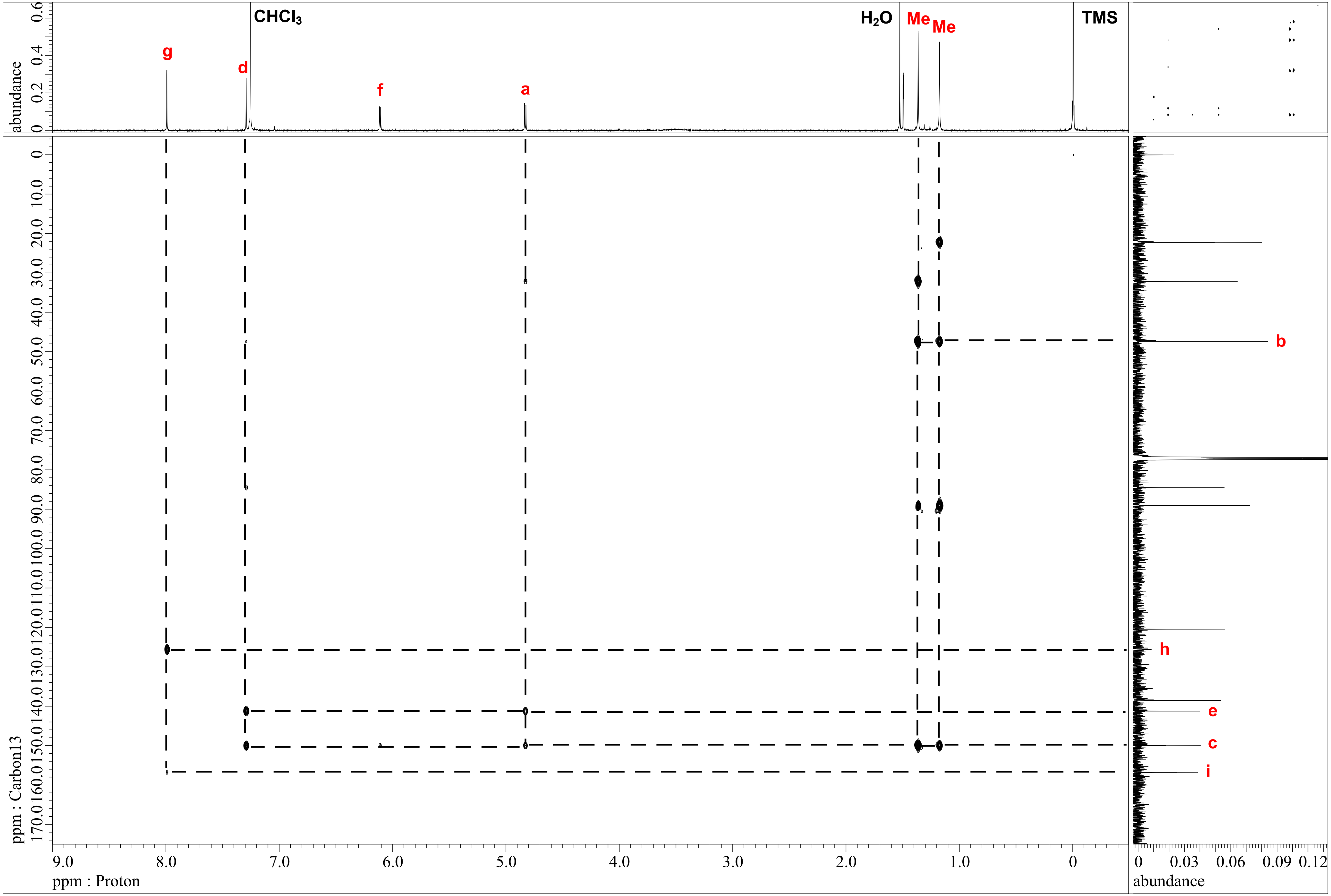

^{1}H NMR spectrum of **3•ANT•CH$_2$Cl$_2$** (500 MHz, in CDCl$_3$ with 0.03% TMS (v/v), rt)

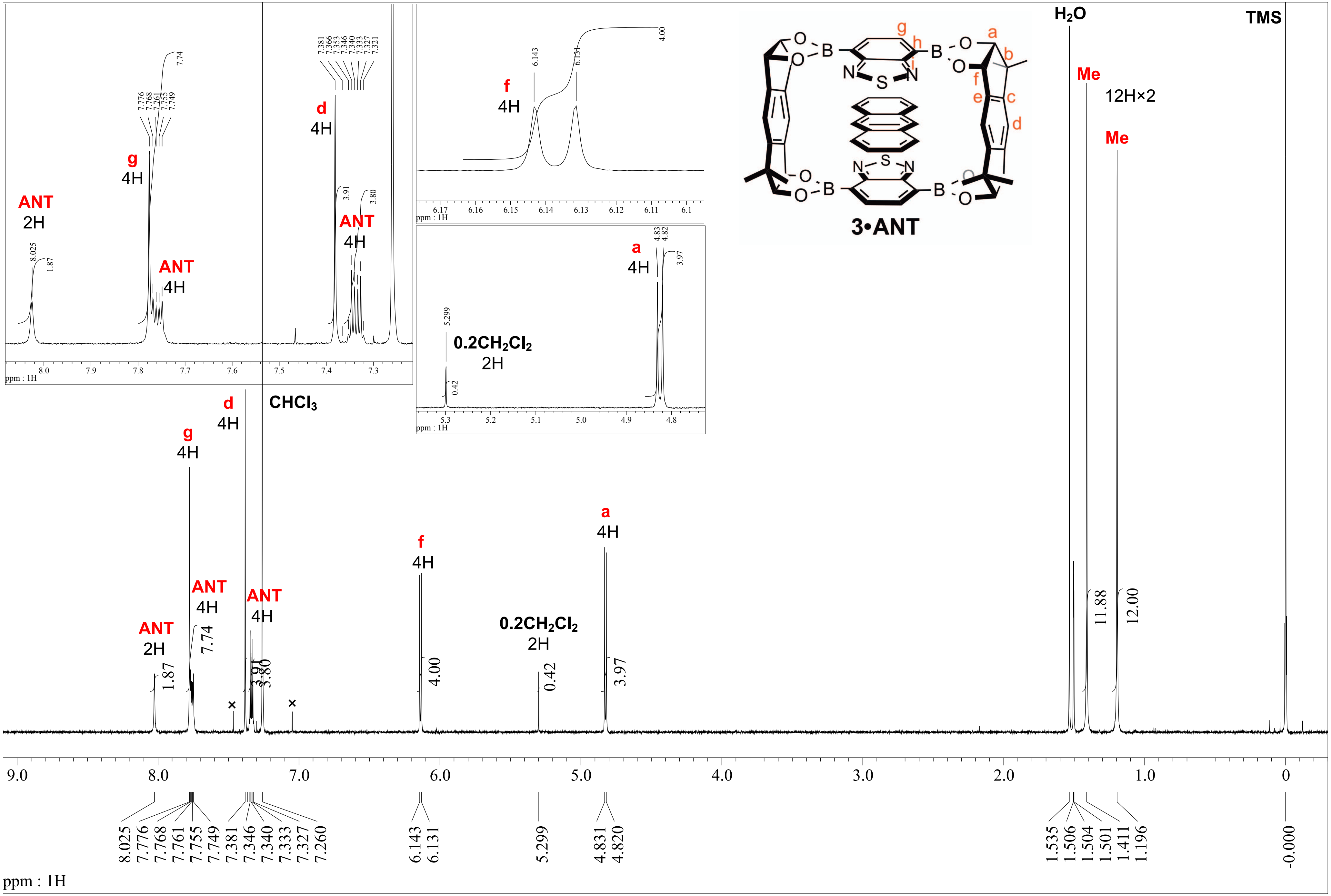

^{1}H NMR spectrum of **3•NA•CHCl₃** (500 MHz, in $CDCl_3$ with 0.03% TMS (v/v), rt)

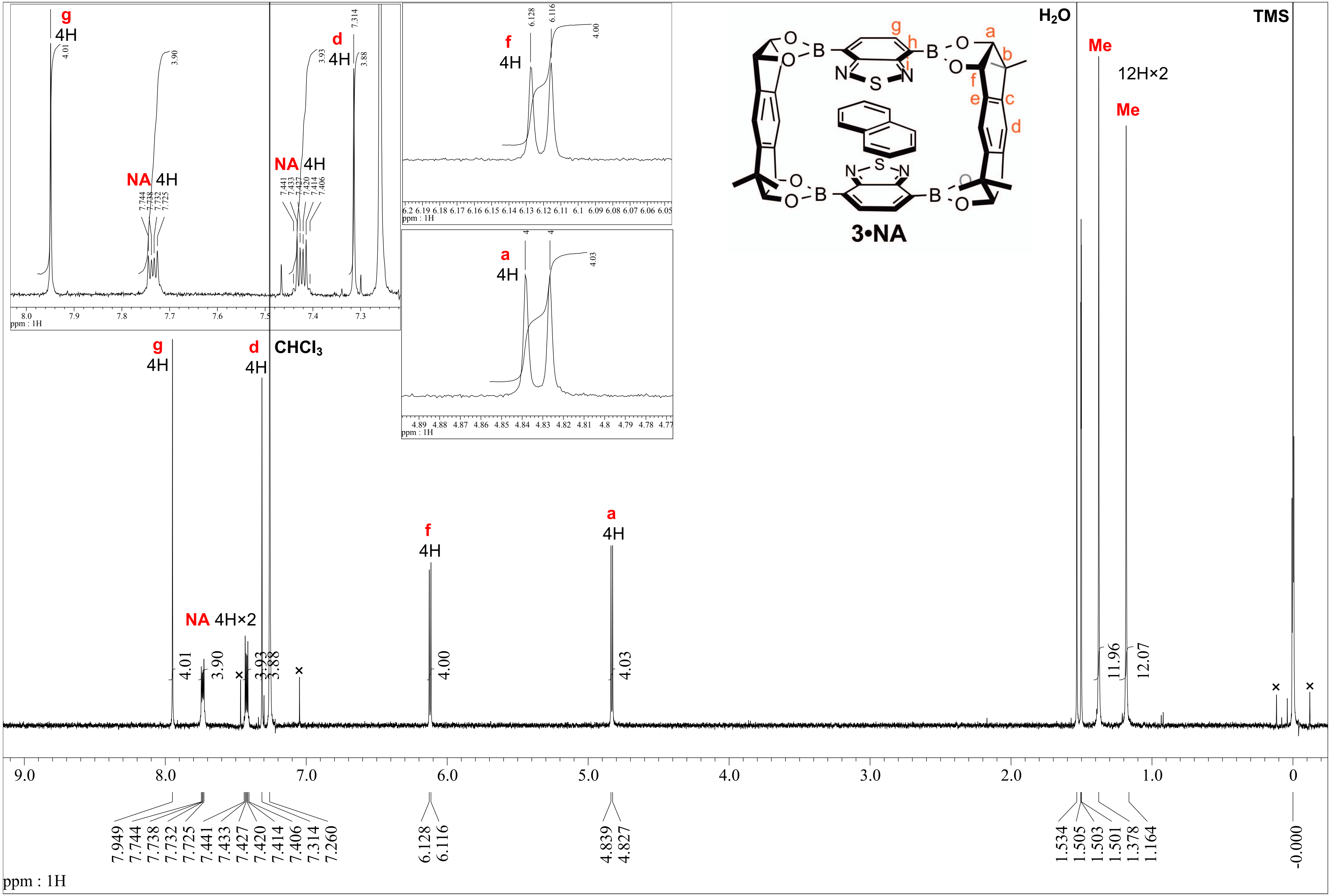

^{1}H NMR spectrum of **3•DBT•CH_2Cl_2** (500 MHz, in $CDCl_3$ with 0.03% TMS (v/v), rt)

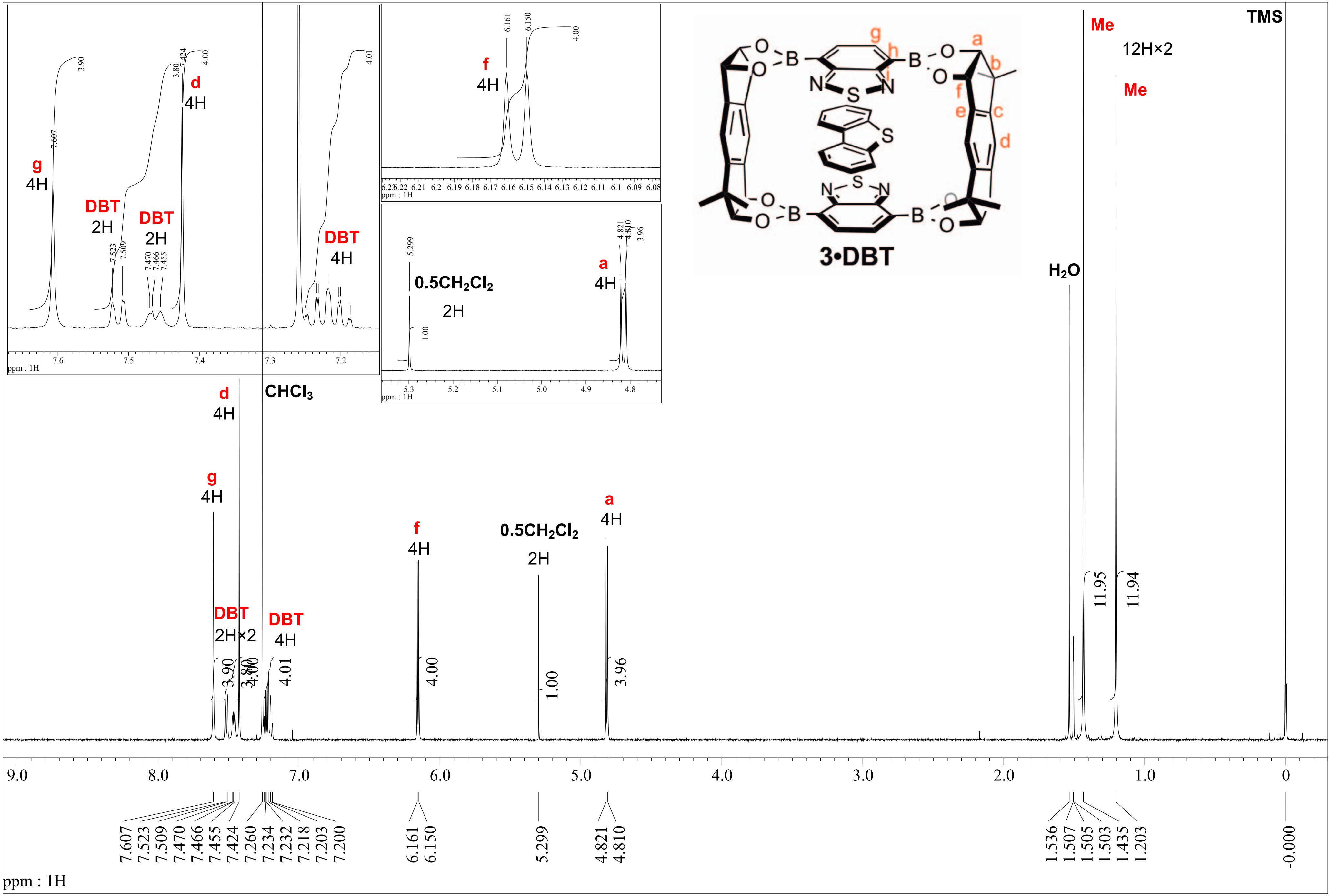

^{1}H NMR spectrum of **3•BT•CHCl$_3$** (500 MHz, in CDCl$_3$ with 0.03% TMS (v/v), rt)

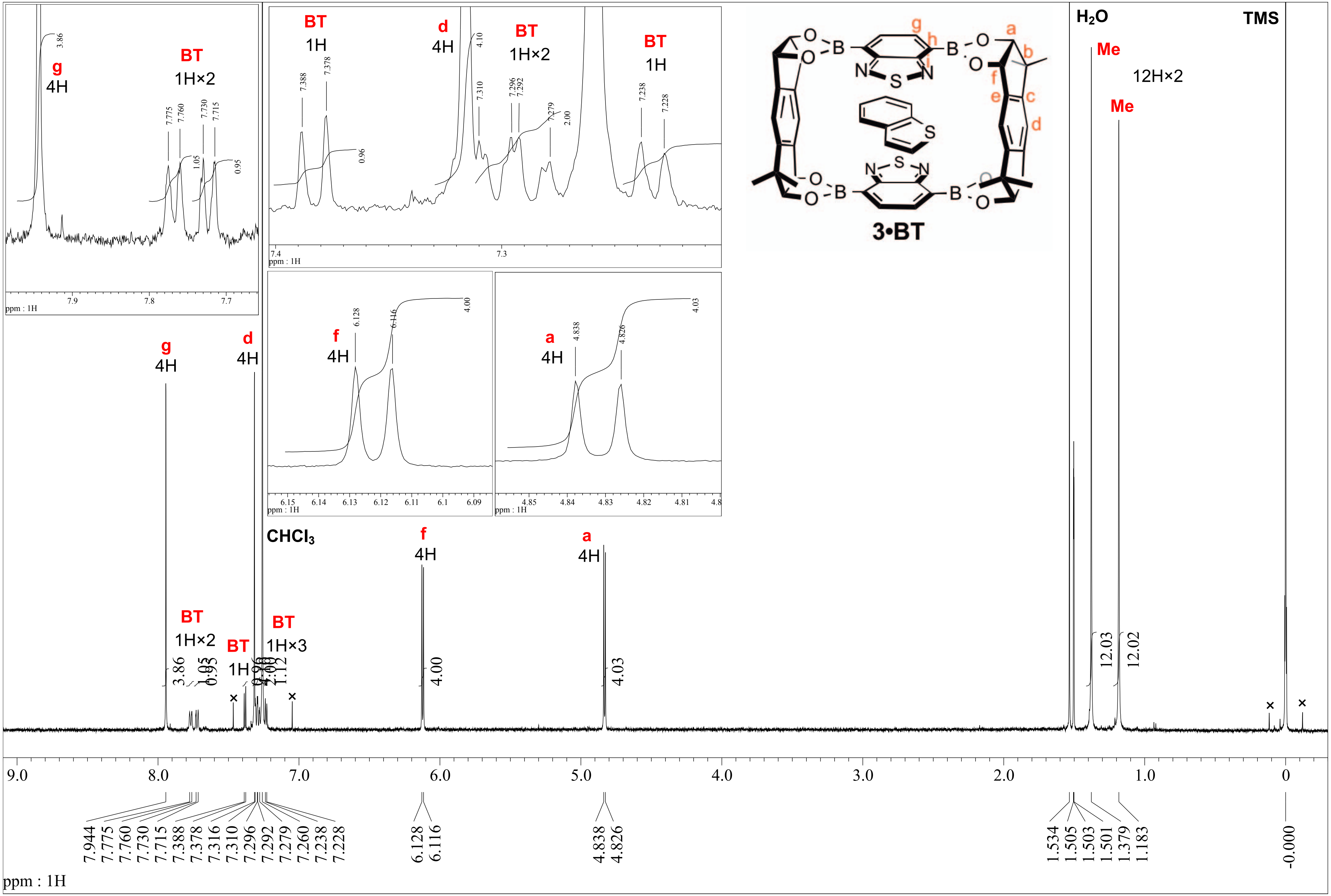

^{1}H NMR spectrum of **3•DBF•CH_2Cl_2** (500 MHz, in $CDCl_3$ with 0.03% TMS (v/v), rt)

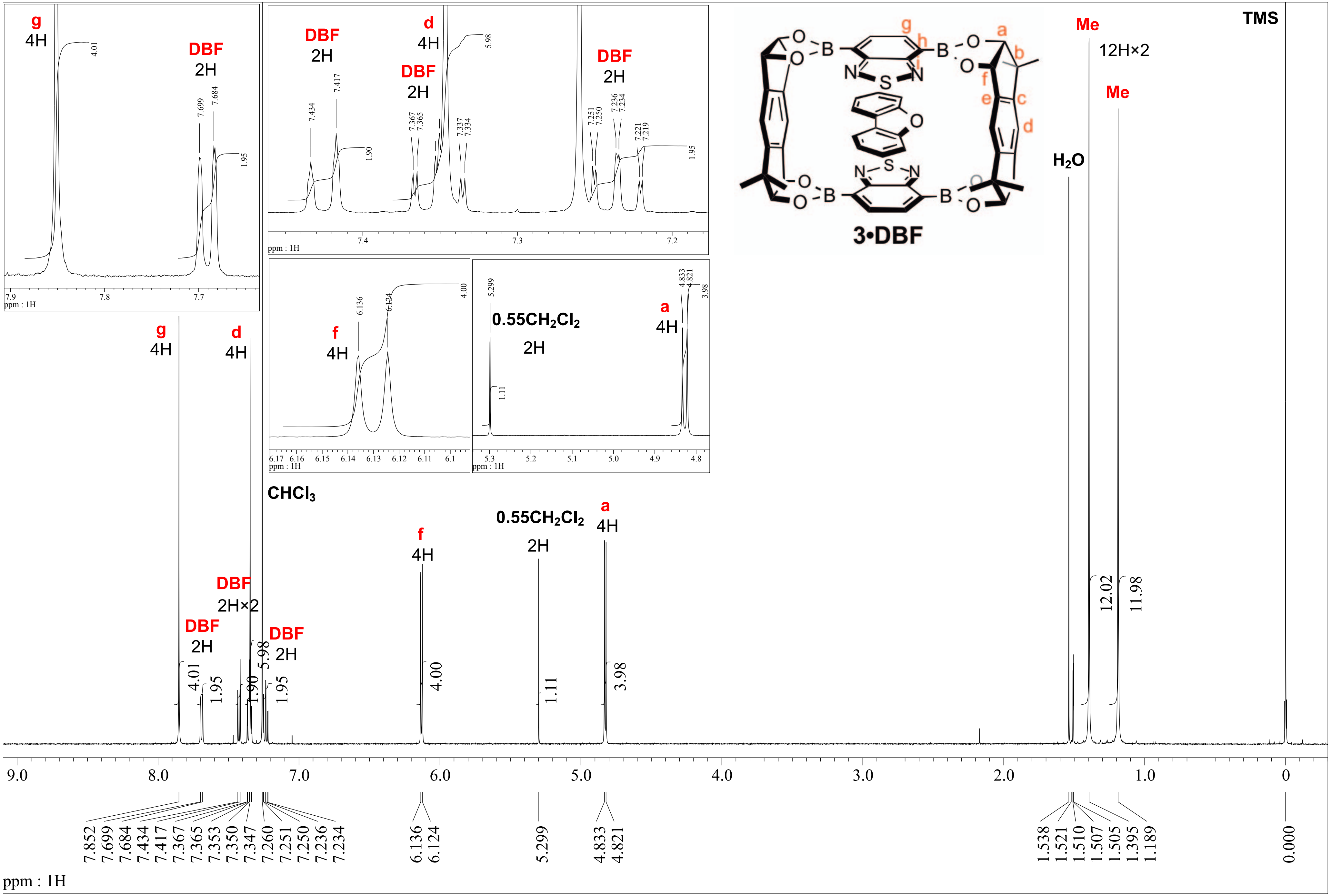

^{1}H NMR spectrum of **3•BF•CHCl$_3$** (500 MHz, in $CDCl_3$ with 0.03% TMS (v/v), rt)

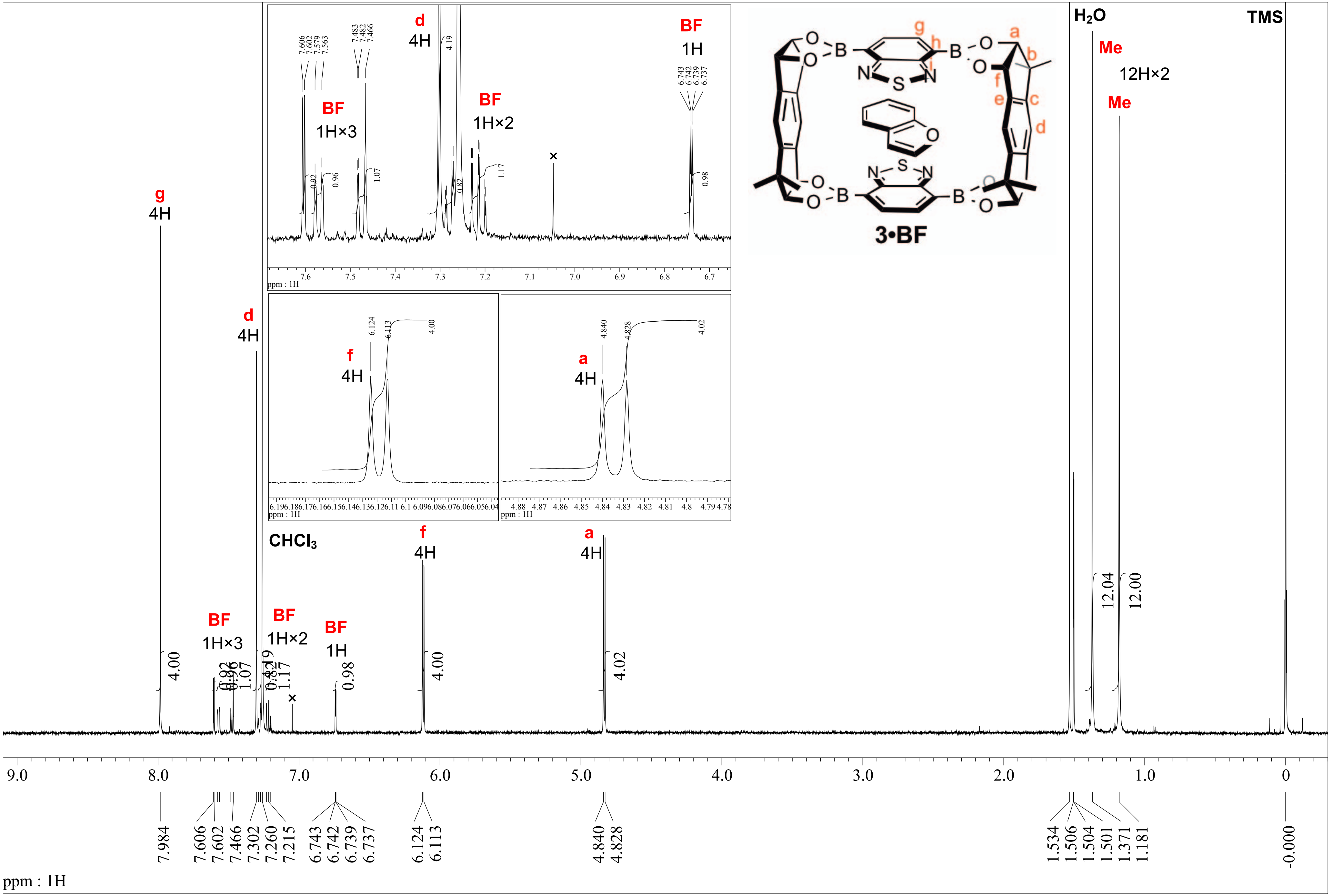

^{1}H NMR spectrum of **3•ACR•CH_2Cl_2** (500 MHz, in $CDCl_3$ with 0.03% TMS (v/v), rt)

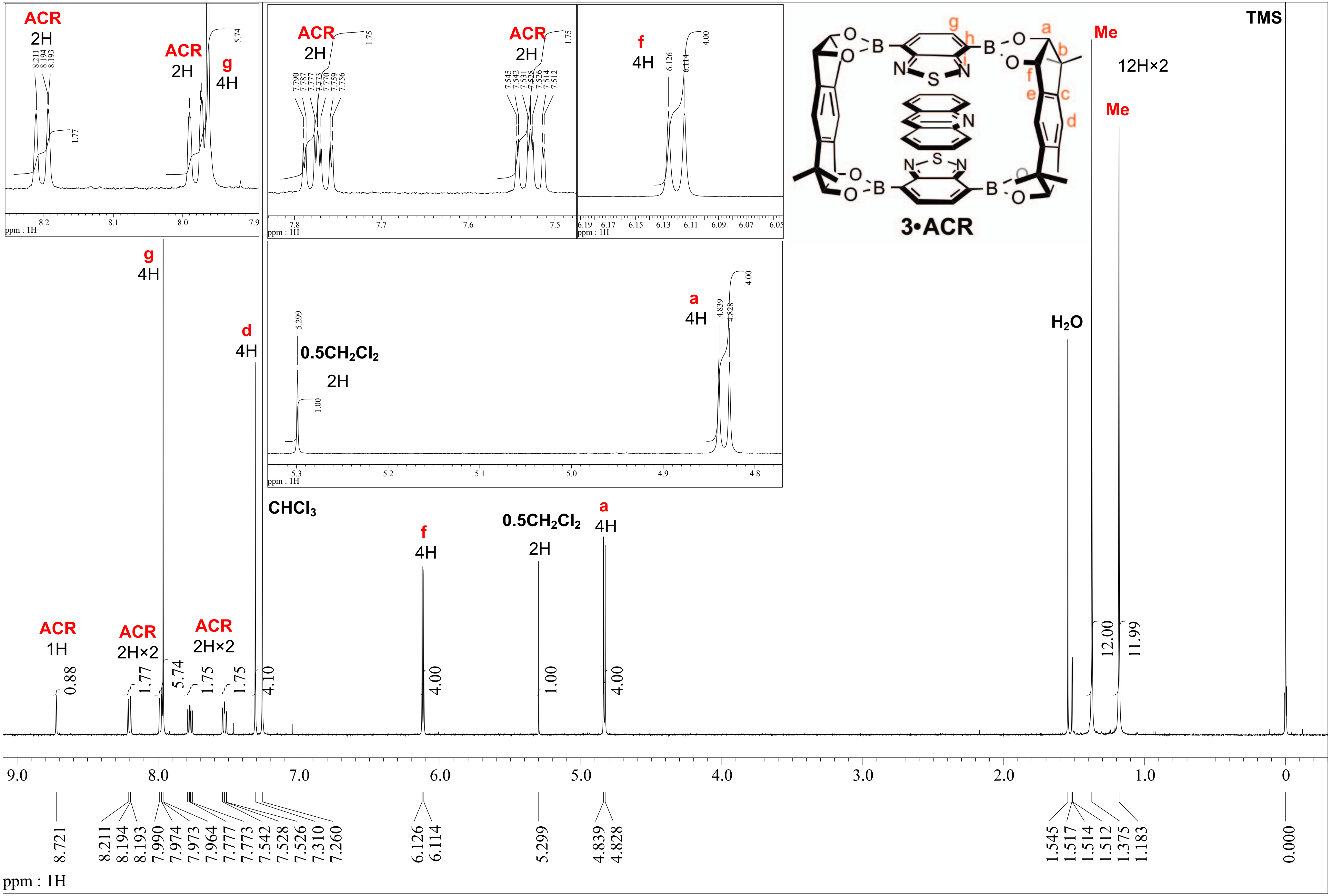

^{1}H NMR spectrum of **3•QU•CHCl₃** (500 MHz, in $CDCl_3$ with 0.03% TMS (v/v), rt)

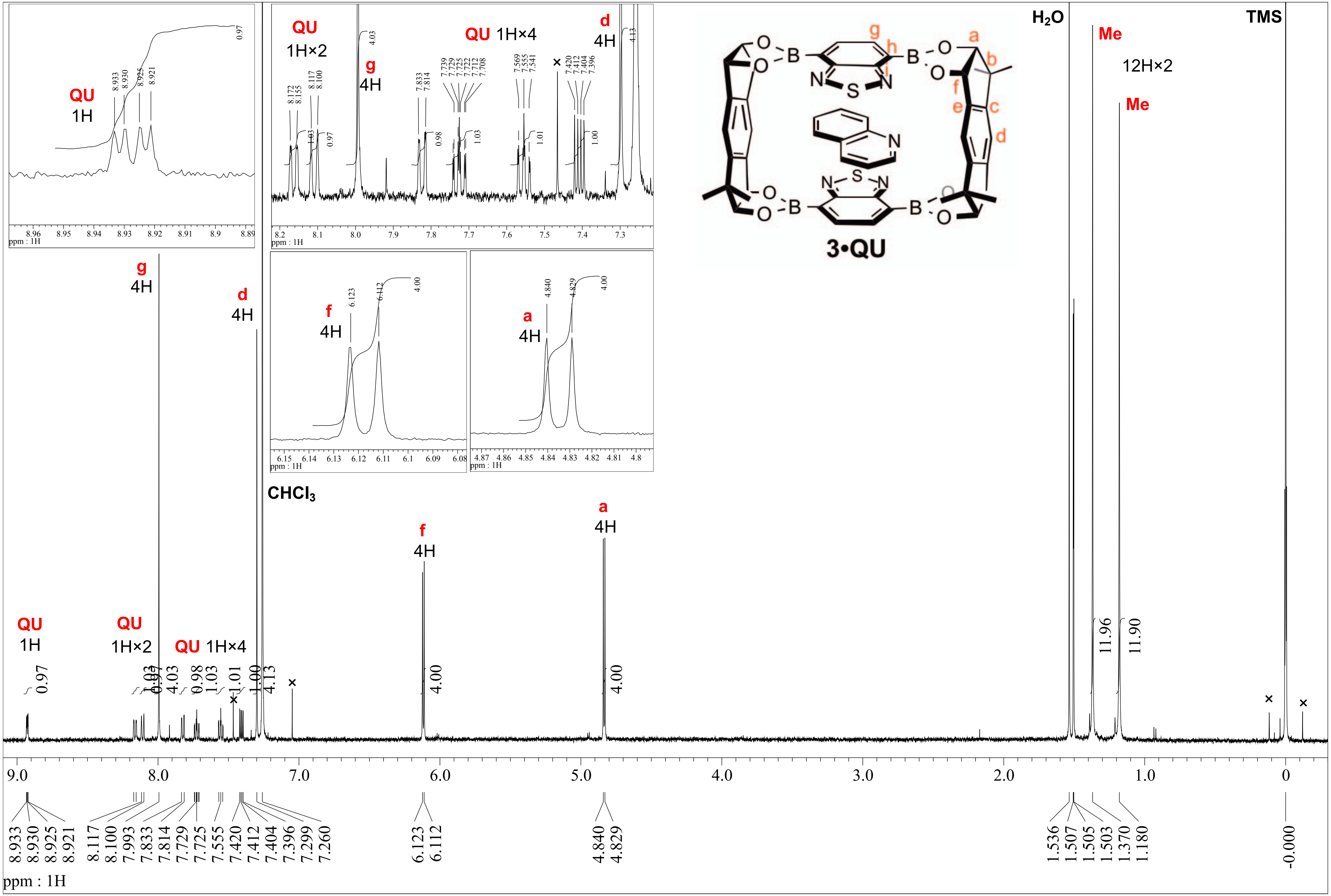

Supplement: Supplementary file 1 [file SC-007-C5SC04766H-s001.pdf]
